# Metabolism-related long non-coding RNA in the stomach cancer associated with 11 AMMLs predictive nomograms for OS in STAD

**DOI:** 10.3389/fgene.2023.1127132

**Published:** 2023-03-13

**Authors:** Wenjian Jin, Kongbo Ou, Yuanyuan Li, Wensong Liu, Min Zhao

**Affiliations:** ^1^ Department of Hepatopancreatobiliary Surgery, Changzhou First People’s Hospital, Third Affiliated Hospital of Soochow University, Changzhou, China; ^2^ Department of Urinary Surgery, The Third Affiliated Hospital of Soochow University, Changzhou First People’s Hospital, Soochow University, Changzhou, China; ^3^ Department of Gastrointestinal Surgery, The Third Affiliated Hospital of Soochow University, Changzhou First People’s Hospital, Soochow University, Changzhou, China; ^4^ Department of Gastrointestinal Surgery, Changzhou Maternal and Child Health Care Hospital, Changzhou Medical Center, Nanjing Medical University, Changzhou, Jiangsu, China

**Keywords:** gastric cancer, amino acid metabolism, long non-coding RNA, prognostic model, immunity

## Abstract

**Background:** The metabolic processes involving amino acids are intimately linked to the onset and progression of cancer. Long non-coding RNAs (LncRNAs) perform an indispensable function in the modulation of metabolic processes as well as the advancement of tumors. Non-etheless, research into the role that amino acid metabolism-related LncRNAs (AMMLs) might play in predicting the prognosis of stomach adenocarcinoma (STAD) has not been done. Therefore, This study sought to design a model for AMMLs to predict STAD-related prognosis and elucidate their immune properties and molecular mechanisms.

**Methods:** The STAD RNA-seq data in the TCGA-STAD dataset were randomized into the training and validation groups in a 1:1 ratio, and models were constructed and validated respectively. In the molecular signature database, This study screened for genes involved in amino acid metabolism. AMMLs were obtained by Pearson’s correlation analysis, and predictive risk characteristics were established using least absolute shrinkage and selection operator (LASSO) regression, univariate Cox analysis, and multivariate Cox analysis. Subsequently, the immune and molecular profiles of high- and low-risk patients and the benefit of the drug were examined.

**Results:** Eleven AMMLs (LINC01697, LINC00460, LINC00592, MIR548XHG, LINC02728, RBAKDN, LINCOG, LINC00449, LINC01819, and UBE2R2-AS1) were used to develop a prognostic model. Moreover, high-risk individuals had worse overall survival (OS) than low-risk patients in the validation and comprehensive groups. A high-risk score was associated with cancer metastasis as well as angiogenic pathways and high infiltration of tumor-associated fibroblasts, Treg cells, and M2 macrophages; suppressed immune responses; and a more aggressive phenotype.

**Conclusion:** This study identified a risk signal associated with 11 AMMLs and established predictive nomograms for OS in STAD. These findings will help us personalize treatment for gastric cancer patients.

## Introduction

Cancer rates have been rising at an alarming rate in recent years, particularly regarding gastrointestinal cancer, which is linked to a high rate of morbidity and mortality. As indicated by the “2020 Global Cancer Report” published by the World Health Organization, gastric cancer ranks fifth and fourth in incidence and mortality, respectively (Sung et al., 2021). Although events of both new cases and deaths from gastric cancer are falling worldwide, more than one million people suffer from this disease annually (Thrift and El-Serag, 2020).

Thus, it is urgent to continuously improve the diagnosis and prognosis evaluation system of gastric cancer. Many variables, such as the natural environment, lifestyle, infection, genetics, etc., may contribute to the onset and progression of tumors (Yang et al., 2021a).

The part played by metabolism in tumor onset and progression is another factor that has been progressively uncovered. Research has shown that metabolites, including tumor metabolites as clinical illness indicators, may alter DNA and protein modification *via* chemical modification and metabolite-macromolecular interactions, and that this is important for the regulation of DNA, RNA, and protein activities (Park et al., 2020). Abnormal changes in energy metabolism are an important sign of malignant tumors. Tumor cells can plunder energy and substrates for anabolism through metabolic reprogramming, thereby promoting their survival and rapid proliferation (Tarrado-Castellarnau et al., 2016). Glucose and fatty acid metabolic abnormalities are involved in carcinogenesis, metastasis, treatment resistance, and cancer stem cell survival (Park et al., 2020). Huang et al. also found that abnormal iron metabolism was significantly related to lymphohematopoietic tumors, which set off a research upsurge on iron metabolism-related targets, hoping to obtain more strategies for the treatment of lymphohematopoietic tumors. Amino acids, one of the three major nutrients, were also found to be intimately linked to the onset and progression of tumors. Ren et al. reported that amino acid metabolism is associated with colorectal cancer (Ren et al., 2022). Zhao et al. found that amino acid metabolism is linked to the prognosis of liver cancer and the immune landscape (Zhao et al., 2021). Nevertheless, the link between amino acid metabolism and gastric cancer, the second most common cancer of the digestive system, is yet to be thoroughly investigated.

Long non-coding RNA (LncRNA) is a type of RNA not involved in coding that is over 200 nt in length, and numerous studies on LncRNA have emerged in the past decade (Bridges et al., 2021). LncRNAs can regulate cell proliferation, differentiation, signal transduction, and inflammatory responses in the human body through different pathways, and participate in the development of various diseases including cancer (Chen et al., 2019), diabetes (Feng et al., 2017), and cardiovascular disease (Jin et al., 2021). Moreover, LncRNA is implicated in many different metabolic pathways and may affect posttranslational modifications of key metabolic enzymes in a direct or indirect manner (Bridges et al., 2021).

The study by Dai et al. found that LncRNAs related to amino acid metabolism were linked to the prognosis of breast cancer, (Dai et al., 2022) suggesting a novel approach to the therapy of this disease. However, there is currently insufficient data from studies to conclude that LncRNAs involved in amino acid metabolism are linked to the outcomes (prognosis) of patients suffering from gastric cancer. This research aimed to discover new gastric cancer therapeutic targets. Although the lack of tissue-specific expression patterns and sequence conservation makes LncRNA research more difficult, it also makes it more valuable.

The surrounding environment in which tumor cells live constitutes the tumor microenvironment (TME). TME factors, including immune cell infiltration, perform a crucial function in tumor onset and progression. Therefore, immunity is closely related to tumors. Not only that, but immune cell infiltration also determines the prognosis of patients with malignant tumors (Ge et al., 2019). Immune checkpoint inhibitors (ICPIs) are used in gastrointestinal tumors as a new form of immunotherapy (Jin et al., 2020). However, due to various reasons such as individual differences and tumor drug resistance, the role of ICPIs is still limited. Immune checkpoints are critical. Studies have shown that LncRNAs also play an important role in regulating immune responses, such as T cell development, differentiation and activation, as well as the production of inflammatory mediators (Heward and Lindsay, 2014). Amino acid metabolism can also affect the tumor immune microenvironment (TIME). A research found that cysteine can promote tumor cell proliferation, enhance their invasiveness, and inhibit T cell activity (Levring et al., 2012). Additionally, some studies have found that leucine (Hayashi et al., 2013), serine (Ma et al., 2017), and other amino acids can promote T cell activation and proliferation. Therefore, an in-depth study of the relationship between amino acid metabolism-related LncRNAs (AMMLs) and the prognosis and immunity of gastric cancer might offer fresh perspectives for the immunotherapy of this disease.

## Materials and methods

### Data acquisition and processing

The flow chart for the analysis of this study is shown in Supplementary Figure S1. This study retrieved and collected STAD-related expression and clinical data from the TCGA database. 374 amino acid metabolism-related genes were obtained from REACTOME_METABOLISM_OF_AMINO_ACIDS_AND_DERIVATIVES, which was contained in the Molecular Signatures Database v7.5.1 [GSEA | MSigDB (gsea-msigdb.org)]. In addition, the corresponding expression data of the 374 genes were obtained in the TCGA transcription data. AMMLs were screened based on Pearson’s correlation analysis with a filter condition of *p* < 0.001 and |correlation coefficient|>0.5. Then, utilizing the “limma” R package, based on FDR<0.05 and |logFC|>1, differential analysis was performed between gastric cancer samples and normal samples to obtain differentially expressed LncRNAs.

### Risk establishment and verification of signatures

Based on AMMLs differentially expressed in gastric cancer samples and tumor samples. First, This study conducted univariate Cox regression analysis to identify 24 AMMLs linked to overall survival (OS) in STAD (*p* < 0.05). These 24 AMMLs were then subjected to LASSO analysis. A total of 11 characteristic AMMLs and their correlation coefficients were obtained. These 11 AMMLs were used to determine the patient’s risk score. The calculation formula is shown below: Risk Score = S (Expi ∗ Coefi). After that, patients were categorized into high- and low-risk groups (categories) as per their median score. Kaplan-Meier (K-M) analysis, log-rank test, and time-dependent receiver operating characteristic (ROC) curve analysis were conducted with the “survival,” “survivaler” and “survivalROC” R packages to judge the OS of different risk groups, and the accuracy of the prognostic model. Furthermore, This study employed univariate and multivariate Cox regression analyses to verify if AMMLs-related risk scores independently functioned as prognostic indicators for STAD. To confirm the efficacy of this prognostic model, This study computed a risk score by applying similar regression coefficients, formulas, and genes for both the validation and the combined cohorts. Additionally, This study explored the robustness of the model in an integrated group classified based on different clinical traits (age, sex, grade, etc.).

### Co-expression network

To determine the link between AMMLs and mRNA, This study constructed an mRNA-LncRNA co-expression network model using Cytoscape_v3.9.1, a network visualization software.

### Nomogram

The findings of the multivariate analysis were utilized in the development of nomograms for anticipating one-, three-, and five-year survival rates. The “rms” R program was adopted to construct and illustrate the findings. Values of discriminant performance and prediction nomograms were determined by Harrell’s C-index and calibration curve.

### Gene set enrichment analysis (GSEA)

First, the “limma” R program was implemented to detectdifferentially expressed genes (DEGs) between high- and low-risk categories, and the screening conditions were *p* < 0.05 and |LogFC|>1. After that, the “clusterProfiler” R program was employed to conduct gene enrichment analysis based on Gene Ontology (GO). An FDR value of <0.05 was required for the pathway and function enrichment analysis to be deemed significant. Next, GSEA software (version 4.2.3) was employed to evaluate the “c2. cp.kegg.v7.5. symbols.gmt” gene set in low- and high-risk categories to determine which genes were enriched. This study screened enrichment results with a nominal *p*-value <5% and FDR <25%.

### Immune-related features

This study started by using the ESTIMATE technique to derive each patient’s immune and stromal scores. This study then explored the differences (variations) in immune, stromal, and ESTIMATE scores in the TIME of STAD patients across low- and high-risk categories. This study next used a single-sample GSEA (ssGSEA) algorithm to compare the immune functions of patients across low- and high-risk categories and elucidate the correlation between the risk score and the TIME in STAD patients. Subsequently, This study explored the level of immune cell infiltration in high- and low-risk groups through different algorithms such as CIBERSORT, EPIC, QUANTISEQ, and XCELL. Since immune checkpoint inhibitors are widely used in tumor therapy, This study compared the levels of various widely used immunosuppressors and immune checkpoints across high- and low-risk categories. These immune checkpoints and immunosuppressive factors were obtained from previously published articles.

### Analysis of drug sensitivity

To evaluate targeted drugs for different risk groups and sensitivity to chemotherapeutics, This study predicted the maximal inhibitory concentration (IC50) with the help of the “pRRophetic” R package.

### Statistical analysis

This study used R soft 4.1.2 software to analyze all the data. Pearson correlation analysis was conducted to study the co-expression of amino acid metabolism genes and LncRNA. The prognostic factors were determined by LASSO regression analysis. This study used univariate and multivariate COX regression analyses to ascertain if the risk score independently acted as a predictive marker for STAD. An evaluation of the risk model’s specificity and sensitivity was executed using the area under the ROC curve (AUC). Categorical variables were subjected to a comparison with the chi-square test and the Fisher’s exact test. To compare data of factors between risk groups, a Student’s t-test was employed.

## Result

### Data sources and basic clinical information

In total, 407 mRNA expression profiles were acquired from TCGA, comprising 375 tumors and 32 normal tissue samples. The clinical data of STAD samples, including age, gender, grade, TNM stage, etc. 374 genes involved in the metabolism of amino acids were obtained from REACTOME_METABOLISM_OF_AMINO_ACIDS_AND_DERIVATIVES, in the Molecular Signatures Database. The list is presented in Supplementary Table S1.

### Screening for AMML differentially expressed in normal and gastric cancer samples and associated with prognosis

**FIGURE 1:**
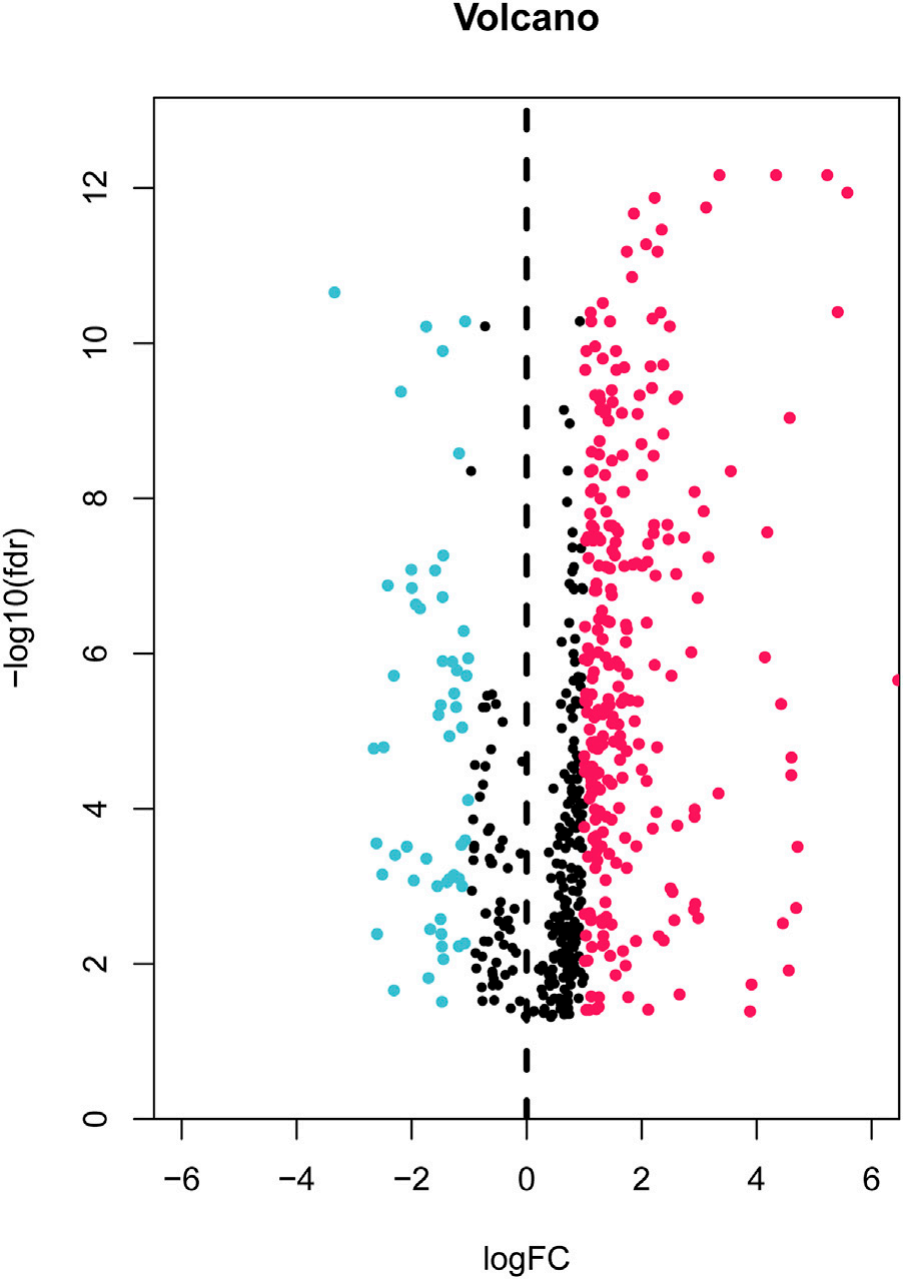
Volcano plot of 327 differentially expressed AAMRLs in STAD. AAMRLs, Amino acid metabolism-related LncRNAs; STAD, Stomach adenocarcinoma.

**FIGURE 2:**
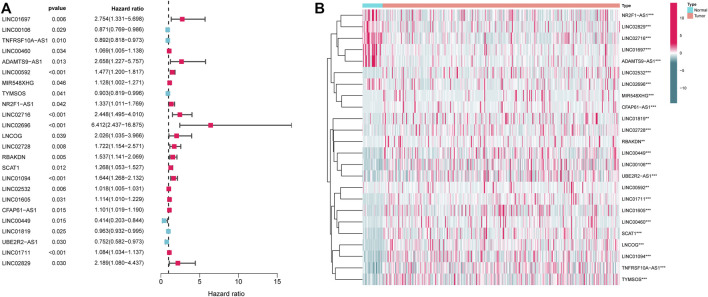
Screening with AMMLs. **(A)** Forest plot of AMMLs linked to STAD OS. **(B)** Heatmap of differential expression of AMMLs linked to STAD OS. AMMLs, Amino acid metabolism-related LncRNAs; STAD, Stomach adenocarcinoma; *p < 0.05; **p < 0.01; ***p < 0.001.

First, the expression data of 374 amino acid metabolism-related genes were extracted from the TCGA database. Then, relevant LncRNAs were screened based on Pearson correlation analysis, and the screening conditions were |correlation coefficient (r)|>0.4 and FDR<0.05. In total, 1724 AMMLs were obtained (Supplementary Table S2). Subsequently, 327 AMMLs with differential expression were identified by performing differential analysis between normal and tumor tissues utilizing the “Limma” R package (Supplementary Table S3). Differential AMMLs are shown on a volcano plot in Figure 1. Then, 24 AMMLs (*p* < 0.05) linked to OS were obtained as per the univariate COX regression analysis of 327 AMMLs. The forest map shown in Figure 2A displays the HR values and confidence intervals (CI) for the 24 AMMLs, whereas the heat map in Figure 2B shows the specific variations in expression.

### Development and verification of a predictive model based on AMMLs

**FIGURE 3:**
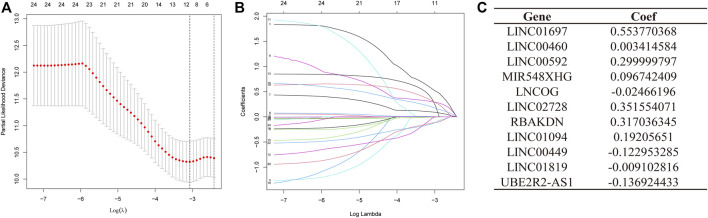
LASSO Regression for STAD Patient Risk Models Based on AMMLs. **(A)** 10-fold cross-validation of variable selection in LASSO models. **(B)** The distribution of LASSO coefficients for 11 AMMLs. **(C)** 11 characteristic AMMLs and their correlation coefficients. LASSO, Least absolute shrinkage and selection operator; STAD, Stomach adenocarcinoma; AMMLs, Amino acid metabolism-related LncRNAs.

A total of 371 samples were obtained after merging expression data with survival data. Patients were randomized into test and training sets on a 1:1 ratio. The sample size for the training set was 187, whereas the value for the testing set was 184. Then, based on the 24 AMMLs obtained in the training cohort, the LASSO regression algorithm was employed to correct overfitting and underfitting in the training group. Finally, 11 stable AAMLs were obtained as the best LncRNAs for prognostic models, as shown in Figures 3A, B. The specific 11 AMMLs and the corresponding correlation coefficients are depicted in Figure 3C. The risk score was calculated as follows: Riskscore = LINC01697 × 0.553770368482515 + LINC00460 × 0.00341458368213084 + LINC00592 × 0.29999979722043 + MIR548XHG × 0.0967424091144317+ LNCOG× −0.0246619595544397 + LINC02728 × 0.351554070674873 + RBAKDN ×0.317036345024823 + LINC01094× 0.192056510214801 + LINC00449 × −0.122953284509337 + LINC01819 × −0.00910281613561449 + UBE2R2-AS1 × −0.136924432797998

### Get its risk score

**FIGURE 4:**
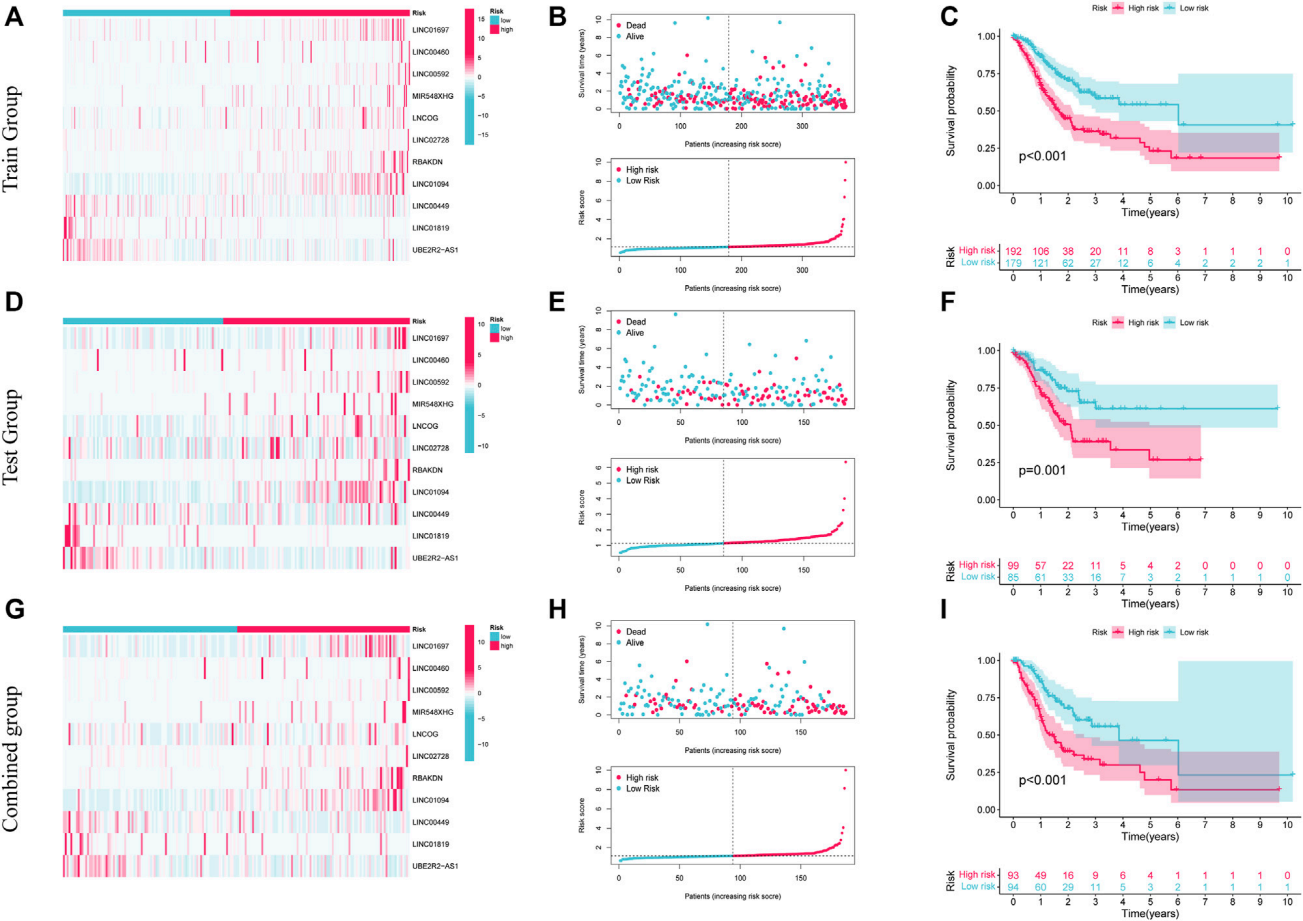
The prognostic significance of 11 AMMLs models in testing, training, and whole cohorts. **(A,D,G)**. Survival curves of two groups of patients in training, test, and the whole group. **(B,E,H)**. Model presentation of AMMLs with survival status and time based on testing, training, and the full set of risk scores. **(C,F,I)**. Expression of 11 AMMLs in training, test and comprehensive cohorts in high and low-risk categories. AMMLs, LncRNAs related to amino acid metabolism.

Patients in the low- and high-risk categories were divided premised on the medium risk score. Figure 4A shows the expression of 11 AAMLs in STAD patients, with LINC01697, LINC00460, LINC00592, MIR548XHG, LNCOG, LINC02728, RBAKDN, and LINC01094 being expressed at a high level in the high-risk group, whereas LINC00449, LINC01819, and UBE2R2-AS1 were expressed at a low level, of course, This needs to be verified in experiments. This study observed that patients with higher risk scores for STAD had a lower likelihood of survival, as depicted in Figure 4B. K-M survival analysis found that high-risk patients (*p* < 0.001) had a considerably shortened OS duration, as depicted in Figure 4C. Similar patterns of expression, risk, and survival were observed between the test and composite groups and the training set (Figures 4D, E, G, H). Furthermore, high-risk individuals had substantially shorter OS duration in both the test (*p* = 0.001) and combined groups (*p* < 0.001). These all validate the accuracy of this prognostic model. This study further explored the precision of prognostic features through the ROC curve. The predicted AUC values for 1-, 3-, and 5- years in the test group were 0.723, 0.638, and 0.667, correspondingly, and the predictive power of the prognostic model was significantly better than that of age (0.544), sex (0.532), and grade (0.560). and staging (0.579), as shown in Supplementary Figures 2A, D. Also, this finding was also consistent in the test set and the combined set (Supplementary Figures 2B, C, E, F).

This study classified patients according to their clinical characteristics to further examine the link between risk scores and patient prognosis. Patients in the high-risk category were shown to have a dismal prognosis across all demographics, including different ages (>65 and ≤65 years), genders (female and male), grades (G1-2 and G3), and stages (I-II and III-IV). The low-risk category also exhibited improved survival status in contrast with that of the high-risk category across different N stages (N0, and N1-N3). In M and T stages, M0 and T3-4 showed the same performance as above, and the OS of the low-risk category was elevated. Details are shown in Supplementary Figures 3A–G, I–L, N Conversely, the difference in survival across the two groups in terms of M1 and T1-2 was insignificant; this could be because of the small sample sizes in these groups, as depicted in Supplementary Figures 3H, M. As per these findings, the prognostic model is highly accurate and stable.

**FIGURE 5:**
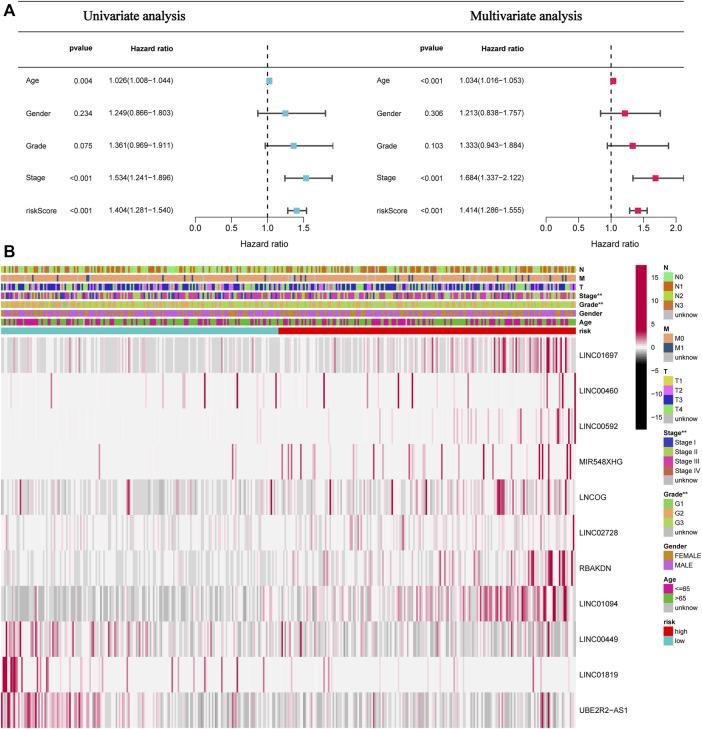
The risk score independently functions as a prognostic marker in STAD patients. **(A)**. Univariate and multivariate overall survival prognostic analysis of clinical parameters and risk scores. **(B)**. Heatmap of prognostic features and clinicopathological findings in STAD AMMLs. *p < 0.05; **p < 0.01; ***p < 0.001.

This study next conducted univariate and multivariate analyses to investigate if the risk model had any impacts on the prognostic factors of patient survival. Univariate analysis results illustrated that age (*p* = 0.004), stage (*p* < 0.001), and Risk score (*p* < 0.001) can affect the prognosis of STAD patients. The multivariate analysis illustrated that age (*p* < 0.004), stage (*p* < 0.001), and Risk score (*p* < 0.001) independently acted as prognostic indicators for patients (Figure 5A). Furthermore, as depicted in Figure 5B, tumor grade and stage differed between high- and low-risk patients.

### Co-expression network of amino acid metabolism-associated mRNAs and LncRNAs

**FIGURE 6:**
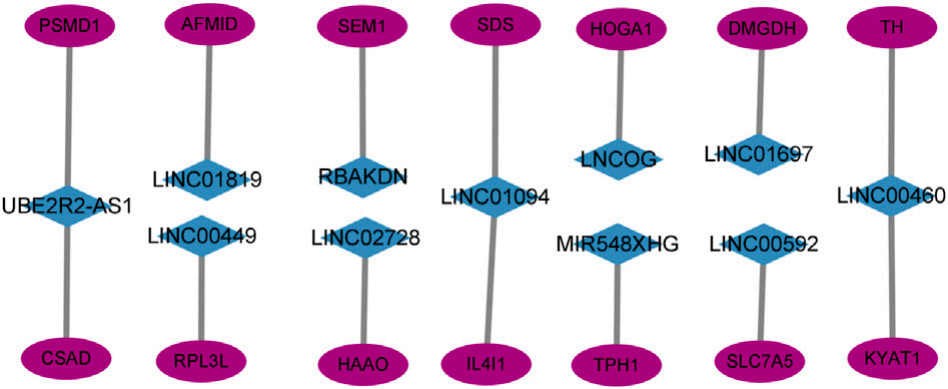
Co-expression network of 11 AMMLs and genes involved in the metabolism of amino acids. AMMLs, Amino acid metabolism-related LncRNAs.

This study created and visualized an mRNA-lncRNA co-expression network in Cytoscape to additionally observe the links between genes involved in amino acid metabolism and 11 AAMLs (version 3.9.1, http://www.cytoscape.org/), as depicted in Figure 6.

### Creation of predictive nomograms

**FIGURE 7:**
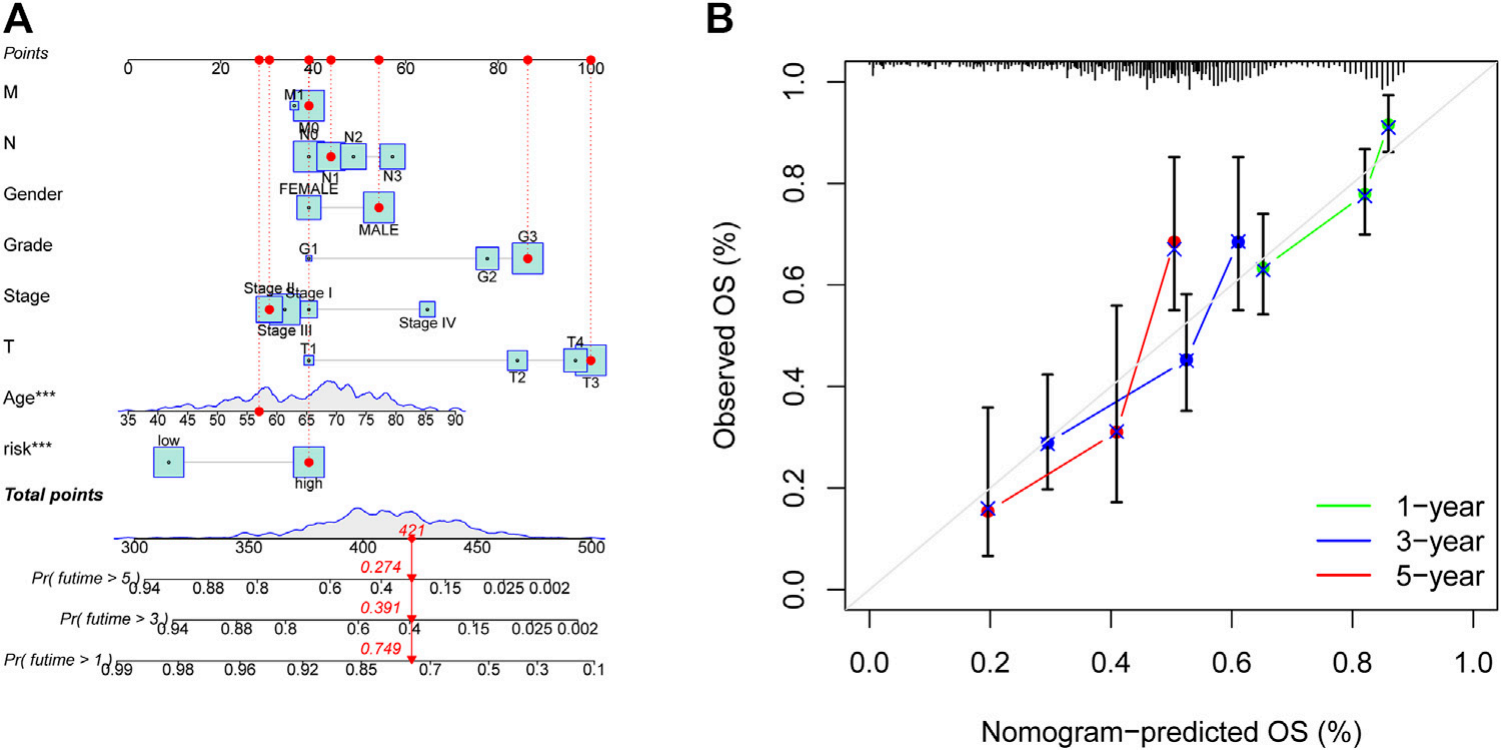
Nomogram for anticipating OS in GC patients. **(A)** Nomogram of prognostic models and clinicopathological factors founded on 11 AMMLs. **(B)** Performance of the nomogram model determined by the calibration curve. The ideal nomogram is shown as the diagonal dotted grey line. *p < 0.05, **p < 0.01; ***p < 0.001. OS, overall survival; GC, gastric cancer; AMMLs, Amino acid metabolism-related LncRNAs.

This study designed the nomogram incorporating patients’ features and demonstrated its accuracy using Harrell’s concordance index (C-index) and a calibration curve, which showed consistency between the nomogram’s predicted and actual survival over 1, 3, and 5 years (Figure 7).

### Enrichment analysis

**FIGURE 8:**
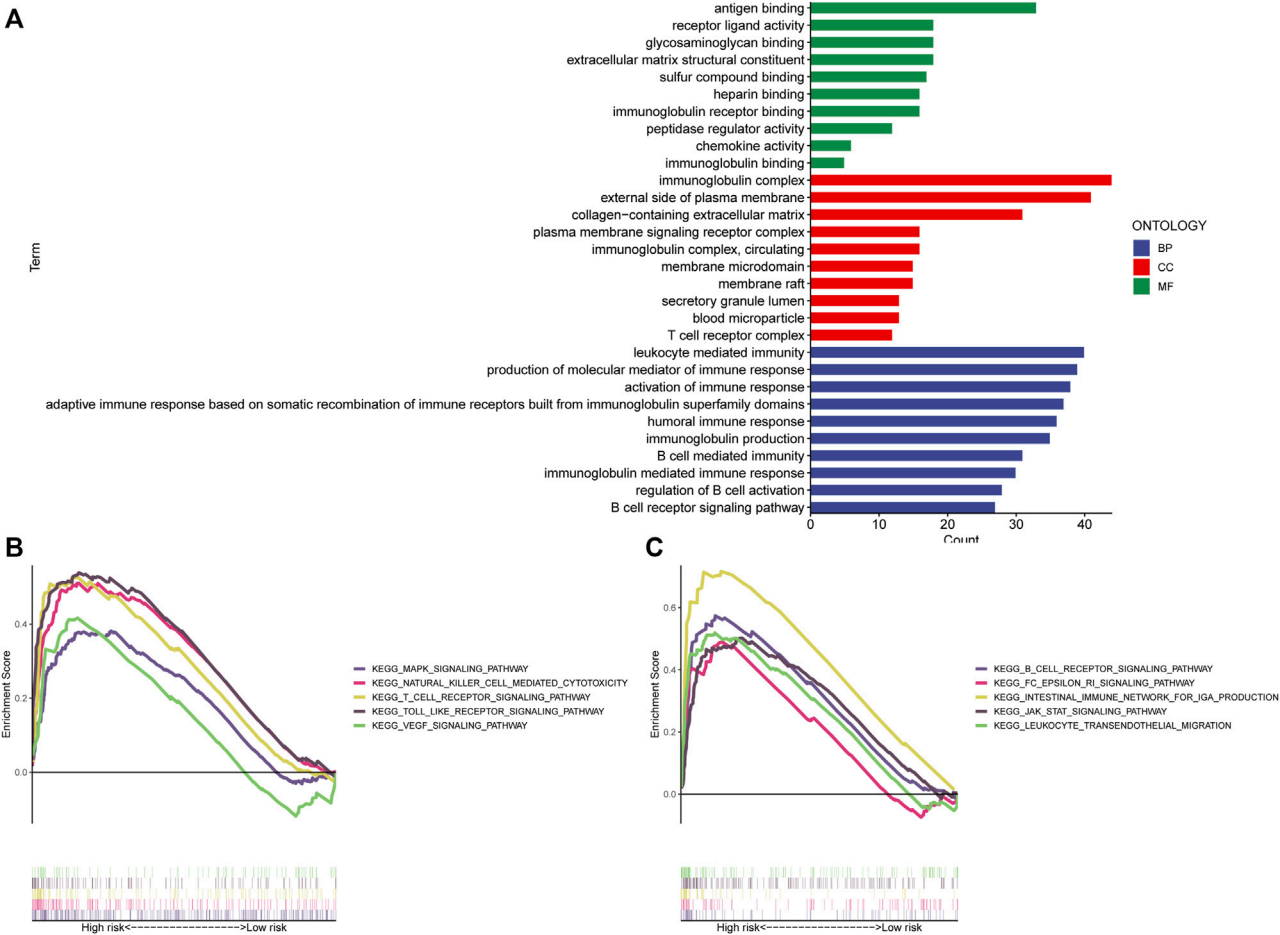
Enrichment analysis. **(A)** GO analysis shows the enrichment of most immune-associated biologic processes. **(B,C)** GSEA analysis shows enriched pathways in high-risk groups. GSEA, gene set enrichment analysis; LncRNAs, long non-coding RNAs; GO, Gene Ontology; MF, molecular function; CC, cellular component; BP, biological process; KEGG, Kyoto Encyclopedia of Genes and Genomes.

The “limma” R program was first used to screen for DEGs between high and low-risk categories. The screening conditions were *p* < 0.05 and |LogFC|>1, and 284 DEGs were screened. The “clusterProfiler” program in R was subsequently employed to conduct a GO enrichment study. Figure 8A demonstrates that the majority of these genes undergo enrichment in immune-related functions. This study utilized GSEA to contrast high- and low-risk STAD patients for the enrichment of biologically functioning pathways to better understand the differences between the two groups. *p*-values <5% and FDRs <25% were considered meaningful enrichments. A considerable enrichment in the high-risk patients was found in tumor proliferation, angiogenesis, and tumor resistance-related pathways, such as VEGF SIGNALING PATHWAY, MAPK SIGNALING PATHWAY, and JAK-STAT SIGNALING PATHWAY which may lead to faster tumor progression. Moreover, immune-related pathways such as those involving T cell receptors, B cell receptors, the generation of IGA in the gut, and natural killer cells, were all considerably enriched (Figures 8B, C). Furthermore, there were no substantially enriched pathways discovered in the low-risk population. This difference between the two categories could be the factor that led to the reduced survival rate of the high-risk patients.

### Immune correlation analysis

**FIGURE 9:**
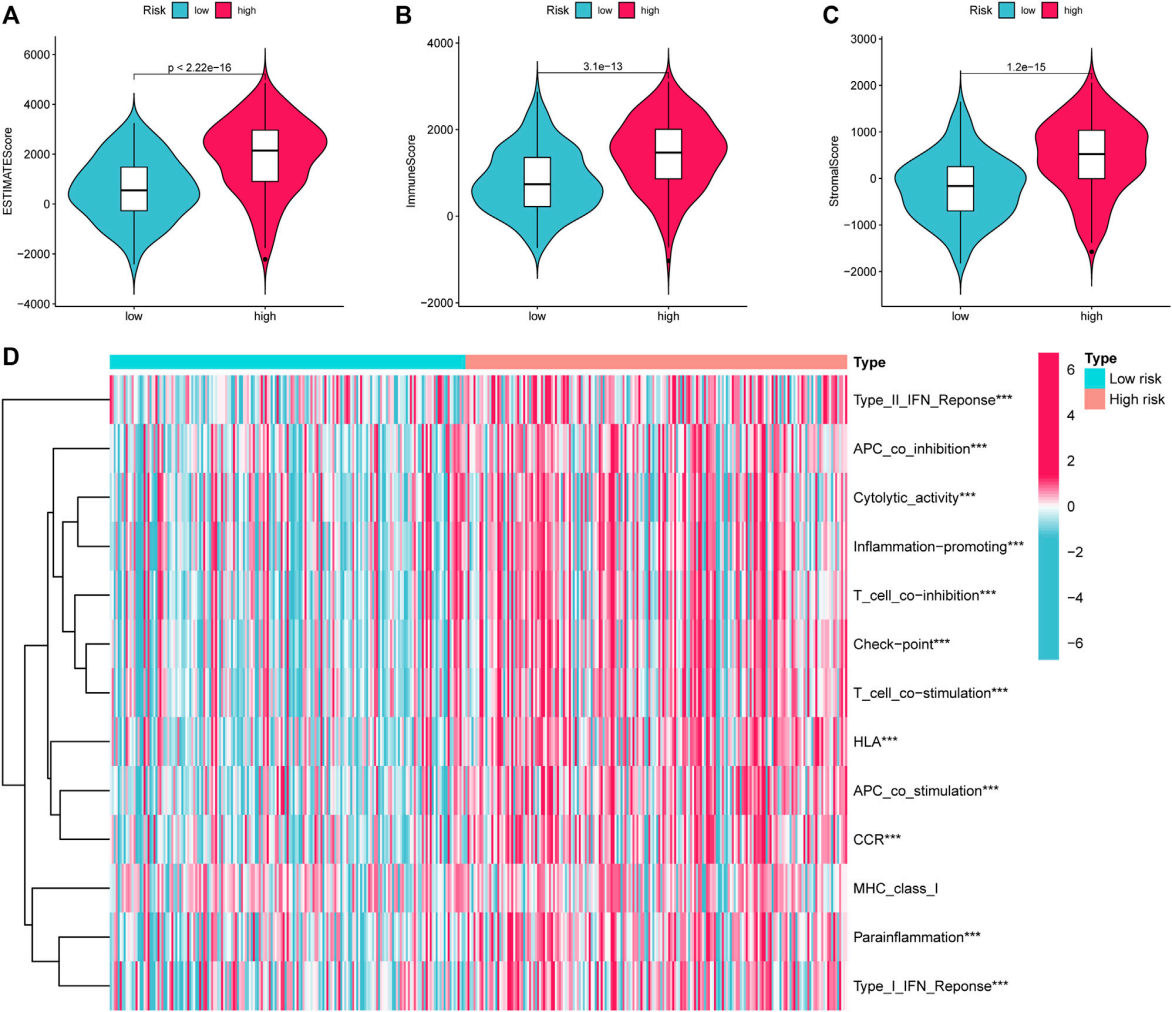
ESTIMATE calculates the purity of STAD. **(A)** Estimate Score **(B)**. Immune Score **(C)**. Stromal Score. **(D)** Variations in immune function across high and low-risk categories calculated by ssGSEA algorithm. STAD, Stomach adenocarcinoma; ssGSEA, single-sample gene set enrichment analysis. *p < 0.05; **p < 0.01; ***p < 0.001.

**FIGURE 10:**
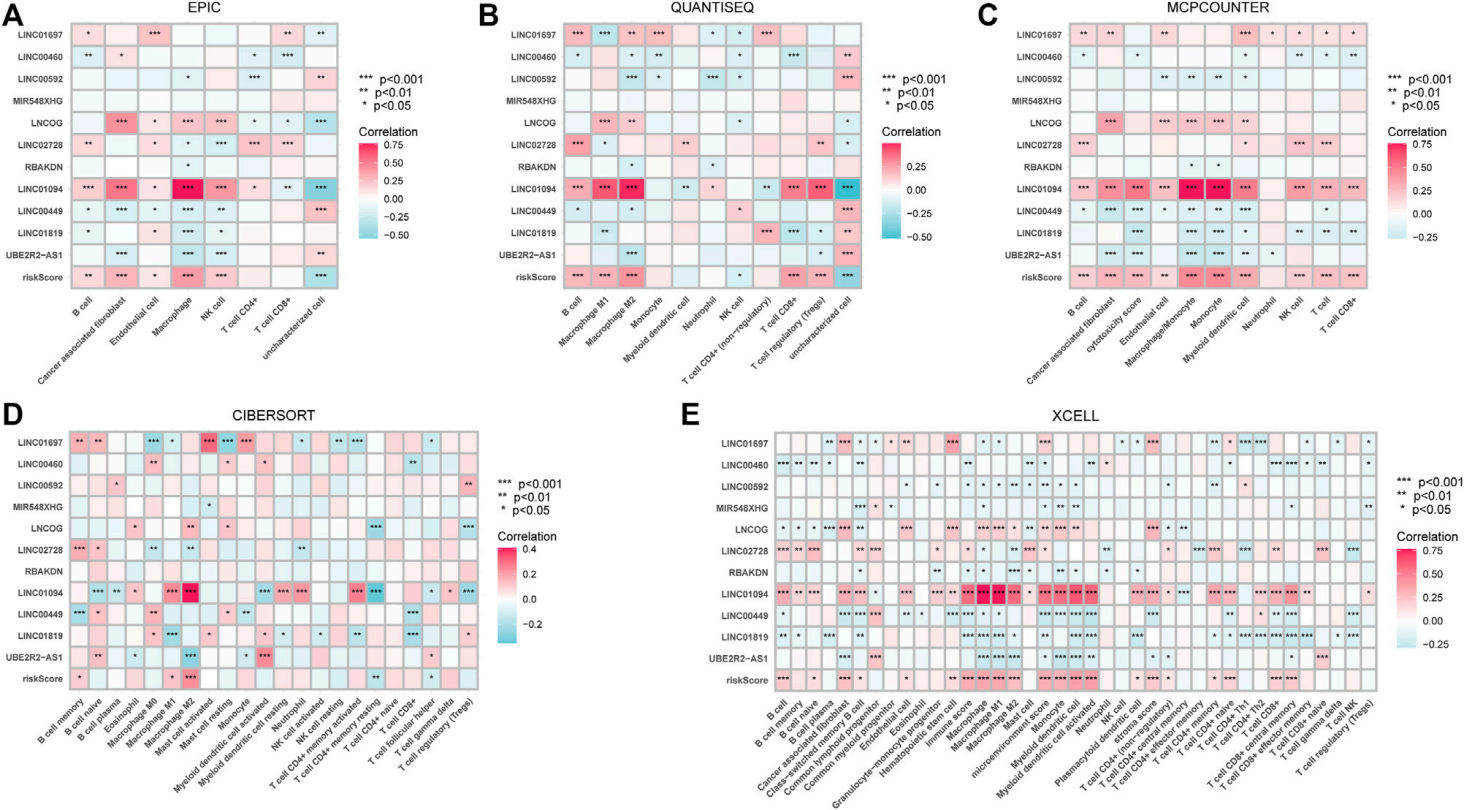
The relationship between immune cell infiltration and risk score in the tumor immune microenvironment of STAD patients calculated by different algorithms. **(A)** EPIC **(B)**. QUANTISEQ **(C)**. MCPCOUNTER **(D)**. CIBERSORT **(E)**. XCELL. *p < 0.05; **p < 0.01; ***p < 0.001.

**FIGURE 11:**
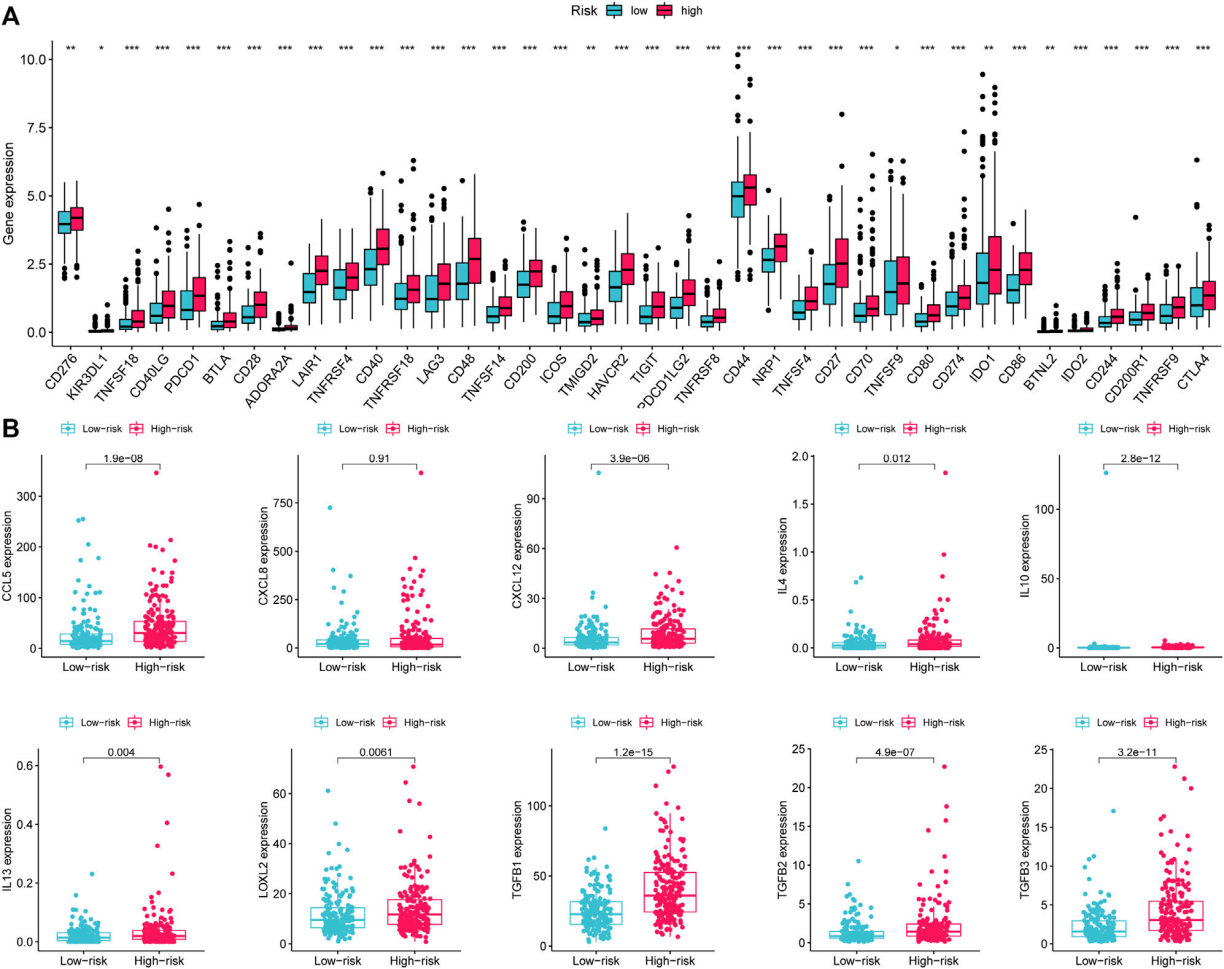
Variations in the expression of immune-associated genes between low- and high-risk groups. **(A)** Expression of immune checkpoints in high- and low-risk patients with gastric cancer. **(B)** Expression of immunosuppressive cytokines across the two groups. *p < 0.05; **p < 0.01; ***p < 0.001.

Given that the high-risk category was shown to have an enrichment of immune-related pathways, This study investigated how prognostic models and immunity are linked with each other. This study first used the ESTIMATE method to assess the matrix and immune scores of the patient’s immune milieu. Results showed that high-risk individuals’ matrix, immune, and comprehensive scores were all substantially elevated and different from those of the low-risk individuals, implying that the immune microenvironment of the two groups was significantly different (Figures 9A–C). This study next used ssGSEA to determine whether there were any remarkable differences in immune functions between the two groups and discovered that 12 immune processes, including Types I and II IFN Response, and APC co-inhibition, were substantially upregulated in the high-risk patients (Figure 9D). This study also used algorithms like MCPCOUNTER, CIBERSORT, EPIC, QUANTISEQ, and XCELL to investigate immune cell infiltration across distinct risk categories. Figure 10 shows that the patient’s risk scores were positively linked to fibroblasts, B cells, M1and M2 macrophages, and Tregs cells, but inversely linked to uncharacterized cells. Variations in immunocyte infiltration between high- and low-risk categories could be a key factor in the diverse outcomes observed between them. Due to the widespread use of immune checkpoints in tumors, This study investigated whether high-risk patients might gain more benefit from immune checkpoint inhibitors by comparing their levels of checkpoint expression with those of low-risk patients. The increased expression of immune checkpoints in the high-risk category compared to the low-risk population implies that immune checkpoint inhibitors may be very effective in high-risk patients. (Figure 11A). Since elevated levels of Tregs and macrophage infiltration in the high-risk population secrete some immunosuppressive factors, we explored to verify the expression levels of these immune factors in the high-risk group versus the low-risk group. It was found that the expression levels of cytokines (IL4, IL10, IL13, TGFB1, TGFB2, TGFB3, etc.) were higher in the high-risk group, which further suggests that elevated levels of Tregs and macrophage infiltration in the high-risk population play a suppressive role in the immune microenvironment. (Figure 11B). These data suggest that the high-risk patients exhibit indolence in tumor immunity, which might account for their poor prognosis.

### Analysis of drug sensitivity

**FIGURE 12:**
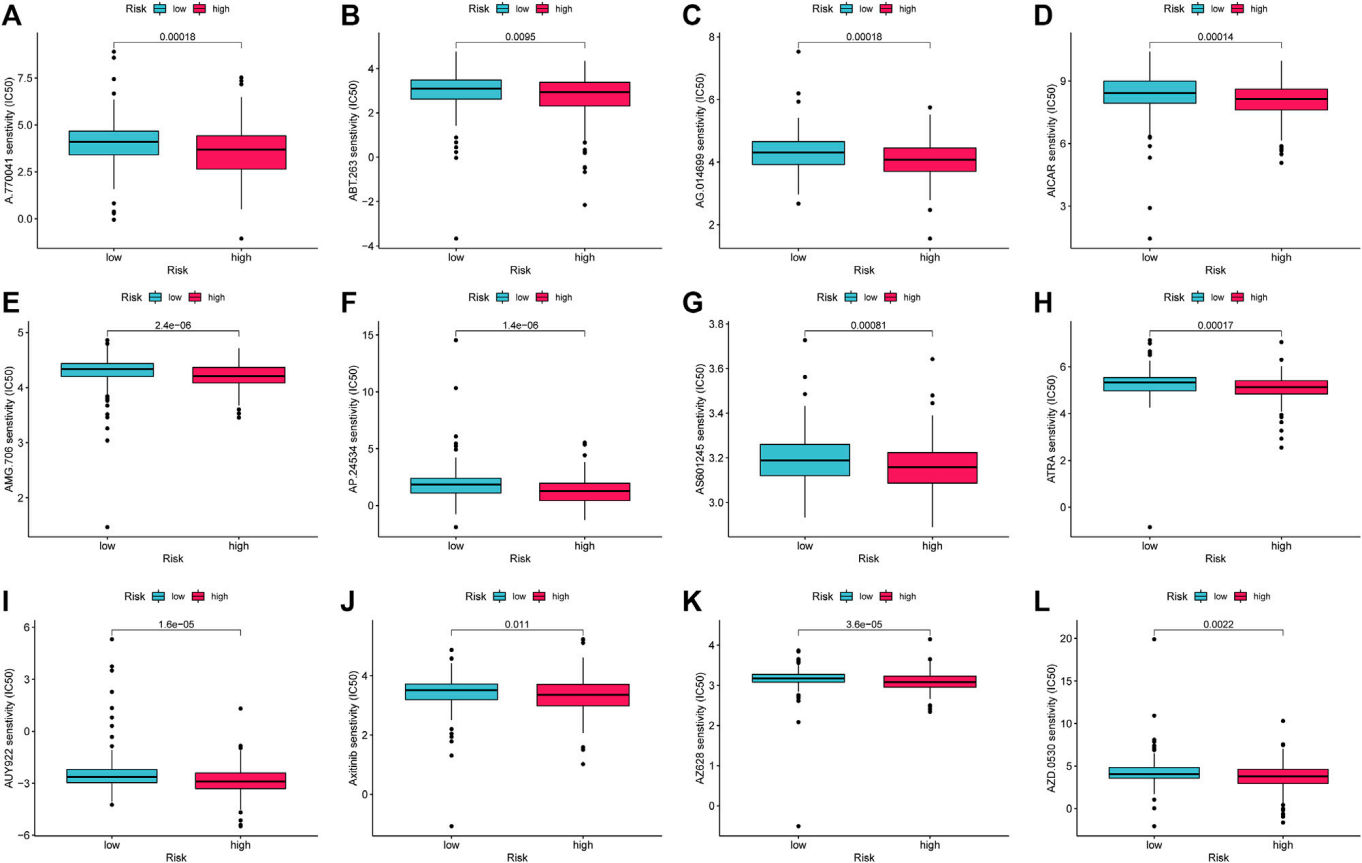
Drug candidates targeting AMMLs. IC50s of **(A)**. **(A)**770041 **(B)**. ABT.263 **(C)**. AG014699 **(D)**. AICAR **(E)**. AMG.706 **(F)**. AP.24534 **(G)**. AS601245 **(H)**. ATRA **(I)**. AUY922 **(J)**. Axitinib **(K)**. AZ628 and **(L)**. AZD.0530 between the low- and high-risk patients. AMMLs, LncRNAs related to amino acid metabolism, *p < 0.05; **p < 0.01; ***p < 0.001.

This study evaluated treatment response premised on IC50 of each sample using the pRRophetic algorithm to compare the sensitivity of prospective medications typical of AMMLs between high- and low-risk categories. Twelve targeted drugs, including A.770041, ABT.263, AG.014699, AICAR, AMG.706, AP.24534, AS601245, ATRA, AUY922, Axitinib, AZ628, and AZD.0530, were shown to be more effective in high-risk categories. This may provide insights into new treatment options for STAD patients (Figure 12A).

## Discussion

Under normal physiological conditions, the body maintains a dynamic balance of metabolism, but this metabolic state will be altered to some degree in a disease state. Therefore, detecting the metabolic level of the corresponding substance can help diagnose and judge the disease. Amino acids, as one of the main nutrients in the human body, have recently been the subject of substantial research in the field of oncology. Studies have found that amino acids may promote tumor progression. For example, when extracellular cysteine is reduced or lacks endogenous transsulfuration activity, cysteine production can support tumor cell proliferation *in vivo* (Zhu et al., 2019). It is well known that tumor cells grow rapidly in the early stage of tumors, but tumor angiogenesis is insufficient, and tumor cells obtain more energy through glycolysis, this phenomenon is called the “WarburgEffect” in this study. However, in the case of glucose starvation, tumor cells may obtain energy through autophagy. Tumor cell-related autophagy often yields amino acids and other metabolites, and new research suggests that non-essential amino acids may replace glucose as tumor cells’ functional substances (Uneyama et al., 2017). Amino acids have also been extensively studied in gastric cancer. Wang et al. disclosed that apoptosis was promoted in gastric cancer cells by the deprivation of glucose, whereas amino acids directly counteracted this effect (Wang et al., 2014). According to certain studies, the plasma amino acid level may distinguish between gastric ulcers and gastric cancer. Among them, the content of glutamine, histidine, arginine, and tryptophan in the plasma of gastric ulcer patients was elevated as opposed to that of tumor patients, while the content of ornithine was the opposite (Jing et al., 2018).

Liu et al. (2018) found that free amino acids in gastric juice can help the diagnosis of early gastric cancer. Abnormal metabolism of tumor cells can cause alterations in the TME, which in turn influences immune cell infiltration and promotes tumor immune escape. Numerous recent studies have shown that aberrant amino acid metabolism in patients with tumors might alter the TIME. Also, studies have found that a variety of amino acids or their transporters can promote T cell activation and proliferation, including leucine (Hayashi et al., 2013), methionine (Sinclair et al., 2019), serine (Ma et al., 2017), alanine (Ron-Harel et al., 2019), and so on. Research has also discovered that arginine deficiency in tumors not only leads to the anti-tumor response of T cells but also induces the generation of myeloid-derived suppressor cells (MDSCs) (Fletcher et al., 2015). In addition, T lymphocytes differentiate into Tregs but not into helper T cells in the presence of glutamine deficiency (Klysz et al., 2015). All of these things point to the significance of amino acid metabolism in the TIME. At present, amino acid metabolism-related factors are also effective targets for the treatment of tumors. For example, glutaminase inhibitor CB-893 (Johnson et al., 2018), glutamine metabolism inhibitor JHU083 (Leone et al., 2019), and arginase 1 inhibitor INCB001158 (Steggerda et al., 2017) can not only inhibit tumor progression but also increase the immune system in TME cell infiltration. However, there is no report on the value of AMMLs in assessing immune infiltration and clinical outcomes in gastric cancer. LncRNAs related to amino acid metabolism remodel the TME, and whether this change affects the prognosis and immunotherapy response of STAD patients is of great significance for us to explore.

So far, this is the first research project that uses AMMLs to establish a prognostic model in STAD. Pearson correlation and univariate Cox regression analyses can effectively detect cellular senescence LncRNAs linked to disease prognosis predicated on the RNA-seq data set downloaded from TCGA and genes related to amino acid metabolism. The LASSO regression technique was used to develop a prediction model that included 11 AMMLs. In this research, 371 samples were randomized into the training and test sets, of which the training set and the test set contained 187 and 184 samples, correspondingly. The prognostic model was proven to be reliable across all three study groups (training, test, and whole groups).

The 11 AMMLs used to construct prognostic models, included 7 risk genes notably, LINC01697, LINC00460, LINC00592, MIR548XHG, LINC02728, RBAKDN, and LINC01094, and 4 protective genes, namely LINCOG, LINC00449, LINC01819, and UBE2R2-AS1. Furthermore, This study found that some of these 11 LncRNAs have been previously reported in tumors. For example, LINC01697 has a diagnostic and prognostic function in lung adenocarcinoma and oral squamous cell carcinoma (Liu et al., 2019; Li et al., 2020). LINC00460 has been reported in various tumors such as head and neck squamous cell carcinoma (Yang et al., 2021b), cervical cancer (Lin et al., 2020), bladder cancer (Li et al., 2021), colorectal cancer (Ruan et al., 2021), and is implicated in tumor proliferation, migration, mesenchymal transition, drug resistance, and increased tumor progression (Meng et al., 2020; Cheng et al., 2021). Wang and Yang et al. also found that LINC00460 could promote the progression of gastric cancer (Wang et al., 2018; Yang et al., 2020). Xu et al. reported that the lncRNA UBE2R2-AS1 targeted the miR-877-3p/TLR4 axis, thereby promoting apoptosis in glioma cells (Xu et al., 2021). In colorectal cancer, upregulated long intergenic non-protein-coding RNA 1094 (LINC01094) is associated with a dismal prognosis and altered cellular function (Zhang et al., 2022). The RBAKDN gene has been linked to the prognosis of patients with cervical cancer. (Ye et al., 2021). Zhang et al. (2020) discovered that lung adenocarcinoma recurrence was linked to LINC01819. Previous studies have found that LINC00592 (Cheng et al., 2019) LINC02728 (Lai et al., 2021) LINC00449 (Zhang et al., 2021) are associated with gastric cancer prognosis. Non-etheless, reports of MIR548XHG are rare, only Shan et al. (2022) have found significant increase in MIR548XHG expression in endometriosis. Therefore, more research is needed in this area.

Pathways associated with B cell receptors, T cell receptors, and natural killer cell-mediated cytotoxicity were shown to be predominantly enriched in the high-risk category as per the GSEA analysis. Neither the impact of LncRNAs involved in amino acid metabolism nor their links to the immune milieu in gastric cancer has been studied. As a first step, the tumor purity of STAD patients in the high and low-risk categories was determined utilizing the ESTIMATE method. High-risk patients had greater values for all three scores (immune, stromal, and estimate scores) compared to those at lower risk. In addition, 12 out of 13 immune functions were different between the two groups, with the high-risk patients showing considerably higher immune function. The infiltration levels of immune cells in the STAD TME were subsequently investigated utilizing algorithms like CIBERSORT, EPIC, MCPCOUNTER, QUANTISEQ, and XCELL, and it was discovered that cancer-associated fibroblasts (CAFs), M2 macrophages, and Tregs cells were positively linked to risk scores. Previous studies have found that CAFs are associated with tumor size, tumor invasion depth, and metastasis (Quail and Joyce, 2013). CAFs typically secrete CXCL12, TGF-β, LOXL2, HGF, and IL-22 to promote tumor progression. This is consistent with the results of this study showing increased cytokine secretion of CAFs in high-risk patients. Tumors may manifest as cells that affect tumor growth, invasion, and metastasis by secreting these cytokines in gastric cancer. This negatively impacts the prognosis of gastric cancer. There are now two recognized types of macrophages, M1 and M2. It is widely accepted that M1 macrophages perform a fundamental function in inflammatory and immune response activation, whereas M2 macrophages are implicated in oncogenesis. Tumor-associated macrophages (TAM) also promote tumor angiogenesis and invasion by producing inflammatory factors, chemokines, and growth factors. For example, Wu et al. found that TAM-derived CXCL8 promotes tumor invasion and induces angiogenesis by stimulating tumor cells to secrete MMP-9 and VEGF (Wu et al., 2020). Wang et al. reported that tumor-associated fibroblasts promote immune escape by secreting IL-10 (Wang et al., 2015). In addition, This study discovered that M2 macrophages infiltrated the TME to a greater extent in high-risk patients and that cytokines such as IL-10, CXCL8, and CCL5 were expressed at a high level in the high-risk category as well, as determined by differential analysis. This further supports the accuracy of the findings in this study.

Chemotherapy and targeted therapy are widely used in gastric cancer. Immune checkpoint analysis shows that high-risk groups may have a higher sensitivity to immune checkpoint inhibitors. Twelve targeted pharmaceuticals, including AICAR, Axitinib, and ATRA, were shown to have increased sensitivity among high-risk patients. This study could offer a new approach to treating gastric cancer. The findings of this pharmacological screening, however, will need to be confirmed in larger clinical studies.

There are still some shortcomings in this study. First of all, data samples are mainly from TCGA data sets, which are limited and single. We will further investigate this in multi-center or multi-data sets. Second, the study had no underlying experimental validation. Therefore, basic experiments on LncRNAs associated with amino acid metabolism in gastric cancer will be further carried out in the future, mainly focusing on relevant mechanisms and signaling pathways.

## Conclusion

In summary, this study explored the prognostic and molecular immunological features of amino acid metabolism-related LncRNAs in gastric cancer. It was found that this prognostic model can offer an insightful perspective on the prognosis of patients with STAD, and provide innovative ideas for gastric cancer therapy in the future.

## Data Availability

The original contributions presented in the study are publicly available. This data can be found here: Supplementary Material.
