# Supplementary material for: Metabolism-related long non-coding RNA in the stomach cancer associated with 11 AMMLs predictive nomograms for OS in STAD
Source: Front Genet. 2023 Mar 13;14:1127132. doi: 10.3389/fgene.2023.1127132 (PMC10040790; doi:10.3389/fgene.2023.1127132)
Supplement: Supplementary file 1 [file Table1.DOCX]

AADAT

AANAT

AASS

ACAD8

ACADSB

ACAT1

ACMSD

ADI1

ADO

AFMID

AGMAT

AGXT

AGXT2

AHCY

AIMP1

AIMP2

ALDH18A1

ALDH4A1

ALDH6A1

ALDH7A1

ALDH9A1

AMD1

AMDHD1

AMT

APIP

ARG1

ARG2

ASL

ASMT

ASNS

ASPA

ASPG

ASRGL1

ASS1

AUH

AZIN1

AZIN2

BBOX1

BCAT1

BCAT2

BCKDHA

BCKDHB

BCKDK

BHMT

BHMT2

CARNMT1

CARNS1

CBS

CBSL

CDO1

CGA

CHDH

CKB

CKM

CKMT1A

CKMT1B

CKMT2

CPS1

CRYM

CSAD

CTH

DAO

DARS1

DBH

DBT

DCT

DDC

DDO

DHTKD1

DIO1

DIO2

DIO3

DLAT

DLD

DLST

DMGDH

DUOX1

DUOX2

ECHS1

EEF1E1

EEFSEC

ENOPH1

EPRS1

ETHE1

FAH

FAU

FOLH1

FTCD

GADL1

GAMT

GATM

GCAT

GCDH

GCSH

GLDC

GLS

GLS2

GLUD1

GLUD2

GLUL

GNMT

GOT1

GOT2

GPT

GPT2

GRHPR

GSR

GSTZ1

HAAO

HAL

HAO1

HDC

HGD

HIBADH

HIBCH

HNMT

HOGA1

HPD

HSD17B10

HYKK

IARS1

IDO1

IDO2

IL4I1

INMT

IVD

IYD

KARS1

KMO

KYAT1

KYAT3

KYNU

LARS1

LIAS

LIPT1

LIPT2

MARS1

MAT1A

MCCC1

MCCC2

MPST

MRI1

MTAP

MTR

MTRR

NAALAD2

NAGS

NAT8L

NDUFAB1

NMRAL1

NNMT

NQO1

OAT

OAZ1

OAZ2

OAZ3

OCA2

ODC1

OGDH

OTC

PAH

PAOX

PAPSS1

PAPSS2

PCBD1

PDHA1

PDHA2

PDHB

PDHX

PHGDH

PHYKPL

PIPOX

PNMT

PPM1K

PRODH

PRODH2

PSAT1

PSMA1

PSMA2

PSMA3

PSMA4

PSMA5

PSMA6

PSMA7

PSMA8

PSMB1

PSMB10

PSMB11

PSMB2

PSMB3

PSMB4

PSMB5

PSMB6

PSMB7

PSMB8

PSMB9

PSMC1

PSMC2

PSMC3

PSMC4

PSMC5

PSMC6

PSMD1

PSMD10

PSMD11

PSMD12

PSMD13

PSMD14

PSMD2

PSMD3

PSMD4

PSMD5

PSMD6

PSMD7

PSMD8

PSMD9

PSME1

PSME2

PSME3

PSME4

PSMF1

PSPH

PSTK

PXMP2

PYCR1

PYCR2

PYCR3

QARS1

QDPR

RARS1

RIDA

RIMKLA

RIMKLB

RPL10

RPL10A

RPL10L

RPL11

RPL12

RPL13

RPL13A

RPL14

RPL15

RPL17

RPL18

RPL18A

RPL19

RPL21

RPL22

RPL22L1

RPL23

RPL23A

RPL24

RPL26

RPL26L1

RPL27

RPL27A

RPL28

RPL29

RPL3

RPL30

RPL31

RPL32

RPL34

RPL35

RPL35A

RPL36

RPL36A

RPL36AL

RPL37

RPL37A

RPL38

RPL39

RPL39L

RPL3L

RPL4

RPL41

RPL5

RPL6

RPL7

RPL7A

RPL8

RPL9

RPLP0

RPLP1

RPLP2

RPS10

RPS11

RPS12

RPS13

RPS14

RPS15

RPS15A

RPS16

RPS17

RPS18

RPS19

RPS2

RPS20

RPS21

RPS23

RPS24

RPS25

RPS26

RPS27

RPS27A

RPS27L

RPS28

RPS29

RPS3

RPS3A

RPS4X

RPS4Y1

RPS4Y2

RPS5

RPS6

RPS7

RPS8

RPS9

RPSA

SARDH

SARS1

SAT1

SCLY

SDS

SDSL

SECISBP2

SEM1

SEPHS2

SEPSECS

SERINC1

SERINC2

SERINC3

SERINC4

SERINC5

SHMT1

SLC25A10

SLC25A12

SLC25A13

SLC25A15

SLC25A2

SLC25A21

SLC25A44

SLC36A4

SLC3A2

SLC44A1

SLC45A2

SLC5A5

SLC6A11

SLC6A12

SLC6A7

SLC6A8

SLC7A5

SMOX

SMS

SQOR

SRM

SRR

SUOX

TAT

TDO2

TH

TMLHE

TPH1

TPH2

TPO

TSHB

TST

TSTD1

TXN2

TXNRD1

TYR

TYRP1

UBA52

UROC1
