# Supplementary figures and images for: Metabolism-related long non-coding RNA in the stomach cancer associated with 11 AMMLs predictive nomograms for OS in STAD
Source: Front Genet. 2023 Mar 13;14:1127132. doi: 10.3389/fgene.2023.1127132 (PMC10040790; doi:10.3389/fgene.2023.1127132)

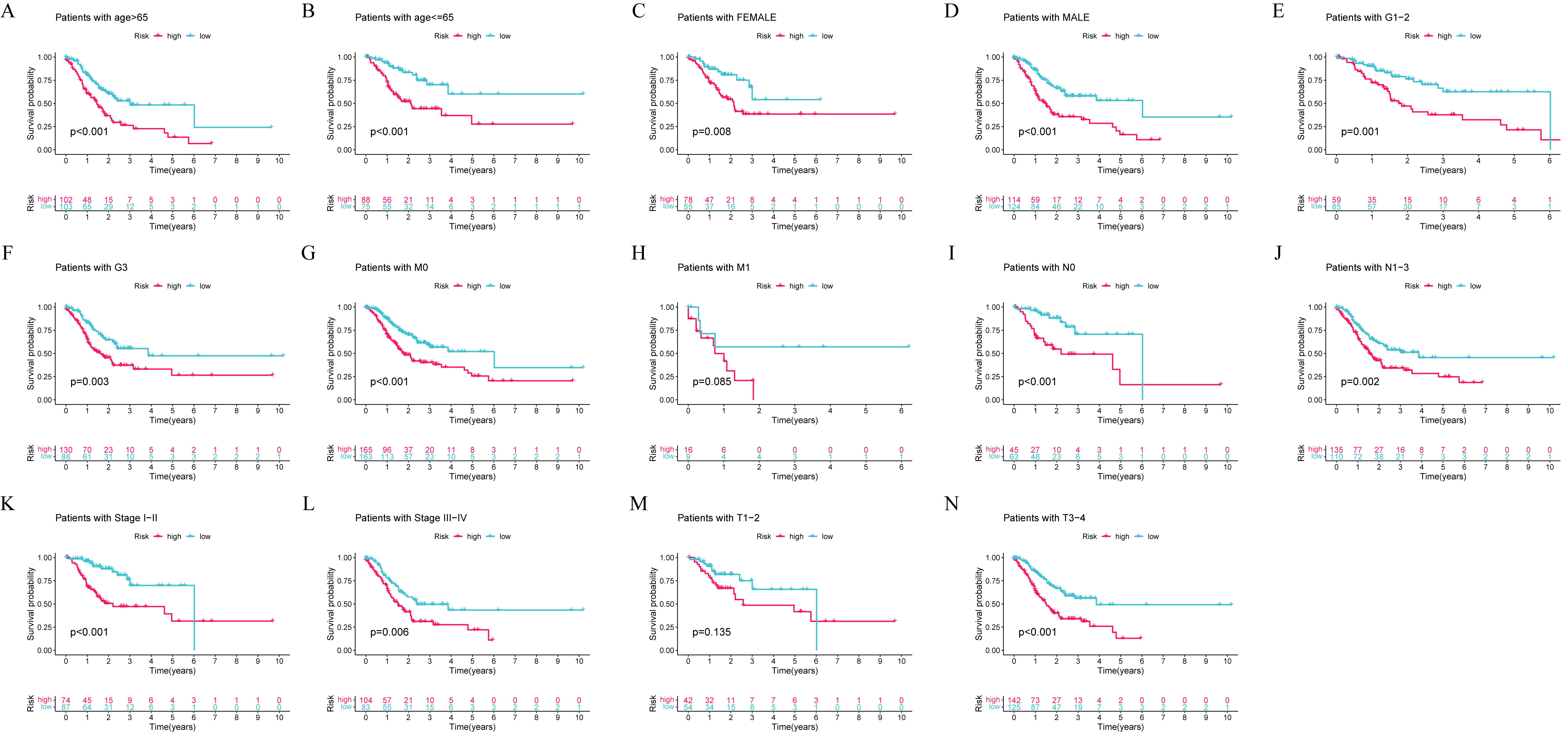

Supplement: Supplementary file 2 [file Image3.TIF]

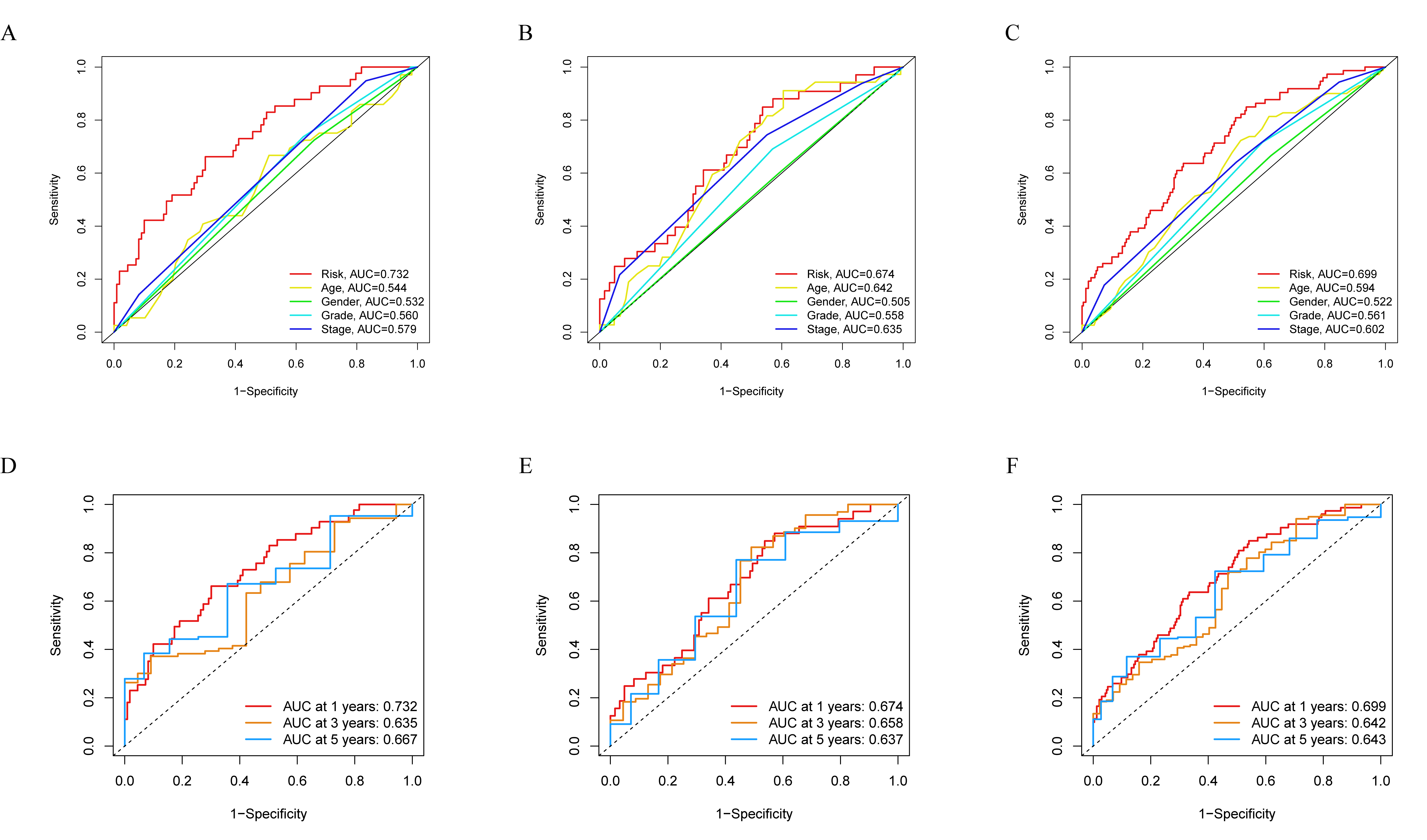

Supplement: Supplementary file 3 [file Image2.TIF]

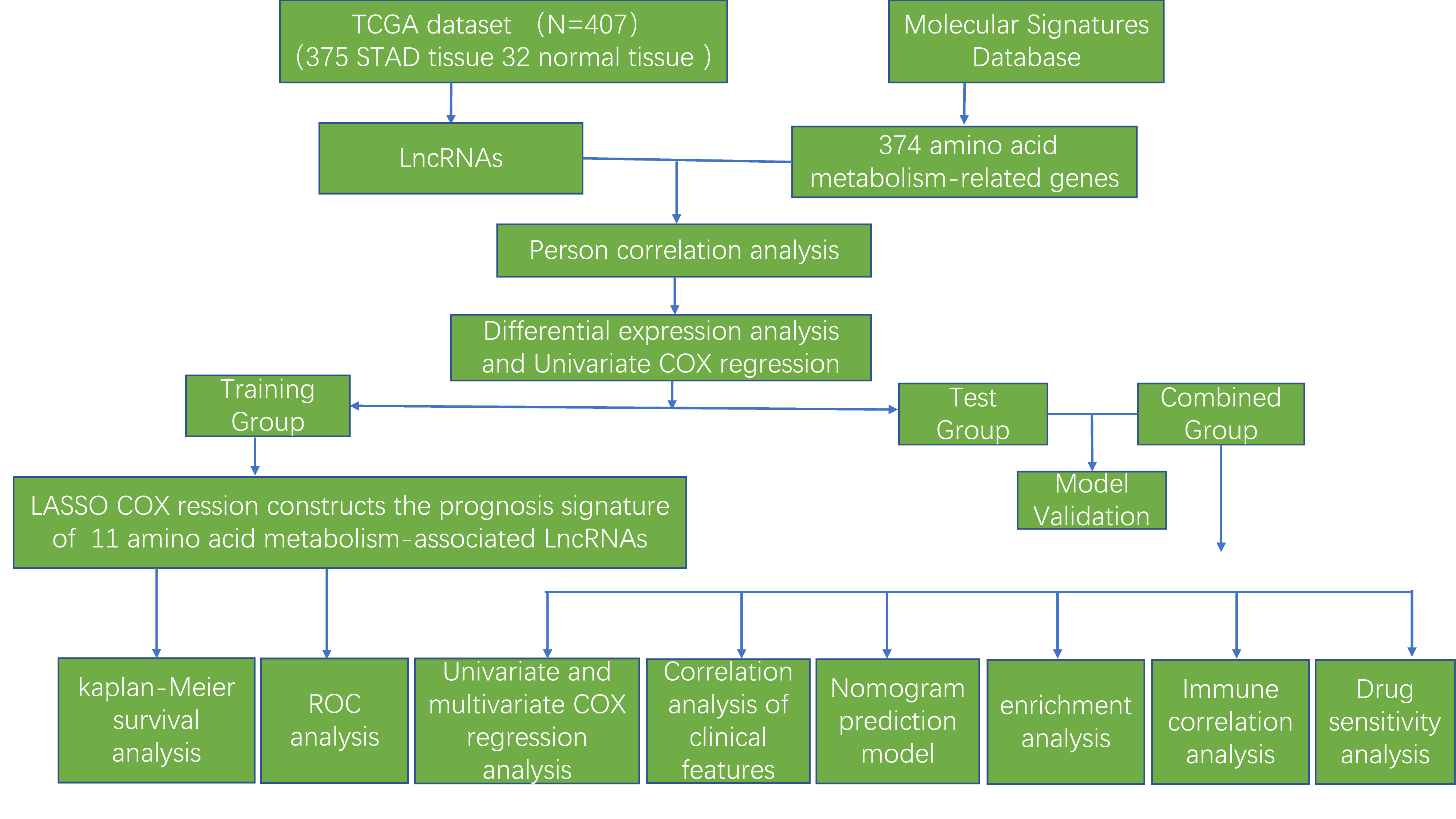

Supplement: Supplementary file 4 [file Image1.TIF]
