# Supplementary material for: Metabolism-related long non-coding RNA in the stomach cancer associated with 11 AMMLs predictive nomograms for OS in STAD
Source: Front Genet. 2023 Mar 13;14:1127132. doi: 10.3389/fgene.2023.1127132 (PMC10040790; doi:10.3389/fgene.2023.1127132)
Supplement: Supplementary file 5 [file Table2.DOCX]

m6A lncRNA cor pvalue Regulation

DMGDH CLIP1-AS1 0.435037613771518 9.52984582690063e-19 postive

RPL3L CLIP1-AS1 0.441927230175241 2.32578466030937e-19 postive

RPL3L ENO1-AS1 0.541420377058865 6.03457051265679e-30 postive

CSAD MUC20-OT1 0.461015032873821 3.93580370430099e-21 postive

PHYKPL MUC20-OT1 0.442487797701972 2.07069631165959e-19 postive

AFMID MIR200CHG 0.571913807043648 5.89716904832529e-34 postive

NAT8L MIR200CHG 0.493042842244661 2.29611477231355e-24 postive

BHMT2 FGF14-AS2 0.575293599444338 1.99562121885878e-34 postive

CKB FGF14-AS2 0.40926004250013 1.4141104412508e-16 postive

DMGDH FGF14-AS2 0.474561094884751 1.85612449990009e-22 postive

INMT FGF14-AS2 0.487474098905319 8.87194397946821e-24 postive

SLC25A12 FGF14-AS2 0.406501699145983 2.35481874516874e-16 postive

CSAD RAD51-AS1 0.656362837371602 1.42004955414042e-47 postive

LIPT1 RAD51-AS1 0.411680065445153 9.00490789983727e-17 postive

PHYKPL RAD51-AS1 0.572375467425647 5.08964763863107e-34 postive

PSMD8 RAD51-AS1 -0.427918864577538 3.95792156173112e-18 negative

SECISBP2 RAD51-AS1 0.417872039358398 2.79054556606072e-17 postive

BBOX1 SMILR 0.670686791979277 2.46547626020099e-50 postive

DUOX1 SMILR 0.526308705835007 4.17617387085091e-28 postive

SLC6A11 SMILR 0.420786291124986 1.59427190152522e-17 postive

PPM1K SLC16A1-AS1 0.465580660632956 1.42740926282108e-21 postive

BHMT2 LINC01778 0.688409480074154 5.68509498373883e-54 postive

DMGDH LINC01778 0.568649750425651 1.659434875655e-33 postive

INMT LINC01778 0.502651569196275 2.10120186277201e-25 postive

SLC25A12 LINC01778 0.556789375981823 6.47480912091272e-32 postive

DIO3 LINC02584 0.477972836176635 8.41629520550494e-23 postive

FTCD SNHG31 0.450962001669552 3.4828077314037e-20 postive

NAALAD2 SNHG31 0.559601889395974 2.75202211362238e-32 postive

RPL3L SNHG31 0.503575884157016 1.66273628989581e-25 postive

RPS24 NUTM2B-AS1 0.564261059883893 6.55028364229264e-33 postive

PHYKPL PSMD6-AS2 0.426403884006681 5.33573415459359e-18 postive

PSMB5 PSMD6-AS2 -0.436883626672797 6.55139323005986e-19 negative

PSMD8 PSMD6-AS2 -0.409413552700448 1.37434985930127e-16 negative

GLS LINC00242 0.470682620952526 4.51269898172393e-22 postive

NAALAD2 LINC00242 0.519893230149831 2.36838676369169e-27 postive

IL4I1 ETV7-AS1 0.419630647595943 1.99185067030233e-17 postive

PSMB9 ETV7-AS1 0.441465896563287 2.55871065758382e-19 postive

PSMA1 MIR222HG 0.418367764169466 2.53804298729982e-17 postive

CSAD LINC01089 0.497070430013571 8.50510702415881e-25 postive

PHYKPL LINC01089 0.483642899060465 2.21660966444449e-23 postive

HSD17B10 CHKB-DT 0.40639648489912 2.40084902684078e-16 postive

NMRAL1 CHKB-DT 0.404863510832617 3.18070789611351e-16 postive

SEM1 PPP1R35-AS1 0.457017312069867 9.4478903355296e-21 postive

CSAD PRMT5-AS1 0.432040826970189 1.74255319948986e-18 postive

RPL3L PRMT5-AS1 0.514775822914107 9.20989788179886e-27 postive

SCLY PRMT5-AS1 0.46192656533228 3.21823341039237e-21 postive

RPL3L CSE1L-AS1 0.67849711947253 6.61560050486314e-52 postive

DMGDH LINC01697 0.445283866800703 1.15627497587911e-19 postive

AASS CYP1B1-AS1 0.420980027013047 1.53573312622697e-17 postive

ASPA CYP1B1-AS1 0.550109427333931 4.78334132664298e-31 postive

AZIN2 CYP1B1-AS1 0.402239498403559 5.13060346577045e-16 postive

BHMT2 CYP1B1-AS1 0.714090400918614 9.8828909452567e-60 postive

CDO1 CYP1B1-AS1 0.517108970515203 4.97265351265786e-27 postive

DMGDH CYP1B1-AS1 0.767190045443492 6.22589037198377e-74 postive

ENOPH1 CYP1B1-AS1 -0.416607935667338 3.55153848587018e-17 negative

INMT CYP1B1-AS1 0.712781061692129 2.01306410984297e-59 postive

MTR CYP1B1-AS1 0.451970094346486 2.80791809480941e-20 postive

PPM1K CYP1B1-AS1 0.468548165078634 7.32290940559542e-22 postive

PSMD14 CYP1B1-AS1 -0.439401158394309 3.9153408404327e-19 negative

SLC25A10 CYP1B1-AS1 -0.408764045041823 1.55045621363316e-16 negative

AFMID CAMTA1-DT 0.465310180481456 1.51645189056586e-21 postive

NAT8L CAMTA1-DT 0.454045038970354 1.79829752015921e-20 postive

RPL3L CAMTA1-DT 0.464249915051321 1.92141037594534e-21 postive

AASS ADAMTS9-AS2 0.467968017805278 8.34785183698838e-22 postive

ASPA ADAMTS9-AS2 0.52908684671334 1.94714055300487e-28 postive

BHMT2 ADAMTS9-AS2 0.841088395009615 1.38809034846439e-101 postive

CDO1 ADAMTS9-AS2 0.432164495550222 1.69989204429009e-18 postive

CKB ADAMTS9-AS2 0.406414991804402 2.39268889021854e-16 postive

DMGDH ADAMTS9-AS2 0.762875514964796 1.21884544111091e-72 postive

ENOPH1 ADAMTS9-AS2 -0.437310488366877 6.0056225853528e-19 negative

INMT ADAMTS9-AS2 0.699988548706797 1.7135242133895e-56 postive

MTR ADAMTS9-AS2 0.460871316193439 4.06248672038123e-21 postive

PPM1K ADAMTS9-AS2 0.514062750859508 1.11083552840957e-26 postive

PSMA5 ADAMTS9-AS2 -0.444143704063502 1.46734557838102e-19 negative

PSMD14 ADAMTS9-AS2 -0.468482709238308 7.43203756032785e-22 negative

SLC25A10 ADAMTS9-AS2 -0.425561729018399 6.29543328139978e-18 negative

SLC25A12 ADAMTS9-AS2 0.60937675067525 1.71205770434721e-39 postive

CSAD ZFHX2-AS1 0.514456914674682 1.00156907650697e-26 postive

LIPT1 ZFHX2-AS1 0.417545305027774 2.97030119903808e-17 postive

PHYKPL ZFHX2-AS1 0.42192564048502 1.27898725170473e-17 postive

CSAD LINC00106 0.542087062198108 4.98089834200274e-30 postive

PHYKPL LINC00106 0.450759695828002 3.6363434436381e-20 postive

AANAT GHRLOS 0.433062616324408 1.41942391775071e-18 postive

CSAD GHRLOS 0.422972735000022 1.04375360245626e-17 postive

PHYKPL GHRLOS 0.454422007128085 1.65790979594396e-20 postive

PPM1K GHRLOS 0.453483550121897 2.02936522988884e-20 postive

SCLY GHRLOS 0.411879610661399 8.67454200321333e-17 postive

AMDHD1 TMEM44-AS1 0.400864964743055 6.57973672791474e-16 postive

MCCC1 TMEM44-AS1 0.434357477160823 1.09345095710529e-18 postive

SEM1 TMEM44-AS1 0.428553066591352 3.49113554374651e-18 postive

LIPT1 LACTB2-AS1 0.401530290937122 5.83412257158711e-16 postive

PSMD1 LACTB2-AS1 0.457699622743649 8.14292329117955e-21 postive

CKM LINC02057 0.497639446871442 7.38383214052636e-25 postive

SLC5A5 LINC02057 0.499410160401813 4.74767851367969e-25 postive

OAZ3 DNAJC27-AS1 0.446553808254254 8.85835620343822e-20 postive

RPL3L DNAJC27-AS1 0.597956243355716 9.97455976656739e-38 postive

SLC7A5 MIR31HG 0.405144429807319 3.02123932944817e-16 postive

RPL3L DENND5B-AS1 0.431576582561877 1.91230318773518e-18 postive

CPS1 LINC02672 0.572166306558799 5.44096854892136e-34 postive

LIPT1 CCDC28A-AS1 0.420324903294184 1.74265754585349e-17 postive

RPL3L CCDC28A-AS1 0.516877274808708 5.2876363926458e-27 postive

DIO3 LINC02753 0.486066858206905 1.24356750192755e-23 postive

DMGDH LINC02289 0.423876911320998 8.7524499540666e-18 postive

RPL3L LINC02289 0.494527556718298 1.59463122847745e-24 postive

CKB ACVR2B-AS1 0.567075668954094 2.721820385547e-33 postive

RPL3L ACVR2B-AS1 0.467955536821865 8.37138789493999e-22 postive

ASPA RBMS3-AS3 0.485165173298836 1.54269275065883e-23 postive

BHMT2 RBMS3-AS3 0.710027853410298 8.87009294397195e-59 postive

DMGDH RBMS3-AS3 0.607356005169633 3.55713329424098e-39 postive

INMT RBMS3-AS3 0.633087860099599 2.13661033604904e-43 postive

PPM1K RBMS3-AS3 0.421200313954585 1.47173563171332e-17 postive

PSMD14 RBMS3-AS3 -0.406364238794197 2.41513242851683e-16 negative

SLC25A10 RBMS3-AS3 -0.41868463911522 2.38854753351249e-17 negative

SLC25A12 RBMS3-AS3 0.450916271323089 3.51694556037924e-20 postive

SLC7A5 TM4SF19-AS1 0.410818586055695 1.05787664259081e-16 postive

GSR TNFRSF10A-AS1 0.501482911331798 2.82188735967214e-25 postive

AFMID DOCK9-DT 0.58054414305395 3.61521923703382e-35 postive

NAT8L DOCK9-DT 0.620593912534922 2.68131087438255e-41 postive

NAALAD2 LINC00865 0.44520769989153 1.17485836890824e-19 postive

ASPA PGM5P4-AS1 0.488873802444434 6.33110350453606e-24 postive

BHMT2 PGM5P4-AS1 0.47100330134021 4.19489662459253e-22 postive

CDO1 PGM5P4-AS1 0.48679012644483 1.04563772016678e-23 postive

DMGDH PGM5P4-AS1 0.583548336444164 1.34135306694958e-35 postive

INMT PGM5P4-AS1 0.55594892045739 8.3479462168405e-32 postive

PSMD14 SNHG3 0.431819600345423 1.82151044526464e-18 postive

SCLY SNHG3 0.444912890562074 1.24959835726933e-19 postive

PHYKPL ACAP2-IT1 0.438275247774558 4.93155372226901e-19 postive

PSME4 ACAP2-IT1 0.421036898088822 1.51895381122481e-17 postive

SECISBP2 ACAP2-IT1 0.436211780843198 7.51071892940575e-19 postive

SEPSECS ACAP2-IT1 0.458820668568501 6.37384199718271e-21 postive

PIPOX LBX2-AS1 0.429716863815281 2.77112617357231e-18 postive

SEPHS2 LBX2-AS1 0.46760648593542 9.05676048784665e-22 postive

AFMID LINC02268 0.578830864733521 6.33444976814437e-35 postive

NAT8L LINC02268 0.70273022970274 4.16046236131503e-57 postive

AANAT LINC00861 0.4327077362926 1.52434327832031e-18 postive

KMO LINC00861 0.676680834444954 1.54994073082511e-51 postive

PHYKPL LINC00861 0.423562974178015 9.30478031969295e-18 postive

PPM1K LINC00861 0.646091999465726 1.09810788664546e-45 postive

AFMID MELTF-AS1 0.437983436368219 5.23475403788048e-19 postive

NAALAD2 HCG15 0.488184266847398 7.47744640018966e-24 postive

RPL3L HCG15 0.400452709832654 7.08785438852105e-16 postive

KMO LINC01215 0.652537260244451 7.31539920171985e-47 postive

PPM1K LINC01215 0.682954157660612 7.97689876704001e-53 postive

CSAD ZKSCAN2-DT 0.48797307921638 7.86785352944129e-24 postive

PHYKPL ZKSCAN2-DT 0.471287661219074 3.93160860787794e-22 postive

SCLY ZKSCAN2-DT 0.401459412410623 5.90943021957619e-16 postive

FTCD LINC00235 0.414852627135479 4.95573033729966e-17 postive

AFMID STX18-AS1 0.493773223119348 1.91955572432726e-24 postive

LIAS STX18-AS1 0.482323689320106 3.03016330219268e-23 postive

NAT8L STX18-AS1 0.523512943902359 8.93696018761018e-28 postive

SEPSECS STX18-AS1 0.471654647659914 3.61576803428297e-22 postive

RPL3L LINC00562 0.46453390148587 1.80352883476815e-21 postive

KYAT1 LINC00460 0.4310013741793 2.14531355913918e-18 postive

TH LINC00460 0.47288990828897 2.72558049431936e-22 postive

KMO MIR155HG 0.597637622793282 1.11463908573893e-37 postive

PPM1K MIR155HG 0.506961955447637 7.01149149755192e-26 postive

PSMB9 MIR155HG 0.400011112882104 7.67486313648476e-16 postive

PHYKPL ERVK13-1 0.490891610214962 3.88185080169719e-24 postive

HAAO KCTD21-AS1 0.403771956293341 3.8826124983343e-16 postive

CKMT1A DSG2-AS1 0.41671893384693 3.47726932453023e-17 postive

CKMT1B DSG2-AS1 0.429860781057276 2.69292837161926e-18 postive

AANAT ZBTB20-AS1 0.477254148529992 9.94940460508656e-23 postive

DMGDH ZBTB20-AS1 0.47471921430287 1.78966985346065e-22 postive

KMO ZBTB20-AS1 0.414043825470652 5.77421003806118e-17 postive

PHYKPL ZBTB20-AS1 0.416648203590186 3.52441671456981e-17 postive

PPM1K ZBTB20-AS1 0.560640362044415 2.00243007396627e-32 postive

CSAD FBXO36-IT1 0.401645890623454 5.71331510428462e-16 postive

RPL3L FBXO36-IT1 0.538329722450777 1.46074318345804e-29 postive

PHYKPL N4BP2L2-IT2 0.458799543415286 6.40338397110655e-21 postive

SECISBP2 N4BP2L2-IT2 0.441701993845344 2.43677422438678e-19 postive

CSAD HCG27 0.47388905977061 2.1667732305419e-22 postive

KMO HCG27 0.445791623893757 1.0395756021094e-19 postive

PHYKPL HCG27 0.480852990762608 4.28689184251893e-23 postive

PHYKPL SPAG5-AS1 0.405627561280627 2.76514026557703e-16 postive

AASS MAGI2-AS3 0.520411937839826 2.0611515370897e-27 postive

AHCY MAGI2-AS3 -0.417190337530317 3.17849165096797e-17 negative

AIMP2 MAGI2-AS3 -0.409118664923182 1.45172645637862e-16 negative

ASPA MAGI2-AS3 0.59099081747528 1.09981221154101e-36 postive

BHMT2 MAGI2-AS3 0.825436545698199 1.16886304155153e-94 postive

CDO1 MAGI2-AS3 0.600704425919478 3.80678354044103e-38 postive

DIO2 MAGI2-AS3 0.498869163876081 5.43500203160493e-25 postive

DMGDH MAGI2-AS3 0.725848981042594 1.38052569274033e-62 postive

ECHS1 MAGI2-AS3 -0.428865331791878 3.28163185493982e-18 negative

ENOPH1 MAGI2-AS3 -0.457827689446193 7.91858510647307e-21 negative

GOT1 MAGI2-AS3 -0.410718784921321 1.07777042006576e-16 negative

INMT MAGI2-AS3 0.737804891754733 1.20382513726702e-65 postive

MTR MAGI2-AS3 0.516409799211912 5.98434106946768e-27 postive

PPM1K MAGI2-AS3 0.523607726883552 8.7104023457357e-28 postive

PSMA5 MAGI2-AS3 -0.448830895992336 5.4785279845127e-20 negative

PSMB2 MAGI2-AS3 -0.441599534426004 2.48897231989527e-19 negative

PSMB5 MAGI2-AS3 -0.41565806889402 4.25422003567341e-17 negative

PSMD13 MAGI2-AS3 -0.400954839468701 6.47380385869477e-16 negative

PSMD14 MAGI2-AS3 -0.491827235758763 3.09068201018224e-24 negative

PYCR1 MAGI2-AS3 -0.431947120313355 1.77557811505166e-18 negative

SERINC1 MAGI2-AS3 0.56975901598628 1.16902770364565e-33 postive

SLC25A10 MAGI2-AS3 -0.537168246528852 2.03157487786957e-29 negative

SLC25A12 MAGI2-AS3 0.550840728041387 3.85123805092369e-31 postive

SLC3A2 MAGI2-AS3 -0.400307533330015 7.27580116612552e-16 negative

TXN2 MAGI2-AS3 -0.409295968560681 1.40470520958422e-16 negative

FTCD RAMP2-AS1 0.415010890707219 4.80946404799146e-17 postive

NAALAD2 RAMP2-AS1 0.540652770656087 7.52264712248173e-30 postive

IYD VIPR1-AS1 0.415917751209623 4.0495809263859e-17 postive

PHYKPL VIPR1-AS1 0.404783508902489 3.22761462885058e-16 postive

SECISBP2 VIPR1-AS1 0.427248152835238 4.51835564940558e-18 postive

CSAD SAP30L-AS1 0.413205235357168 6.76290282919056e-17 postive

PHYKPL SAP30L-AS1 0.441484544152955 2.54886528501621e-19 postive

BHMT2 LINC02762 0.512785484672129 1.55226940284e-26 postive

DMGDH LINC02762 0.443731511468826 1.5989825386375e-19 postive

INMT LINC02762 0.4638440307936 2.10317507172703e-21 postive

SLC25A12 LINC02762 0.43643913408775 7.17154803727694e-19 postive

PSMA4 TFAP2A-AS1 0.410395433209526 1.14478242360928e-16 postive

PSME2 TFAP2A-AS1 0.415886172533216 4.07393993698357e-17 postive

RPL10A GAS5 0.520801154556991 1.85679146016375e-27 postive

RPL11 GAS5 0.517726449664285 4.22087540967166e-27 postive

RPL12 GAS5 0.579399791290101 5.25998843766345e-35 postive

RPL13A GAS5 0.428995457158812 3.19802361271163e-18 postive

RPL14 GAS5 0.446371434309626 9.20448658025229e-20 postive

RPL15 GAS5 0.403015139140068 4.45636653267825e-16 postive

RPL22 GAS5 0.473043732604436 2.63111224610172e-22 postive

RPL24 GAS5 0.435263912631588 9.10306946555792e-19 postive

RPL26 GAS5 0.483426486564146 2.33347619858634e-23 postive

RPL27A GAS5 0.446287414291044 9.36839603616658e-20 postive

RPL29 GAS5 0.434309970556211 1.10399012284559e-18 postive

RPL3 GAS5 0.413153775633999 6.82871487635521e-17 postive

RPL30 GAS5 0.450819960410541 3.58992306806699e-20 postive

RPL31 GAS5 0.591255607417818 1.00496924124004e-36 postive

RPL34 GAS5 0.478594194082882 7.28029068221406e-23 postive

RPL36 GAS5 0.483011916445841 2.57457268353667e-23 postive

RPL36A GAS5 0.416116790447299 3.89930710349203e-17 postive

RPL37A GAS5 0.496639921159149 9.46359515856383e-25 postive

RPL5 GAS5 0.50640280168389 8.09145320366641e-26 postive

RPL6 GAS5 0.479898973391585 5.36408099421481e-23 postive

RPL7 GAS5 0.475252421003714 1.58240117998017e-22 postive

RPL7A GAS5 0.57474270423323 2.38314796134767e-34 postive

RPLP0 GAS5 0.508404369503011 4.83932345852888e-26 postive

RPS12 GAS5 0.530198076276794 1.43215102857592e-28 postive

RPS13 GAS5 0.497283193108079 8.06743933007288e-25 postive

RPS15A GAS5 0.413875003000996 5.96110229387912e-17 postive

RPS18 GAS5 0.528714553626233 2.15765875893095e-28 postive

RPS23 GAS5 0.413308586304794 6.63260338599147e-17 postive

RPS24 GAS5 0.429459582682407 2.9165224863243e-18 postive

RPS27A GAS5 0.55508957306724 1.08165652881302e-31 postive

RPS3 GAS5 0.45375101067352 1.9158654836836e-20 postive

RPS3A GAS5 0.454088016831629 1.78171877633107e-20 postive

RPS7 GAS5 0.508467953254476 4.76067736973675e-26 postive

RPS8 GAS5 0.548913012836256 6.81133253616894e-31 postive

AFMID LINC01719 0.43211738833107 1.71601981814232e-18 postive

NAT8L LINC01719 0.514454731512828 1.00214401781574e-26 postive

PSMD4 LINC02633 0.475309834833673 1.56154686756629e-22 postive

FTCD LINC02575 0.465932083420793 1.31937729524087e-21 postive

NAT8L LINC02575 0.425732622391618 6.0878727089308e-18 postive

DMGDH C1QTNF7-AS1 0.426089879998752 5.6754538800934e-18 postive

CSAD IGBP1-AS1 0.468764731772708 6.97298351440927e-22 postive

PHYKPL IGBP1-AS1 0.431005174001468 2.14368626928422e-18 postive

RPL3L LINC02166 0.411141700075039 9.95910850050609e-17 postive

RPL3L LARS2-AS1 0.417219605063812 3.1607971691611e-17 postive

ASPA BARX1-DT 0.424777146816102 7.34117763402449e-18 postive

BHMT2 BARX1-DT 0.809084411788717 3.98275051510123e-88 postive

CKB BARX1-DT 0.457699001619298 8.14402644295221e-21 postive

DMGDH BARX1-DT 0.624018049471776 7.28850755401962e-42 postive

INMT BARX1-DT 0.647358532916047 6.48216778706407e-46 postive

PPM1K BARX1-DT 0.430234015091714 2.5000999259749e-18 postive

SLC25A12 BARX1-DT 0.617938222895841 7.28299608365952e-41 postive

RPL3L NSMCE1-DT 0.49470739522386 1.52556013119277e-24 postive

PSMA7 HAR1A 0.439239174068037 4.04771879253809e-19 postive

RPS21 HAR1A 0.459472235110951 5.52575855372004e-21 postive

PSMD4 LY6E-DT 0.402967161066405 4.49542093227485e-16 postive

BBOX1 CERS3-AS1 0.47052007807919 4.68272879201765e-22 postive

DUOX1 CERS3-AS1 0.455495551926048 1.31458898879512e-20 postive

GRHPR MYOSLID 0.524836037539988 6.24029088967451e-28 postive

PSMC1 MYOSLID 0.451240146733401 3.2820885764322e-20 postive

ASPA LRRK2-DT 0.446750805986711 8.49885954832443e-20 postive

BHMT2 LRRK2-DT 0.503564178200366 1.66767947594415e-25 postive

DMGDH LRRK2-DT 0.640338314975814 1.16703117904112e-44 postive

INMT LRRK2-DT 0.473569244360561 2.33208458850215e-22 postive

KMO LRRK2-DT 0.458478724510255 6.86895818744711e-21 postive

PPM1K LRRK2-DT 0.58646758139918 5.06781930487753e-36 postive

DHTKD1 WAC-AS1 0.496279464037935 1.03475072400893e-24 postive

FTCD CASC20 0.479165153562669 6.37045946575125e-23 postive

NAALAD2 CASC20 0.579227655451576 5.564492500885e-35 postive

RPL3L CASC20 0.503344083871997 1.76336781016176e-25 postive

ASPG LINC02560 0.469970173167618 5.30612629785143e-22 postive

BBOX1 LINC02560 0.745514741736932 1.04048226763882e-67 postive

DUOX1 LINC02560 0.742514922495801 6.74264588831386e-67 postive

SLC6A11 LINC02560 0.572638163599047 4.68004603225064e-34 postive

ASPA LINC00092 0.516052198544963 6.57779849289662e-27 postive

BHMT2 LINC00092 0.499345629613417 4.82492766886928e-25 postive

DMGDH LINC00092 0.507403051563169 6.26108162831375e-26 postive

INMT LINC00092 0.574092041997562 2.93761218125699e-34 postive

PPM1K LINC00092 0.565396846431275 4.60036149501715e-33 postive

PSMD14 LINC00092 -0.450931921325436 3.50522587293365e-20 negative

CDO1 WDFY3-AS2 0.439056116534316 4.20262312451619e-19 postive

DMGDH WDFY3-AS2 0.534589589495988 4.20641240834211e-29 postive

NDUFAB1 PPP1R14B-AS1 0.410458571493518 1.13138382512365e-16 postive

SLC3A2 FRGCA 0.474594889945566 1.84171970585437e-22 postive

SMOX FRGCA 0.460306452494078 4.60050462011635e-21 postive

CSAD MPRIP-AS1 0.535881365507897 2.92356142995556e-29 postive

GCSH VPS9D1-AS1 0.519632028254625 2.53980132182943e-27 postive

PSMB5 VPS9D1-AS1 0.425477316158813 6.40050917202196e-18 postive

PYCR3 VPS9D1-AS1 0.503465666414733 1.70985775081951e-25 postive

SLC25A10 VPS9D1-AS1 0.442414300250755 2.10250368484118e-19 postive

SRM VPS9D1-AS1 0.520844386795702 1.83536576735344e-27 postive

AFMID DPP10-AS1 0.551483230585076 3.18205574299448e-31 postive

NAT8L DPP10-AS1 0.64823416851778 4.49585684444702e-46 postive

GLS KCNQ1OT1 0.514841416515727 9.0522777395169e-27 postive

RPL3L LINC00862 0.556603153478585 6.85023088980555e-32 postive

CGA CYP4F26P 0.425814965761405 5.99027803969686e-18 postive

GRHPR LINC02542 0.56132861223693 1.62093907640641e-32 postive

PSMC1 LINC02542 0.466583370104948 1.14004023436086e-21 postive

BHMT2 LYPLAL1-DT 0.543819056076476 3.01945861316886e-30 postive

CDO1 LYPLAL1-DT 0.444243048910769 1.43724962983204e-19 postive

DMGDH LYPLAL1-DT 0.486818932216699 1.03843428300627e-23 postive

INMT LYPLAL1-DT 0.425544944314491 6.31619066222311e-18 postive

SLC25A12 LYPLAL1-DT 0.467413563212491 9.45899616562417e-22 postive

FTCD CAPN10-DT 0.407698098643115 1.88863222663853e-16 postive

NAALAD2 CAPN10-DT 0.602049369945594 2.36781809281846e-38 postive

SCLY CAPN10-DT 0.447782005052346 6.83890698203053e-20 postive

PSMD1 INTS9-AS1 0.413281472873244 6.6665460131047e-17 postive

FTCD H19 0.438640120465453 4.576656354699e-19 postive

RPL3L NQO2-AS1 0.472755619244585 2.81077945242773e-22 postive

PSMD1 LINC01655 0.402708521471461 4.71181239986889e-16 postive

BHMT2 FLRT1 0.497725189439798 7.22803194138351e-25 postive

SLC25A12 FLRT1 0.437172225548122 6.17731302513373e-19 postive

TPO FLRT1 0.61202214707896 6.52079188411613e-40 postive

PHYKPL NFYC-AS1 0.511783584232387 2.01616989167323e-26 postive

GLDC PROX1-AS1 0.477285575751185 9.87694534655404e-23 postive

KYAT1 PROX1-AS1 0.405816102399153 2.67108779441719e-16 postive

SMOX PROX1-AS1 0.426017142324331 5.75713217748933e-18 postive

RPL3L LINC02816 0.477625584498353 9.12552054105109e-23 postive

GLS TMEM147-AS1 0.45412843514592 1.76626475228592e-20 postive

SCLY TMEM147-AS1 0.409583456349021 1.33162251310058e-16 postive

PPM1K KDM2B-DT 0.411652798428738 9.05100003019868e-17 postive

RPL3L KDM2B-DT 0.435227129089427 9.17113690686088e-19 postive

INMT LINC00957 0.455393519022085 1.34394927763531e-20 postive

PPM1K LINC00957 0.437530484720099 5.74206837864735e-19 postive

PSMA5 LINC00957 -0.401172947489933 6.22363601688078e-16 negative

TPO LINC00957 0.476114336573065 1.29630602936081e-22 postive

FTCD DSCR4-IT1 0.584090303004327 1.12043336045521e-35 postive

RPL3L DSCR4-IT1 0.411778635790385 8.84019963647773e-17 postive

NAT8L WARS2-AS1 0.405171922712418 3.00606081041444e-16 postive

CSAD ZDHHC20-IT1 0.676321979494066 1.83254196082819e-51 postive

RPL3L KTN1-AS1 0.513786755909305 1.1942692985513e-26 postive

CSAD ADIRF-AS1 0.406850582285208 2.20829946426075e-16 postive

FAU EPB41L4A-AS1 0.413882065879615 5.9531656901368e-17 postive

RPL10 EPB41L4A-AS1 0.499432624770937 4.7210740786491e-25 postive

RPL10A EPB41L4A-AS1 0.605888115370074 6.03074363987541e-39 postive

RPL11 EPB41L4A-AS1 0.540265010489125 8.40684312805433e-30 postive

RPL12 EPB41L4A-AS1 0.623070875314745 1.04670653653628e-41 postive

RPL13 EPB41L4A-AS1 0.474137828802566 2.04622221600753e-22 postive

RPL13A EPB41L4A-AS1 0.596450047283408 1.68447349896699e-37 postive

RPL14 EPB41L4A-AS1 0.570159954565191 1.02966111801651e-33 postive

RPL15 EPB41L4A-AS1 0.580612300155234 3.53520884404263e-35 postive

RPL18 EPB41L4A-AS1 0.486210055158855 1.20164884937262e-23 postive

RPL18A EPB41L4A-AS1 0.448242904357271 6.20443077582391e-20 postive

RPL23A EPB41L4A-AS1 0.485487954322423 1.42824001771787e-23 postive

RPL24 EPB41L4A-AS1 0.490471333873233 4.29935179517057e-24 postive

RPL26 EPB41L4A-AS1 0.525675566733173 4.96447750071311e-28 postive

RPL27 EPB41L4A-AS1 0.435067369529641 9.47262532792878e-19 postive

RPL27A EPB41L4A-AS1 0.527736326952779 2.82404512976318e-28 postive

RPL29 EPB41L4A-AS1 0.604515070890735 9.85715038358736e-39 postive

RPL3 EPB41L4A-AS1 0.609250893938762 1.79211053213229e-39 postive

RPL30 EPB41L4A-AS1 0.400582818157999 6.92346858615369e-16 postive

RPL31 EPB41L4A-AS1 0.58783261701602 3.20394213383663e-36 postive

RPL32 EPB41L4A-AS1 0.583730663638841 1.26260069460845e-35 postive

RPL34 EPB41L4A-AS1 0.552655201922178 2.24410373373777e-31 postive

RPL35 EPB41L4A-AS1 0.448397276400186 6.0051638691614e-20 postive

RPL35A EPB41L4A-AS1 0.520637804337204 1.94000044134725e-27 postive

RPL36 EPB41L4A-AS1 0.595937884598554 2.01174667890012e-37 postive

RPL36A EPB41L4A-AS1 0.459324554577129 5.70766223032323e-21 postive

RPL37A EPB41L4A-AS1 0.568454520595257 1.76472233844475e-33 postive

RPL38 EPB41L4A-AS1 0.415436156597753 4.43711925478446e-17 postive

RPL4 EPB41L4A-AS1 0.460646493689303 4.26876256533932e-21 postive

RPL5 EPB41L4A-AS1 0.52960594220946 1.68709188050878e-28 postive

RPL6 EPB41L4A-AS1 0.557419547305277 5.34907832604009e-32 postive

RPL7 EPB41L4A-AS1 0.431958368639222 1.77158160339562e-18 postive

RPL7A EPB41L4A-AS1 0.568028551837511 2.01793069378391e-33 postive

RPL8 EPB41L4A-AS1 0.407684240628021 1.8934741415875e-16 postive

RPL9 EPB41L4A-AS1 0.542477324536255 4.45078618952073e-30 postive

RPLP0 EPB41L4A-AS1 0.587015107576717 4.21754017706687e-36 postive

RPLP2 EPB41L4A-AS1 0.472922699472961 2.70516605661808e-22 postive

RPS11 EPB41L4A-AS1 0.500209112004113 3.88663377261334e-25 postive

RPS12 EPB41L4A-AS1 0.493342473243301 2.13354837980274e-24 postive

RPS13 EPB41L4A-AS1 0.520739246461386 1.88790361644831e-27 postive

RPS14 EPB41L4A-AS1 0.538640560314825 1.33702673299934e-29 postive

RPS15 EPB41L4A-AS1 0.497676946018811 7.31529002175313e-25 postive

RPS16 EPB41L4A-AS1 0.408073945095905 1.76185160391122e-16 postive

RPS17 EPB41L4A-AS1 0.450503028789448 3.84075562921323e-20 postive

RPS18 EPB41L4A-AS1 0.50448290062335 1.32062606545737e-25 postive

RPS19 EPB41L4A-AS1 0.445967208524573 1.00198253749568e-19 postive

RPS2 EPB41L4A-AS1 0.464497924260352 1.8180591632256e-21 postive

RPS20 EPB41L4A-AS1 0.416922198871919 3.34519909219927e-17 postive

RPS23 EPB41L4A-AS1 0.606169362949086 5.45173749274507e-39 postive

RPS24 EPB41L4A-AS1 0.43818084501694 5.02770004207553e-19 postive

RPS25 EPB41L4A-AS1 0.496479439329914 9.84752709670151e-25 postive

RPS27 EPB41L4A-AS1 0.411605701213079 9.13115945321706e-17 postive

RPS27A EPB41L4A-AS1 0.513409350349641 1.31845456074608e-26 postive

RPS28 EPB41L4A-AS1 0.552945431070775 2.05774592508193e-31 postive

RPS29 EPB41L4A-AS1 0.449840621891878 4.42203930927367e-20 postive

RPS3 EPB41L4A-AS1 0.458886758776135 6.28228396233411e-21 postive

RPS3A EPB41L4A-AS1 0.481705810580208 3.50637067721082e-23 postive

RPS4X EPB41L4A-AS1 0.455146555510152 1.41771345932165e-20 postive

RPS5 EPB41L4A-AS1 0.516960854467265 5.17180382332928e-27 postive

RPS6 EPB41L4A-AS1 0.48527996218441 1.50099006883519e-23 postive

RPS7 EPB41L4A-AS1 0.459673224455031 5.28733682065093e-21 postive

RPS8 EPB41L4A-AS1 0.605145556045072 7.86861680895154e-39 postive

RPS9 EPB41L4A-AS1 0.592188815805316 7.30854607589126e-37 postive

RPSA EPB41L4A-AS1 0.440743871773933 2.9700911667778e-19 postive

UBA52 EPB41L4A-AS1 0.435285936060855 9.0625535040017e-19 postive

KMO LINC00528 0.615462330733134 1.8330604623445e-40 postive

PPM1K LINC00528 0.535124334235584 3.61900127211653e-29 postive

CSAD TMED2-DT 0.533406492017248 5.86169242907263e-29 postive

LIPT1 TMED2-DT 0.434005382172484 1.17397577641794e-18 postive

RPL3L TMED2-DT 0.561197541671788 1.68757797664032e-32 postive

SCLY TMED2-DT 0.485717453799256 1.35199889945426e-23 postive

PHYKPL ANKRD10-IT1 0.410210037114448 1.18503239287995e-16 postive

CSAD ZNF630-AS1 0.403627552219729 3.98617882038705e-16 postive

BCKDK FOXN3-AS1 0.449857147926602 4.40653505514197e-20 postive

OAZ2 FOXN3-AS1 0.5022631487198 2.31786985615707e-25 postive

AFMID MINCR 0.655272810491053 2.27097091823901e-47 postive

NAT8L MINCR 0.64739967122805 6.37186112504709e-46 postive

PYCR1 MINCR 0.478697941085966 7.10594486054996e-23 postive

RIDA MINCR 0.404875793718339 3.17356570207042e-16 postive

CSAD GTF3C2-AS1 0.490008298429316 4.81071741777495e-24 postive

RPL3L GTF3C2-AS1 0.513458795824739 1.30148590108328e-26 postive

SCLY GTF3C2-AS1 0.510134334525114 3.09488409097711e-26 postive

BHMT2 SAP30-DT 0.658377968885234 5.93061514078844e-48 postive

DMGDH SAP30-DT 0.582987183846422 1.61552186747458e-35 postive

INMT SAP30-DT 0.534506368534245 4.30593481582894e-29 postive

PPM1K SAP30-DT 0.458733263249063 6.49695003106731e-21 postive

SLC25A12 SAP30-DT 0.591091527900429 1.06273859824111e-36 postive

FTCD LINC01671 0.404307557044194 3.5210405493498e-16 postive

MAT1A LINC01671 0.589842049928967 1.62486827958422e-36 postive

PSPH LINC01671 0.472304757668127 3.11648131354221e-22 postive

CSAD LINC02570 0.410951399771166 1.03196068674098e-16 postive

CSAD SEC62-AS1 0.454238243490869 1.72494262786016e-20 postive

PHYKPL SEC62-AS1 0.470149078663074 5.09482658620188e-22 postive

PPM1K SEC62-AS1 0.49643142860516 9.96534456993066e-25 postive

NAALAD2 SLC25A21-AS1 0.436624618370049 6.90603737480699e-19 postive

SLC25A21 SLC25A21-AS1 0.42144113737796 1.40476914327602e-17 postive

AANAT EML4-AS1 0.413087462441477 6.91445137429323e-17 postive

IL4I1 EML4-AS1 0.507055429314533 6.84539530710668e-26 postive

KMO EML4-AS1 0.716940799444301 2.07110585066851e-60 postive

PPM1K EML4-AS1 0.50545365720171 1.03127387633931e-25 postive

CSAD LINC01004 0.476658487671867 1.1426216857967e-22 postive

PSMC2 LINC01004 0.412818519385921 7.2731399293146e-17 postive

CGA LINC00404 0.56880754604301 1.57889668225672e-33 postive

HAL LINC00404 0.451133083363608 3.35796374757801e-20 postive

TPH1 LINC00404 0.445381775170311 1.13281150368855e-19 postive

NAALAD2 LINC02550 0.402372946803603 5.00786441225763e-16 postive

RPL3L DDN-AS1 0.402059913553063 5.30044684999375e-16 postive

ASPA NR2F2-AS1 0.545185881633598 2.0299674093012e-30 postive

BHMT2 NR2F2-AS1 0.613074836971814 4.42972417955372e-40 postive

CDO1 NR2F2-AS1 0.42867285944165 3.4092540249332e-18 postive

DMGDH NR2F2-AS1 0.643346192701218 3.41377918826719e-45 postive

INMT NR2F2-AS1 0.683813652023586 5.28171238024202e-53 postive

MTR NR2F2-AS1 0.411562808275461 9.2047695927154e-17 postive

PPM1K NR2F2-AS1 0.568711199923927 1.62759929277485e-33 postive

PSMA5 NR2F2-AS1 -0.412480525821295 7.74992330440359e-17 negative

SLC25A12 NR2F2-AS1 0.416604798442107 3.55366009956028e-17 postive

CSAD NARF-IT1 0.480814302909838 4.32609816398994e-23 postive

PHYKPL NARF-IT1 0.445215560747014 1.17292693469431e-19 postive

CSAD ZNF451-AS1 0.49572228685344 1.18765126936396e-24 postive

DMGDH ZNF451-AS1 0.435964470794619 7.89758973197096e-19 postive

RPS27 ZNF236-DT 0.422103870263599 1.23555897251842e-17 postive

RPL3L SRRM2-AS1 0.441663158428734 2.45643097161015e-19 postive

BHMT2 LINC01579 0.572188135406466 5.4032098084073e-34 postive

DMGDH LINC01579 0.481494098695799 3.68594633578436e-23 postive

INMT LINC01579 0.455283008123274 1.3764778064291e-20 postive

SLC25A12 LINC01579 0.449035877347573 5.24566917316721e-20 postive

AANAT CARD11-AS1 0.484464810637378 1.82304658698377e-23 postive

KMO CARD11-AS1 0.497887884757718 6.94126832794268e-25 postive

PHYKPL CARD11-AS1 0.40278364282278 4.64792984541223e-16 postive

PPM1K CARD11-AS1 0.483805301150302 2.13271893847723e-23 postive

GRHPR LINC01484 0.423361172294328 9.67776565693641e-18 postive

PHYKPL LINC01126 0.421201464376016 1.47140839102565e-17 postive

PHYKPL NCBP2-AS1 0.410860420811713 1.04964521773098e-16 postive

AFMID LINC01978 0.578615097743488 6.79645456693707e-35 postive

NAT8L LINC01978 0.436989568157782 6.41155284571408e-19 postive

CSAD TTC3-AS1 0.45238178720096 2.5709424120741e-20 postive

RPL3L TTC3-AS1 0.413152019490765 6.83097187973004e-17 postive

GLUD2 LINC02476 0.409593114186594 1.32923333892562e-16 postive

NAALAD2 LINC02476 0.493954645462824 1.83589755069605e-24 postive

PAH LINC02476 0.433413195132023 1.32276000383917e-18 postive

SLC6A12 LINC02476 0.43130319685667 2.01976725142023e-18 postive

KMO CARD8-AS1 0.545814486952222 1.69012139417195e-30 postive

PPM1K CARD8-AS1 0.640199227744086 1.23487770153458e-44 postive

CSAD GARS1-DT 0.528144105714812 2.52459719606533e-28 postive

PHYKPL GARS1-DT 0.501836248930727 2.58148605979478e-25 postive

ASPA MIR497HG 0.565383369013936 4.61972914993132e-33 postive

BHMT2 MIR497HG 0.830139773408892 1.15222879671644e-96 postive

CDO1 MIR497HG 0.556023680466334 8.16161808583283e-32 postive

DMGDH MIR497HG 0.718836593640741 7.24693927295927e-61 postive

ENOPH1 MIR497HG -0.451338163742377 3.21410602056383e-20 negative

INMT MIR497HG 0.789317238244244 5.0216221521588e-81 postive

PPM1K MIR497HG 0.500966172976743 3.21374541185585e-25 postive

PSMA5 MIR497HG -0.414407106579972 5.39134787713598e-17 negative

PSMD14 MIR497HG -0.488130067326611 7.57577921383659e-24 negative

SLC25A12 MIR497HG 0.439774809083112 3.62601375377871e-19 postive

FTCD DSCR8 0.488967717115948 6.18903642340705e-24 postive

CSAD DNAJC9-AS1 0.506501470007861 7.8896220492576e-26 postive

AANAT MDS2 0.444578607378065 1.34001689156827e-19 postive

KMO MDS2 0.43286865745174 1.47585460799057e-18 postive

RPL3L MDS2 0.461295276844554 3.69986554111569e-21 postive

RPS4Y1 TTTY14 0.59358108723538 4.53552203721221e-37 postive

PHYKPL MANEA-DT 0.41449326278038 5.30427183845599e-17 postive

FTCD MAPK6-DT 0.406797219225697 2.23011586416258e-16 postive

SLC25A15 TSC22D1-AS1 0.413983911702787 5.83986823579555e-17 postive

DIO1 WWOX-AS1 0.576298942125354 1.44228057556893e-34 postive

CSAD FOXO6-AS1 0.425584989477558 6.26677825692123e-18 postive

GLS FOXO6-AS1 0.423860181128883 8.7810538051997e-18 postive

GRHPR EBLN3P 0.407776250840244 1.86155301243599e-16 postive

IARS1 EBLN3P 0.434077511388567 1.15701824244306e-18 postive

PSMD5 EBLN3P 0.402848393410869 4.59354921803688e-16 postive

SECISBP2 EBLN3P 0.469944573841761 5.33705878808821e-22 postive

SEPSECS EBLN3P 0.437552138715018 5.71675060200061e-19 postive

RPL3L KLHL7-DT 0.454451123975801 1.64752672787517e-20 postive

RPL3L LINC02626 0.534024219760267 4.93002939189582e-29 postive

CSAD ODF2-AS1 0.477022128598333 1.05008179098632e-22 postive

DIO3 DIO3OS 0.434674015547012 1.0257113414889e-18 postive

GNMT LINC01545 0.404548482387113 3.3693780040221e-16 postive

CSAD PTOV1-AS2 0.425757675562281 6.05801501893744e-18 postive

PHYKPL PTOV1-AS2 0.511568156846417 2.1325322877528e-26 postive

CSAD EHMT2-AS1 0.443354245880731 1.72961326904087e-19 postive

AMDHD1 WASIR2 0.407111971254948 2.10442648200816e-16 postive

RPL3L WASIR2 0.425221579709375 6.72948205002021e-18 postive

AIMP2 RNASEH1-AS1 0.466232200628775 1.23352579622496e-21 postive

RPL3L RNASEH1-AS1 0.443849156698744 1.56026998328645e-19 postive

ASPG LINC02487 0.479818246476874 5.4666229834208e-23 postive

BBOX1 LINC02487 0.695976480575645 1.32118205241647e-55 postive

DUOX1 LINC02487 0.593559843584403 4.56873978468152e-37 postive

SLC6A11 LINC02487 0.504602141651291 1.28116592313789e-25 postive

ASPG MIR205HG 0.405397210843265 2.88446952636319e-16 postive

BBOX1 MIR205HG 0.829344541569588 2.54133649073138e-96 postive

DUOX1 MIR205HG 0.717582689798226 1.45281623936066e-60 postive

SLC6A11 MIR205HG 0.605339138038854 7.34192812134026e-39 postive

GAMT LINC01767 0.59528960002479 2.51755094911845e-37 postive

PAH LINC01767 0.55243350200983 2.39761290310933e-31 postive

DMGDH HOMER3-AS1 0.49347416029831 2.06574398590669e-24 postive

RPL3L HOMER3-AS1 0.405772129556195 2.6927385072189e-16 postive

CSAD NEAT1 0.563823468538471 7.50291025254727e-33 postive

AGMAT LINC01106 0.412592800412732 7.58823647306274e-17 postive

RPL3L HCG20 0.461301560795426 3.69473804604601e-21 postive

SEM1 RAB11B-AS1 0.519272831315472 2.79569211413831e-27 postive

AASS ADAMTS9-AS1 0.437572972008647 5.69249605623965e-19 postive

ASPA ADAMTS9-AS1 0.480412524436225 4.75475197742618e-23 postive

BHMT2 ADAMTS9-AS1 0.626740856050808 2.55717879487715e-42 postive

CDO1 ADAMTS9-AS1 0.400867735399412 6.57644576394578e-16 postive

DMGDH ADAMTS9-AS1 0.638564565554539 2.39391121289283e-44 postive

INMT ADAMTS9-AS1 0.593683640348513 4.37849675783791e-37 postive

NAALAD2 LINC01807 0.401604337029121 5.756455369339e-16 postive

SERINC3 STK4-AS1 0.537263389884961 1.97750926177061e-29 postive

AGMAT OGFRP1 0.427829602341558 4.02836459879873e-18 postive

SLC25A15 OGFRP1 0.434024100715249 1.16955171499298e-18 postive

ASL STPG3-AS1 0.41464016309629 5.1589782762322e-17 postive

RPL6 MAPKAPK5-AS1 0.450557279568901 3.79662778633422e-20 postive

CSAD RHOA-IT1 0.520380729337651 2.07846839910934e-27 postive

PHYKPL RHOA-IT1 0.419409434513756 2.07837335013209e-17 postive

PPM1K RHOA-IT1 0.401031350229619 6.38494301037638e-16 postive

AANAT LINC00243 0.447792672843511 6.82352389894887e-20 postive

KMO LINC00243 0.526200009658592 4.30211453259684e-28 postive

PPM1K LINC00243 0.510603347116266 2.74043530389106e-26 postive

RPL3L ODC1-DT 0.451625499335455 3.02270504914627e-20 postive

SLC7A5 LINC00592 0.408915967893444 1.5073772690207e-16 postive

FTCD RN7SL832P 0.466934551434331 1.05354100850029e-21 postive

RPL3L RN7SL832P 0.549823264253492 5.20597993455513e-31 postive

HAL DNAJC3-DT 0.527624659205925 2.91199569715795e-28 postive

IL4I1 LINC01943 0.628922990159746 1.09633428788383e-42 postive

PSMB10 LINC01943 0.433510003283034 1.29722820397527e-18 postive

PSMB9 LINC01943 0.479887044818976 5.37911277194652e-23 postive

RPL3L UVRAG-DT 0.579914527453464 4.44440142228646e-35 postive

HOGA1 HS1BP3-IT1 0.414633154129324 5.16582089044299e-17 postive

NAALAD2 HS1BP3-IT1 0.432527341567928 1.58056374184796e-18 postive

TPH1 MIR548XHG 0.55374924204525 1.61778773796567e-31 postive

FTCD TSPEAR-AS2 0.606583266435699 4.69832910995268e-39 postive

BHMT2 FTX 0.438425418591157 4.78232435171589e-19 postive

CSAD FTX 0.44180874586261 2.38353516779602e-19 postive

DMGDH FTX 0.488600070602995 6.7637930205828e-24 postive

ECHS1 FTX -0.40359640238229 4.00887243740809e-16 negative

GOT1 FTX -0.421949814492056 1.27301012310461e-17 negative

INMT FTX 0.563041880388038 9.55736959551567e-33 postive

MTR FTX 0.407827851798752 1.84388295105878e-16 postive

PHYKPL FTX 0.400620876338437 6.87609515485775e-16 postive

PPM1K FTX 0.519239964806278 2.82033997382379e-27 postive

PSMA5 FTX -0.403681770354353 3.9469796018999e-16 negative

AFMID ZNF197-AS1 0.449686163058477 4.56956950524678e-20 postive

NAT8L ZNF197-AS1 0.461935139910899 3.21213650731079e-21 postive

RPL3L LIF-AS1 0.415678285427355 4.23793054935286e-17 postive

RPL19 LINC00392 0.656176554069714 1.53890198295122e-47 postive

CGA SOX1-OT 0.572703846219983 4.58283705783378e-34 postive

HAL SOX1-OT 0.469635060056487 5.72541538899403e-22 postive

FTCD STK24-AS1 0.611841338899393 6.96753516770661e-40 postive

FTCD UBAC2-AS1 0.492824871149832 2.42199653830704e-24 postive

RPL3L UBAC2-AS1 0.493313426856345 2.14879710698074e-24 postive

AGXT C5orf34-AS1 0.505676362670909 9.74282880567759e-26 postive

PAH TPT1-AS1 0.403068344705041 4.41344628104581e-16 postive

PIPOX TPT1-AS1 0.404763538019434 3.23942946517436e-16 postive

PHYKPL MATN1-AS1 0.411173673846675 9.89976434148734e-17 postive

BHMT2 GIHCG 0.440994754399684 2.82025922805357e-19 postive

DMGDH GIHCG 0.435784421496285 8.19150912078856e-19 postive

OAZ3 TDRKH-AS1 0.400725192731215 6.74787073845566e-16 postive

PSMB4 TDRKH-AS1 0.481153521720174 3.99403231762323e-23 postive

PSMD4 TDRKH-AS1 0.540702570531977 7.41596808900658e-30 postive

PYCR2 TDRKH-AS1 0.447768179425173 6.8588945038977e-20 postive

SMOX TDRKH-AS1 0.432615390947417 1.55287272554773e-18 postive

CSAD LINC02878 0.420244197553456 1.76997253486304e-17 postive

PPM1K RRN3P2 0.450889496125186 3.53708600979961e-20 postive

CSAD LINC01138 0.43416256199777 1.13733223932966e-18 postive

FTCD LRP1-AS 0.408595844117409 1.5995616847501e-16 postive

TPO LRP1-AS 0.416330434902132 3.74410118090707e-17 postive

AASS RAP2C-AS1 0.447613998748398 7.08572507415519e-20 postive

ASPA RAP2C-AS1 0.457133785074041 9.21139491311673e-21 postive

BHMT2 RAP2C-AS1 0.773868192681144 5.47816113695352e-76 postive

CDO1 RAP2C-AS1 0.442592098644646 2.02636950471029e-19 postive

CKB RAP2C-AS1 0.413063690512643 6.94544288900854e-17 postive

DMGDH RAP2C-AS1 0.65547948717954 2.07784985873584e-47 postive

ECHS1 RAP2C-AS1 -0.41146214806289 9.37981256382778e-17 negative

ENOPH1 RAP2C-AS1 -0.419942393706226 1.87589788330197e-17 negative

GOT1 RAP2C-AS1 -0.411360463059421 9.5599558998475e-17 negative

INMT RAP2C-AS1 0.70017542009159 1.55672547614746e-56 postive

MTR RAP2C-AS1 0.541464451341147 5.95857785127389e-30 postive

PPM1K RAP2C-AS1 0.530266106720866 1.40541766332703e-28 postive

PSMA5 RAP2C-AS1 -0.42548574443064 6.38994078154823e-18 negative

PSMD14 RAP2C-AS1 -0.421213040975118 1.46811937185634e-17 negative

SERINC1 RAP2C-AS1 0.463876054467017 2.08823871415258e-21 postive

SLC25A10 RAP2C-AS1 -0.449033552066332 5.24825524292745e-20 negative

SLC25A12 RAP2C-AS1 0.582579106260214 1.84906158869696e-35 postive

AIMP1 SNHG1 0.420238066112748 1.77206484337551e-17 postive

DLAT SNHG1 0.417646915917376 2.91321347416716e-17 postive

EEF1E1 SNHG1 0.476355110658903 1.22594125861418e-22 postive

ENOPH1 SNHG1 0.449712964649858 4.54362720130524e-20 postive

PSMA1 SNHG1 0.543717650964011 3.10950309953088e-30 postive

PSMA3 SNHG1 0.467390534417225 9.5081709777327e-22 postive

PSMC6 SNHG1 0.442518821769361 2.05741264201682e-19 postive

PSMD14 SNHG1 0.548986462461344 6.6654002550482e-31 postive

RPL22 SNHG1 0.41242754161961 7.82739649452648e-17 postive

RPL31 SNHG1 0.414585031621089 5.21304258115124e-17 postive

RPS12 SNHG1 0.454364156354236 1.67873094879679e-20 postive

RPS13 SNHG1 0.445164379300642 1.1855586526394e-19 postive

RPS18 SNHG1 0.406316911119703 2.4362474001524e-16 postive

RPS7 SNHG1 0.428299083980016 3.67118788191208e-18 postive

SCLY SNHG1 0.484835546429163 1.66890301113772e-23 postive

MTR ZKSCAN7-AS1 0.432377958893381 1.62865871635498e-18 postive

TH LINC01607 0.428711531235729 3.3832262789819e-18 postive

RPL19 FOCAD-AS1 0.61779605064706 7.68113659978091e-41 postive

LIPT1 LINC01473 0.40992727261719 1.24911155880655e-16 postive

CSAD LINC02614 0.423595465417295 9.24606306682388e-18 postive

NAALAD2 PCAT1 0.40815563782801 1.73542114055941e-16 postive

GOT2 TYMSOS 0.447375391594437 7.45139762131495e-20 postive

GSTZ1 TYMSOS 0.417119782617183 3.22154861207149e-17 postive

NDUFAB1 TYMSOS 0.453588331764211 1.98413215752526e-20 postive

RPL26L1 TYMSOS 0.425089087734559 6.90639596170037e-18 postive

OAZ3 TESC-AS1 0.405529675908744 2.81524410127732e-16 postive

ASPA LINC01537 0.520758132138483 1.87835836426709e-27 postive

BHMT2 LINC01537 0.665238888231591 2.88285884618402e-49 postive

DMGDH LINC01537 0.625019150115345 4.96495481743735e-42 postive

INMT LINC01537 0.549680086370436 5.43109184680057e-31 postive

PPM1K LINC01537 0.425042893442684 6.96914686937806e-18 postive

PSMD14 LINC01537 -0.409179010988174 1.43555162588499e-16 negative

SLC25A12 LINC01537 0.419044584808865 2.22921186956945e-17 postive

CSAD INE1 0.459207228548166 5.85637033342228e-21 postive

PHYKPL INE1 0.469801733596922 5.51294765310916e-22 postive

AFMID SEC24B-AS1 0.532793324798779 6.95806861656314e-29 postive

LIPT1 SEC24B-AS1 0.406555289972184 2.33170741404191e-16 postive

NAT8L SEC24B-AS1 0.493231733799443 2.19226303657535e-24 postive

DIO1 LINC01088 0.474057274222881 2.08451593596986e-22 postive

RPS4Y1 LINC00278 0.624009751614315 7.3116939675339e-42 postive

CSAD TXNDC12-AS1 0.409180735618015 1.43509197607665e-16 postive

CGA NNT-AS1 0.403300883301176 4.23057350659659e-16 postive

RPL37 NNT-AS1 0.511889911227392 1.96107113565872e-26 postive

KMO KIF1C-AS1 0.455926241232775 1.19746300210496e-20 postive

PHYKPL KIF1C-AS1 0.477540964265193 9.30708929707967e-23 postive

PPM1K KIF1C-AS1 0.458733065458998 6.49723125179411e-21 postive

BHMT2 PGM5-AS1 0.714267681318628 8.97255651261944e-60 postive

CKB PGM5-AS1 0.430371680185886 2.4324595830416e-18 postive

DMGDH PGM5-AS1 0.53744237212199 1.87962694556132e-29 postive

INMT PGM5-AS1 0.527247013981874 3.22996276797514e-28 postive

SLC25A12 PGM5-AS1 0.59376543432087 4.25712483155307e-37 postive

CSAD SND1-IT1 0.44515617675913 1.18759547203009e-19 postive

PSMD4 LINC00337 0.42680419294947 4.93150374603149e-18 postive

RPL3L LINC00337 0.438784102298033 4.44363344037627e-19 postive

AFMID MHENCR 0.633986236262293 1.49658693475984e-43 postive

NAT8L MHENCR 0.604682895939901 9.28380036928361e-39 postive

PHYKPL AP4B1-AS1 0.519791161634826 2.43396504637136e-27 postive

RPL3L RB1-DT 0.468013123682573 8.26333566215442e-22 postive

AIMP1 OTUD6B-AS1 0.433104037688481 1.40765060073373e-18 postive

AZIN1 OTUD6B-AS1 0.42348206694065 9.45259095270468e-18 postive

RIDA OTUD6B-AS1 0.528160355770463 2.51333766883925e-28 postive

RPL30 OTUD6B-AS1 0.496367014661279 1.0125602640258e-24 postive

RPL7 OTUD6B-AS1 0.403857213418066 3.8227111202243e-16 postive

RPL3L IL6R-AS1 0.44718200187809 7.76134906259032e-20 postive

CSAD PSMA3-AS1 0.517806843869608 4.13165106087335e-27 postive

KMO PSMA3-AS1 0.400409239118528 7.14362655362786e-16 postive

LIPT1 PSMA3-AS1 0.50175130247694 2.63736709330713e-25 postive

PHYKPL PSMA3-AS1 0.518473595160924 3.4599806173415e-27 postive

PPM1K PSMA3-AS1 0.406099608220171 2.53554540515774e-16 postive

EEF1E1 SNHG10 0.402224974982443 5.14413819665328e-16 postive

PSMA3 SNHG10 0.405710611903997 2.72331703443314e-16 postive

PSMD14 SNHG10 0.422499619882326 1.14424457996288e-17 postive

RPL3L SNHG10 0.401188345586805 6.20633754232274e-16 postive

CSAD LENG8-AS1 0.508194776036567 5.10776009211963e-26 postive

PHYKPL LENG8-AS1 0.434328176636568 1.09993941485878e-18 postive

BBOX1 FAM83A-AS1 0.407364588491682 2.00860374269768e-16 postive

DUOX1 FAM83A-AS1 0.534876221207313 3.88071815249302e-29 postive

AANAT PIK3CD-AS1 0.490916019066894 3.85887197610734e-24 postive

KMO PIK3CD-AS1 0.629003940354952 1.0622866983936e-42 postive

PHYKPL PIK3CD-AS1 0.40151024985159 5.85531997318866e-16 postive

PPM1K PIK3CD-AS1 0.669580709773873 4.07903591028806e-50 postive

AASS NR2F1-AS1 0.419278888152438 2.13115550382791e-17 postive

AHCY NR2F1-AS1 -0.409630896356466 1.31992709156783e-16 negative

ASPA NR2F1-AS1 0.493773968565591 1.91920440230765e-24 postive

BHMT2 NR2F1-AS1 0.771824400540905 2.37222521090835e-75 postive

CDO1 NR2F1-AS1 0.43581692238118 8.13766908962341e-19 postive

DMGDH NR2F1-AS1 0.69281700904018 6.44568516821187e-55 postive

INMT NR2F1-AS1 0.701600278455638 7.47059282776867e-57 postive

PPM1K NR2F1-AS1 0.435207053406432 9.20849761437936e-19 postive

SERINC1 NR2F1-AS1 0.436961685576181 6.44806990531412e-19 postive

SLC25A10 NR2F1-AS1 -0.454908364646564 1.49262899834574e-20 negative

SLC25A12 NR2F1-AS1 0.530688995297765 1.24995504951849e-28 postive

NAALAD2 EIF2AK3-DT 0.411690396542589 8.98750451067735e-17 postive

LIPT2 PXN-AS1 0.402795622287188 4.6378215083765e-16 postive

RPL39L PXN-AS1 0.408719177224407 1.56340847741555e-16 postive

DIO1 LINC02716 0.499872062469205 4.22926392559086e-25 postive

TH LNCAROD 0.532087364802253 8.47296502087456e-29 postive

OAZ3 ZNF687-AS1 0.451161229583197 3.33785097615827e-20 postive

PSMB4 ZNF687-AS1 0.48679157000646 1.0452755596608e-23 postive

PSMD4 ZNF687-AS1 0.544898441784493 2.20708163327082e-30 postive

RPL39L ZNF687-AS1 0.401861775859367 5.49424226304936e-16 postive

RPL3L ZNF687-AS1 0.482228534847852 3.09910914025157e-23 postive

CSAD UBOX5-AS1 0.434018208049759 1.1709426541611e-18 postive

RPL3L UBOX5-AS1 0.488421471693851 7.06169707655376e-24 postive

SCLY UBOX5-AS1 0.479191482965058 6.33132372051049e-23 postive

ASPA LINC01140 0.526469294501654 3.99674516029368e-28 postive

BHMT2 LINC01140 0.545704682639674 1.74513661134452e-30 postive

CDO1 LINC01140 0.550707645691754 4.00633816484478e-31 postive

DIO2 LINC01140 0.435405936761841 8.84488797761629e-19 postive

DMGDH LINC01140 0.578864398000952 6.26549368465184e-35 postive

INMT LINC01140 0.578777695561805 6.44532636814316e-35 postive

DBH CASC15 0.560608600438795 2.02203271896851e-32 postive

RPL3L PRKX-AS1 0.477221781290818 1.00245789871664e-22 postive

RPL3L USP12-AS2 0.639822858228631 1.43869800067442e-44 postive

RPL3L DAPK1-IT1 0.420234996180159 1.77311334796727e-17 postive

OAZ3 PDC-AS1 0.437561756960359 5.70554023665429e-19 postive

AANAT CEP250-AS1 0.454812774422122 1.52377828565328e-20 postive

KMO CEP250-AS1 0.616435169571481 1.27670673187238e-40 postive

PHYKPL CEP250-AS1 0.406489155944244 2.36026046208743e-16 postive

PPM1K CEP250-AS1 0.678343659007832 7.1107062809647e-52 postive

ASPA MSC-AS1 0.414055758282721 5.76121998938384e-17 postive

AZIN2 MSC-AS1 0.407918019749074 1.81340060610029e-16 postive

BHMT2 MSC-AS1 0.464006155480875 2.02862483710459e-21 postive

CDO1 MSC-AS1 0.429888922009071 2.67789353591774e-18 postive

DIO2 MSC-AS1 0.531195436781034 1.08600097674234e-28 postive

DMGDH MSC-AS1 0.49357269982946 2.01640177200582e-24 postive

INMT MSC-AS1 0.562633684020402 1.0842318639248e-32 postive

NNMT MSC-AS1 0.489375769374469 5.60741216229383e-24 postive

SERINC1 MSC-AS1 0.433383644953858 1.33065144728011e-18 postive

SLC25A10 MSC-AS1 -0.432246845752894 1.67205569123778e-18 negative

RPL3L ITCH-AS1 0.415033899761005 4.78855539198795e-17 postive

RPL36 ELFN1-AS1 0.411347021366897 9.58402178853065e-17 postive

RPS7 ELFN1-AS1 0.419400119430351 2.08209665329903e-17 postive

MAT1A CASC2 0.453385393180737 2.07265888957845e-20 postive

CSAD LINC00894 0.492373529254592 2.70469471558891e-24 postive

PHYKPL LINC00894 0.470294564697418 4.92912977061567e-22 postive

BHMT2 ZNF667-AS1 0.625004900563693 4.99220800250557e-42 postive

CDO1 ZNF667-AS1 0.486786830713 1.04646501671355e-23 postive

CKB ZNF667-AS1 0.466334118563427 1.20564233112609e-21 postive

DMGDH ZNF667-AS1 0.486781181618362 1.04788455769465e-23 postive

INMT ZNF667-AS1 0.480664994962649 4.4807500545406e-23 postive

CSAD SLFNL1-AS1 0.466337000071553 1.20486308252146e-21 postive

PHYKPL SLFNL1-AS1 0.403691608539827 3.93990735253489e-16 postive

IDO1 DLGAP1-AS5 0.476942459434289 1.06970212235243e-22 postive

PSME2 DLGAP1-AS5 0.425412998372795 6.48172649277068e-18 postive

FTCD TSPEAR-AS1 0.554693682163513 1.21846067202367e-31 postive

BHMT2 LINC01589 0.400860931925611 6.58452976523819e-16 postive

DMGDH LINC01589 0.445832123718052 1.03078319171466e-19 postive

INMT LINC01589 0.45781100477811 7.94746358626288e-21 postive

CSAD LINC00653 0.426756124612262 4.97840497157145e-18 postive

NAALAD2 LINC00653 0.510421945777322 2.87250282914474e-26 postive

CSAD FAM215B 0.401215269723147 6.17620383738637e-16 postive

KMO MMP2-AS1 0.497081127011955 8.48255368698935e-25 postive

CSAD ASMTL-AS1 0.493757943913594 1.92677066938751e-24 postive

PHYKPL ASMTL-AS1 0.412828970134861 7.25886657341031e-17 postive

GLS MIR194-2HG 0.495676260103976 1.20123596911495e-24 postive

CSAD SCAT2 0.436596942121085 6.94503165702328e-19 postive

DARS1 SCAT2 0.416694466035808 3.4935085560011e-17 postive

EEF1E1 SCAT2 0.516336334010059 6.10177943292792e-27 postive

LIPT1 SCAT2 0.409428750223876 1.37047380112754e-16 postive

PSMA2 SCAT2 0.458520060948388 6.80714906499683e-21 postive

PSMD1 SCAT2 0.440601434905457 3.05861476397724e-19 postive

RPL22 SCAT2 0.413020493153722 7.00210898903708e-17 postive

RPL31 SCAT2 0.475824417011755 1.38633216397488e-22 postive

RPL39 SCAT2 0.447687218976649 6.97709745873049e-20 postive

KMO PSMB8-AS1 0.444876707742297 1.25908788331773e-19 postive

PSMB8 PSMB8-AS1 0.609316392301475 1.74999754905154e-39 postive

PSMB9 PSMB8-AS1 0.586879671774817 4.41370387692711e-36 postive

BHMT2 LRRC3-DT 0.599083908600327 6.7255260866621e-38 postive

DMGDH LRRC3-DT 0.641155484887764 8.36833729142921e-45 postive

INMT LRRC3-DT 0.42632069266996 5.42374075762162e-18 postive

SLC25A12 LRRC3-DT 0.444362461349268 1.40187749140372e-19 postive

CKM NCOA7-AS1 0.672713619622162 9.74453235532265e-51 postive

SLC5A5 NCOA7-AS1 0.626859140804818 2.44285677345545e-42 postive

RPL3L THORLNC 0.473289266974699 2.48696760747196e-22 postive

ASPA HHIP-AS1 0.408156314535043 1.73520383615864e-16 postive

BHMT2 HHIP-AS1 0.415831798357435 4.11622058345093e-17 postive

CDO1 HHIP-AS1 0.645736557197523 1.27261141822512e-45 postive

DIO2 HHIP-AS1 0.467760467748091 8.747851925757e-22 postive

DMGDH HHIP-AS1 0.427508016415466 4.2925209850447e-18 postive

INMT HHIP-AS1 0.571328868475591 7.10448828850951e-34 postive

KMO ARAP1-AS2 0.405450145335657 2.85660810320418e-16 postive

PHYKPL ARAP1-AS2 0.490254226012814 4.53207172178169e-24 postive

CSAD DIP2A-IT1 0.469824379352349 5.48468615501402e-22 postive

NAALAD2 LINC02696 0.424425027326536 7.8643358786944e-18 postive

GLS SEMA6A-AS1 0.416660418174101 3.51623008827265e-17 postive

PHYKPL SEMA6A-AS1 0.400745858190235 6.72274891827335e-16 postive

DBH MIR924HG 0.450434848251952 3.89693065398303e-20 postive

OAZ3 INTS6-AS1 0.405713958567373 2.721644778615e-16 postive

RPL3L INTS6-AS1 0.446052159412964 9.84277869319159e-20 postive

PSMB7 SNHG15 0.443867851786184 1.5542036782787e-19 postive

RPL30 SNHG15 0.429558996030504 2.85947309637931e-18 postive

RPL31 SNHG15 0.400433969116798 7.11184580608218e-16 postive

RPL35 SNHG15 0.406610214921765 2.30825182879137e-16 postive

RPL36A SNHG15 0.414604114341448 5.19426655573023e-17 postive

RPL7 SNHG15 0.418035008659562 2.70492603091639e-17 postive

RPL7A SNHG15 0.416627472810457 3.53835406514162e-17 postive

RPL8 SNHG15 0.434282565014487 1.11011531968777e-18 postive

RPS18 SNHG15 0.400706853192177 6.77024220735123e-16 postive

RPS7 SNHG15 0.456026392428273 1.17173563111513e-20 postive

GLS LINC01355 0.433492035889716 1.30192993662637e-18 postive

LIPT1 LINC01355 0.44943044853073 4.8245343642163e-20 postive

KMO RASSF1-AS1 0.426169445320139 5.58741307558624e-18 postive

PPM1K RASSF1-AS1 0.408829506025799 1.53174799330233e-16 postive

CKMT1A KBTBD11-OT1 0.401225994254461 6.16424088487909e-16 postive

CKMT1B KBTBD11-OT1 0.404946524229187 3.13274296865671e-16 postive

PSMD1 VASH1-AS1 0.444345313563051 1.40690378881249e-19 postive

RPL3L VASH1-AS1 0.416567638012343 3.57888553432303e-17 postive

SERINC3 NORAD 0.575991659362536 1.59296668622877e-34 postive

CSAD FOXP1-AS1 0.468955125537635 6.67899432873838e-22 postive

PPM1K FOXP1-AS1 0.423174194106897 1.0036447630785e-17 postive

RPL39L LINC02321 0.40405695708467 3.68590141315088e-16 postive

SCLY LINC01311 0.404936807312417 3.13832047721274e-16 postive

RPL3L P3H2-AS1 0.403717782305628 3.92115266299857e-16 postive

AASS GAS1RR 0.409341405531372 1.39289800755924e-16 postive

ASPA GAS1RR 0.526672294847609 3.78079224779208e-28 postive

BHMT2 GAS1RR 0.789189732711786 5.54811821089509e-81 postive

CDO1 GAS1RR 0.586602323993324 4.84402405522859e-36 postive

DMGDH GAS1RR 0.723315882612224 5.85501971584413e-62 postive

INMT GAS1RR 0.74793999240421 2.25269266426643e-68 postive

MTR GAS1RR 0.405027443088973 3.08667190845311e-16 postive

PPM1K GAS1RR 0.498964592739193 5.30700742331039e-25 postive

PSMD14 GAS1RR -0.430526475389407 2.35855053288271e-18 negative

SLC25A10 GAS1RR -0.412463719263151 7.7744158387531e-17 negative

SLC25A12 GAS1RR 0.537762475765052 1.71636135542944e-29 postive

RPL3L RPP38-DT 0.432105683779434 1.72005030772744e-18 postive

AFMID LINC01979 0.400921554407302 6.51283945079413e-16 postive

AASS MIR100HG 0.431784282445997 1.83443717211737e-18 postive

ASPA MIR100HG 0.503903689860184 1.53003389989584e-25 postive

BHMT2 MIR100HG 0.83926628456741 9.69576918470033e-101 postive

CDO1 MIR100HG 0.467514421155603 9.24656387373564e-22 postive

DMGDH MIR100HG 0.696701897783697 9.15503973539866e-56 postive

ENOPH1 MIR100HG -0.401624298524713 5.73569191920815e-16 negative

INMT MIR100HG 0.745112559068007 1.33876857066606e-67 postive

MTR MIR100HG 0.418834282063265 2.3209906473705e-17 postive

PPM1K MIR100HG 0.472995319158086 2.66049098528977e-22 postive

PSMD14 MIR100HG -0.417563042707372 2.96025711220206e-17 negative

SERINC1 MIR100HG 0.536419901572964 2.51096984166098e-29 postive

SLC25A10 MIR100HG -0.452926471685198 2.28744516095076e-20 negative

SLC25A12 MIR100HG 0.601269083518614 3.11962637992003e-38 postive

KMO WAKMAR2 0.543107194239153 3.71044415197481e-30 postive

PPM1K WAKMAR2 0.575301027520708 1.99084704062286e-34 postive

ASPA LINC02106 0.406065147999869 2.5516530225069e-16 postive

BHMT2 LINC02106 0.740911502808871 1.81138999689855e-66 postive

CKB LINC02106 0.409782491879336 1.2832262146724e-16 postive

DMGDH LINC02106 0.598965864736781 7.00931919975789e-38 postive

INMT LINC02106 0.666692210113998 1.50364627472953e-49 postive

PPM1K LINC02106 0.473384442210966 2.43321189352284e-22 postive

SLC25A12 LINC02106 0.538226766127276 1.50416397528594e-29 postive

CSAD EXTL3-AS1 0.412931958712545 7.11967085694223e-17 postive

RPL3L EXTL3-AS1 0.472251827302292 3.15445290595291e-22 postive

GLS PRKCZ-AS1 0.445681015589295 1.06396623074643e-19 postive

PHYKPL PRKCZ-AS1 0.402707167744626 4.71297146607379e-16 postive

BHMT2 MIR1-1HG-AS1 0.816949023799058 3.46843144338678e-91 postive

CKB MIR1-1HG-AS1 0.471455358992323 3.78404459665188e-22 postive

DMGDH MIR1-1HG-AS1 0.640382973022419 1.1460406021426e-44 postive

INMT MIR1-1HG-AS1 0.589603084723169 1.76194440078408e-36 postive

MTR MIR1-1HG-AS1 0.413733045969776 6.12284217969092e-17 postive

PPM1K MIR1-1HG-AS1 0.402593503391692 4.8112960531992e-16 postive

SLC25A12 MIR1-1HG-AS1 0.653399415525451 5.06633047403048e-47 postive

GLS NADK2-AS1 0.41725158292756 3.14157471506079e-17 postive

MTR PAXBP1-AS1 0.419040908864346 2.23078505028152e-17 postive

NAALAD2 PAXBP1-AS1 0.457421103103607 8.65264797191356e-21 postive

ASPA LINC01082 0.472012728341664 3.33174039220551e-22 postive

BHMT2 LINC01082 0.470313409646222 4.90805922503337e-22 postive

DIO2 LINC01082 0.518226978951353 3.69481349453659e-27 postive

DMGDH LINC01082 0.434078590500031 1.15676638205577e-18 postive

HDC LINC01082 0.450403701598945 3.92286129738736e-20 postive

CSAD C3orf35 0.433198626639681 1.3811242016011e-18 postive

AFMID LINC02473 0.485721449049423 1.35070784731262e-23 postive

NAT8L LINC02473 0.495806463421541 1.16319771117307e-24 postive

BHMT2 LINC00702 0.54943470942836 5.83947796857297e-31 postive

DMGDH LINC00702 0.438092688405863 5.11914882180042e-19 postive

SLC25A12 LINC00702 0.417141023689832 3.20852606066931e-17 postive

PSMD4 KCNMB2-AS1 0.404986180665501 3.11008089771084e-16 postive

PSME4 UBE2Q1-AS1 0.402138239398606 5.22570179191957e-16 postive

CSAD HCG25 0.531778128692784 9.23516560171105e-29 postive

PHYKPL HCG25 0.419962467454046 1.86866317427572e-17 postive

PPM1K HCG25 0.401140165754668 6.26062129949178e-16 postive

BHMT2 LINC02593 0.465545923155055 1.43854995188634e-21 postive

DIO2 LINC02593 0.418053004959325 2.69563102449361e-17 postive

DMGDH LINC02593 0.439986200850904 3.47175545066212e-19 postive

INMT LINC02593 0.523272166002264 9.538993967425e-28 postive

CSAD CTBP1-AS 0.45343077205326 2.05253194902554e-20 postive

PHYKPL CTBP1-AS 0.618351915504999 6.23708441283029e-41 postive

PSMB8 HCP5 0.697671448646198 5.5975696104638e-56 postive

PSMB9 HCP5 0.597266155456796 1.26855117517856e-37 postive

PSME1 HCP5 0.401332309812024 6.04687179106708e-16 postive

CSAD MIS18A-AS1 0.427176104922873 4.58301070847863e-18 postive

OAZ3 MIS18A-AS1 0.45891359406201 6.24547860875104e-21 postive

PSMD4 MIS18A-AS1 0.423269148600515 9.85269238111296e-18 postive

RPL3L MIS18A-AS1 0.523557500662566 8.8297411882493e-28 postive

DIO1 STAU2-AS1 0.420020227709102 1.84799861099381e-17 postive

CSAD NARF-AS1 0.419983551661364 1.86109381297998e-17 postive

HOGA1 ITGB1-DT 0.408813231681482 1.53637826095765e-16 postive

BCAT1 HHLA3-AS1 0.807688370032994 1.34435904290676e-87 postive

CTH HHLA3-AS1 0.899319784518926 4.66909427577866e-136 postive

PYCR1 RHPN1-AS1 0.408206084484043 1.71929472022927e-16 postive

SLC3A2 RHPN1-AS1 0.412705812169501 7.42883501766784e-17 postive

GRHPR LINC01679 0.440665654033957 3.01838615654168e-19 postive

SLC6A7 LINC00668 0.42436604156338 7.95548667999774e-18 postive

FTCD CEP83-DT 0.49228572399154 2.76335884510273e-24 postive

NAALAD2 CEP83-DT 0.571072193809076 7.70861680772782e-34 postive

RPL3L CEP83-DT 0.456746050511625 1.00221084879512e-20 postive

CSAD REV3L-IT1 0.461769363633186 3.33205276747644e-21 postive

PSMD1 REV3L-IT1 0.400552644326272 6.96125536201894e-16 postive

GCDH LMF1-AS1 0.400911993068885 6.52409530617484e-16 postive

AASS SNHG14 0.434352024228615 1.09465562434136e-18 postive

BHMT2 SNHG14 0.630325714991941 6.33797111697328e-43 postive

CDO1 SNHG14 0.517076630404946 5.01547997410334e-27 postive

CKB SNHG14 0.479490119284434 5.90369069170655e-23 postive

DMGDH SNHG14 0.563441476179711 8.44569256721177e-33 postive

INMT SNHG14 0.493301714886763 2.15497601188376e-24 postive

MTR SNHG14 0.511383896945381 2.23730289467279e-26 postive

PPM1K SNHG14 0.47284128297534 2.75613244703097e-22 postive

PSMA5 SNHG14 -0.427130013354666 4.62484898685915e-18 negative

SLC25A12 SNHG14 0.487927851241343 7.95404136346662e-24 postive

CSAD STAG3L5P-PVRIG2P-PILRB 0.633771071826825 1.62997643943426e-43 postive

PHYKPL STAG3L5P-PVRIG2P-PILRB 0.459365154637838 5.65707384107344e-21 postive

SEM1 STAG3L5P-PVRIG2P-PILRB 0.437186978520338 6.1587655363557e-19 postive

ACMSD KLHL6-AS1 0.488249216022528 7.36126792012054e-24 postive

HOGA1 LNCOG 0.419353706491219 2.10074613483951e-17 postive

TPH1 HCG18 0.53658144458447 2.39882907163236e-29 postive

CSAD ASH1L-IT1 0.427426666352913 4.36199804301694e-18 postive

CSAD HM13-IT1 0.538788689615051 1.28177113382866e-29 postive

SCLY HM13-IT1 0.426303198974612 5.44242780503388e-18 postive

BHMT2 NALT1 0.731457690314283 5.31242946182479e-64 postive

CKB NALT1 0.450906706497056 3.52412727795276e-20 postive

DMGDH NALT1 0.56808863773313 1.98014793159205e-33 postive

INMT NALT1 0.487644156847195 8.5162977968912e-24 postive

SLC25A12 NALT1 0.627421328017544 1.96505688590636e-42 postive

HAAO LINC02728 0.416147889578201 3.87632764709373e-17 postive

SEM1 LINC02595 0.424130794341659 8.32945812279822e-18 postive

AIMP2 SEMA3F-AS1 -0.403065238766308 4.41594061263355e-16 negative

CSAD SEMA3F-AS1 0.527842579731656 2.74279793698215e-28 postive

PHYKPL SEMA3F-AS1 0.571538650551718 6.64570926542353e-34 postive

PPM1K SEMA3F-AS1 0.423707742589052 9.04594907806716e-18 postive

PSMD8 SEMA3F-AS1 -0.400315444373113 7.26543465071218e-16 negative

CSAD MRTFA-AS1 0.446759839195425 8.48272401541198e-20 postive

AMT DLGAP4-AS1 0.401734939857259 5.62193137748875e-16 postive

CSAD DLGAP4-AS1 0.453917587528444 1.84836037134159e-20 postive

LIPT1 DLGAP4-AS1 0.405977607515549 2.59302485675326e-16 postive

RPL3L DLGAP4-AS1 0.502926779469755 1.95990944084855e-25 postive

SCLY DLGAP4-AS1 0.439498629387188 3.8377474827979e-19 postive

RPL3L LINC01397 0.527863150384529 2.72733733432322e-28 postive

CSAD BRWD1-IT1 0.440552016212948 3.08993074291381e-19 postive

PPM1K LINC-PINT 0.508108569877786 5.22238759591514e-26 postive

ASPG DSG1-AS1 0.436738079504438 6.74841600302725e-19 postive

BBOX1 DSG1-AS1 0.784513738876611 2.04968318133981e-79 postive

DUOX1 DSG1-AS1 0.603377330417837 1.47836199857879e-38 postive

SLC6A11 DSG1-AS1 0.591024781737138 1.08716838585049e-36 postive

RPL3L LINC01572 0.471226881608086 3.98647946368533e-22 postive

SCLY LINC01572 0.475300141454397 1.56504866585567e-22 postive

ARG2 LINC01018 0.470467948566724 4.73858502122817e-22 postive

CGA LINC01018 0.725827544428839 1.39760416456525e-62 postive

HAL LINC01018 0.704339082733585 1.79930534032652e-57 postive

RIMKLA LINC01018 0.467876039728027 8.52284201006567e-22 postive

SLC36A4 LINC01018 0.506484113223254 7.92476244258781e-26 postive

CSAD PRC1-AS1 0.448867542917542 5.43616289697361e-20 postive

HYKK PRC1-AS1 0.415413766532748 4.45599652944653e-17 postive

TPH1 UFL1-AS1 0.696764701351879 8.86841612128634e-56 postive

AZIN2 LINC02611 0.420691511907628 1.62370352139289e-17 postive

KMO LINC02611 0.453261943592588 2.1284013098598e-20 postive

CGA SNTG2-AS1 0.559515072021169 2.82601703559521e-32 postive

HAL SNTG2-AS1 0.593400349629042 4.82594455799045e-37 postive

NNMT LINC01235 0.443833093376608 1.56550092432657e-19 postive

PAPSS1 RABGAP1L-IT1 0.41167262570491 9.0174611091586e-17 postive

PSMD1 RABGAP1L-IT1 0.411669495044086 9.02274866654752e-17 postive

RPL34 RABGAP1L-IT1 0.45047002820148 3.86784506579213e-20 postive

AFMID LINC01152 0.678846369137766 5.61262027007779e-52 postive

NAT8L LINC01152 0.757586610383945 4.28829422830694e-71 postive

NAALAD2 LINC02484 0.517607800938414 4.35604303417611e-27 postive

HPD PLAC4 0.493993544682247 1.81843410378603e-24 postive

AASS MIR99AHG 0.422879727707169 1.06279816104949e-17 postive

ASPA MIR99AHG 0.553578162387021 1.70286513421303e-31 postive

BHMT2 MIR99AHG 0.737968996267133 1.08994816042967e-65 postive

CDO1 MIR99AHG 0.617625524589342 8.18719800637494e-41 postive

DIO2 MIR99AHG 0.529514943954368 1.73005746759288e-28 postive

DMGDH MIR99AHG 0.69774067439368 5.40395083919982e-56 postive

INMT MIR99AHG 0.780093696060211 5.72567837905713e-78 postive

MTR MIR99AHG 0.404487745788096 3.40699597667915e-16 postive

PPM1K MIR99AHG 0.494393698572515 1.64803690104878e-24 postive

PSMA5 MIR99AHG -0.44394360551689 1.52985930687112e-19 negative

PSMD14 MIR99AHG -0.486354482144279 1.16078227931527e-23 negative

SERINC1 MIR99AHG 0.426656211937437 5.07729921373433e-18 postive

SLC25A10 MIR99AHG -0.410229636088403 1.18071256055215e-16 negative

SLC25A12 MIR99AHG 0.416476232763467 3.64168466665213e-17 postive

SEM1 RBAKDN 0.552636863359223 2.25642414090014e-31 postive

KMO SKAP1-AS1 0.415796766788783 4.14368869386512e-17 postive

LIPT1 SKAP1-AS1 0.415212105379702 4.62961324856667e-17 postive

PPM1K SKAP1-AS1 0.479220048501506 6.28913228996257e-23 postive

SLC7A5 MYO16-AS1 0.437839290675649 5.39121964933239e-19 postive

AASS FENDRR 0.415011374071253 4.80902388734633e-17 postive

ASPA FENDRR 0.466376781675983 1.19415549696745e-21 postive

BHMT2 FENDRR 0.847166638018986 1.76783867276375e-104 postive

CKB FENDRR 0.417483299238208 3.00567622899361e-17 postive

DMGDH FENDRR 0.64371273779214 2.93609510202856e-45 postive

ENOPH1 FENDRR -0.439765570991967 3.63290666695218e-19 negative

INMT FENDRR 0.606154555742503 5.480799469e-39 postive

MTR FENDRR 0.444112951550797 1.47678696662355e-19 postive

PPM1K FENDRR 0.423509629145343 9.40197978717777e-18 postive

PSMA5 FENDRR -0.42287400982372 1.06398005827867e-17 negative

PSMD14 FENDRR -0.46290944850646 2.58860107983818e-21 negative

SERINC1 FENDRR 0.454000741014636 1.81554446117092e-20 postive

SLC25A10 FENDRR -0.448014889523857 6.51071927857577e-20 negative

SLC25A12 FENDRR 0.599244231144618 6.35822547786695e-38 postive

TPH1 LINC00667 0.589635209447472 1.74287147384332e-36 postive

AHCY MNX1-AS1 0.415967491552477 4.01150235386815e-17 postive

AIMP2 MNX1-AS1 0.480443362857763 4.72041761141087e-23 postive

AASS CADM3-AS1 0.421214102918262 1.46781802600149e-17 postive

ASPA CADM3-AS1 0.577645762141127 9.31868539984458e-35 postive

BHMT2 CADM3-AS1 0.622741150064442 1.18690899361898e-41 postive

DIO2 CADM3-AS1 0.411791162606621 8.81948075064779e-17 postive

DMGDH CADM3-AS1 0.611597625703571 7.61800208157392e-40 postive

INMT CADM3-AS1 0.563774067162938 7.61871197625007e-33 postive

MTR CADM3-AS1 0.409210986675005 1.42705289841607e-16 postive

PPM1K CADM3-AS1 0.544015735275639 2.8520982614189e-30 postive

PSMB5 CADM3-AS1 -0.409184681943476 1.43404074168353e-16 negative

PSMD14 CADM3-AS1 -0.43386529077461 1.20761257518521e-18 negative

SLC25A12 CADM3-AS1 0.425967454219233 5.81359093558401e-18 postive

TPO CADM3-AS1 0.412449878942421 7.794642684828e-17 postive

KMO SIDT1-AS1 0.416192832999354 3.8433533987275e-17 postive

SRM EXOSC10-AS1 0.427918156567264 3.95847550804786e-18 postive

DBH MIAT 0.479581048851814 5.77923373924691e-23 postive

KMO MIAT 0.574598457672791 2.49636308738077e-34 postive

PPM1K MIAT 0.457286119729623 8.91086410819574e-21 postive

ASPA LINC01798 0.434848643562024 9.90125486778999e-19 postive

BHMT2 LINC01798 0.667800673643954 9.12998331382009e-50 postive

CDO1 LINC01798 0.631102144677027 4.67403428582877e-43 postive

DMGDH LINC01798 0.690092554559468 2.48713088619123e-54 postive

INMT LINC01798 0.596647997131597 1.57262283387543e-37 postive

MTR LINC01798 0.401658677316398 5.70010412181154e-16 postive

PPM1K LINC01798 0.438145704646509 5.06395844523049e-19 postive

SLC25A12 LINC01798 0.444767224957949 1.28823559379083e-19 postive

RPL3L LINC02574 0.43733605070722 5.9743983090261e-19 postive

SLC7A5 SCAT1 0.445800201116545 1.03770736852245e-19 postive

BHMT2 ENTPD1-AS1 0.438611471515547 4.60358828844223e-19 postive

DMGDH ENTPD1-AS1 0.517931109795018 3.99738812332018e-27 postive

INMT ENTPD1-AS1 0.4830250474666 2.56657240141751e-23 postive

MTR ENTPD1-AS1 0.428145160872949 3.78471744263583e-18 postive

PPM1K ENTPD1-AS1 0.521370482825665 1.59340626906731e-27 postive

SCLY DLEU1 0.415320822339121 4.53520575419544e-17 postive

SLC25A15 DLEU1 0.513855817544002 1.17282891439913e-26 postive

OAZ2 LINC02525 0.474387828939615 1.93173979654595e-22 postive

CKM PRDM16-DT 0.51363142196511 1.24391750978268e-26 postive

SLC5A5 PRDM16-DT 0.506983946230047 6.97206116357378e-26 postive

IL4I1 LINC01094 0.428251335836626 3.70604259108937e-18 postive

SDS LINC01094 0.519422111051563 2.68639244734221e-27 postive

ASPA ROR1-AS1 0.417178524893267 3.18566083602244e-17 postive

RPL3L GHET1 0.441975349721936 2.30272583391475e-19 postive

AFMID SLCO4A1-AS1 0.631103061584148 4.67235124985432e-43 postive

NAT8L SLCO4A1-AS1 0.546612088485323 1.33879074762043e-30 postive

CSAD C1orf220 0.423810687479777 8.86621288744161e-18 postive

NAALAD2 C1orf220 0.401587288049106 5.77424768554689e-16 postive

PHYKPL C1orf220 0.45751751725686 8.47273870311142e-21 postive

PPM1K C1orf220 0.414712195364425 5.08916956488949e-17 postive

OAZ3 ATXN2-AS 0.400350000059947 7.22032304820368e-16 postive

PSMD4 ATXN2-AS 0.451444865308239 3.14167572278297e-20 postive

RPL3L ATXN2-AS 0.477062445142614 1.04028856401669e-22 postive

CSAD BACH1-IT1 0.443057513997297 1.83968777089943e-19 postive

SEPSECS IQCH-AS1 0.535847569179594 2.95158025104611e-29 postive

GAMT SLC12A5-AS1 0.411491852169795 9.32782140249971e-17 postive

RPL3L SLC12A5-AS1 0.517916772357284 4.01265637475894e-27 postive

TPO MAFA-AS1 0.489291408915708 5.72305813425883e-24 postive

CSAD SDCBP2-AS1 0.406122900716957 2.52471445966895e-16 postive

NAALAD2 SDCBP2-AS1 0.546817106512611 1.26082495718345e-30 postive

SCLY SDCBP2-AS1 0.410692554965985 1.08305962575464e-16 postive

BHMT2 OXCT1-AS1 0.548799264612401 7.04358774636401e-31 postive

DMGDH OXCT1-AS1 0.543338673174739 3.47014226062118e-30 postive

SLC25A12 OXCT1-AS1 0.453446324278182 2.04567843351627e-20 postive

ARG2 LINC02418 0.40647152018869 2.36793240913253e-16 postive

CGA LINC02418 0.456789236245312 9.92844339659179e-21 postive

DBH LINC02418 0.531125694010076 1.10724967559892e-28 postive

HAL LINC02418 0.431522174069969 1.93323231798754e-18 postive

AFMID LINC02313 0.438318896549159 4.887712565217e-19 postive

NAT8L LINC02313 0.425691816464962 6.13681409395256e-18 postive

FAU ZFAS1 0.487297200825005 9.25743942335501e-24 postive

PSMA2 ZFAS1 0.402680398820944 4.73594868558041e-16 postive

RPL10A ZFAS1 0.473998758597141 2.11277496728041e-22 postive

RPL11 ZFAS1 0.578852348057358 6.29018664814814e-35 postive

RPL12 ZFAS1 0.574366663112266 2.68953635419641e-34 postive

RPL13 ZFAS1 0.422727208541171 1.09477042627468e-17 postive

RPL13A ZFAS1 0.483243467410239 2.43703623058538e-23 postive

RPL14 ZFAS1 0.448905065269548 5.39311996418523e-20 postive

RPL18 ZFAS1 0.452242940764268 2.64857599633734e-20 postive

RPL18A ZFAS1 0.410777617705442 1.06599901886811e-16 postive

RPL22 ZFAS1 0.440636143535312 3.03680726512017e-19 postive

RPL23 ZFAS1 0.469346992007349 6.11181943413344e-22 postive

RPL23A ZFAS1 0.528255248740592 2.44857096074498e-28 postive

RPL24 ZFAS1 0.482684072376899 2.78248467259881e-23 postive

RPL26 ZFAS1 0.48749343670776 8.83077549267627e-24 postive

RPL27A ZFAS1 0.547863116887114 9.27740881664237e-31 postive

RPL28 ZFAS1 0.417448187352376 3.02589119578507e-17 postive

RPL29 ZFAS1 0.506688157855547 7.52124525478242e-26 postive

RPL30 ZFAS1 0.568723916897548 1.62108676337876e-33 postive

RPL31 ZFAS1 0.621570036334246 1.85264746995886e-41 postive

RPL32 ZFAS1 0.501605855011211 2.735833137327e-25 postive

RPL34 ZFAS1 0.487836435393185 8.13109952013367e-24 postive

RPL35 ZFAS1 0.456208698249361 1.12629375937918e-20 postive

RPL35A ZFAS1 0.50835664673441 4.89919254488673e-26 postive

RPL36 ZFAS1 0.578169989974597 7.85744157973102e-35 postive

RPL36A ZFAS1 0.531030303183517 1.13697916668639e-28 postive

RPL37 ZFAS1 0.448672875337988 5.66495824026347e-20 postive

RPL37A ZFAS1 0.591051600829945 1.07728622874659e-36 postive

RPL38 ZFAS1 0.488769468751645 6.49270546288635e-24 postive

RPL39 ZFAS1 0.400282413725801 7.3088138342725e-16 postive

RPL5 ZFAS1 0.411922128237509 8.60570407228081e-17 postive

RPL6 ZFAS1 0.487345209022741 9.15121625646999e-24 postive

RPL7 ZFAS1 0.471448271970612 3.79016879401226e-22 postive

RPL7A ZFAS1 0.494785347603044 1.49654575651234e-24 postive

RPL8 ZFAS1 0.469044353696528 6.54545339961796e-22 postive

RPL9 ZFAS1 0.442615930232909 2.01637313557796e-19 postive

RPLP0 ZFAS1 0.526197410425355 4.30517161179296e-28 postive

RPLP1 ZFAS1 0.499591595621685 4.53696419723859e-25 postive

RPLP2 ZFAS1 0.550306262194427 4.51250931521635e-31 postive

RPS10 ZFAS1 0.416642432286678 3.52829134837378e-17 postive

RPS11 ZFAS1 0.469016185175652 6.58732430983995e-22 postive

RPS12 ZFAS1 0.513218070609454 1.3861796595568e-26 postive

RPS13 ZFAS1 0.514269996240231 1.05199365515466e-26 postive

RPS14 ZFAS1 0.409239632020114 1.4194813007253e-16 postive

RPS15 ZFAS1 0.450469887038525 3.86796134636286e-20 postive

RPS15A ZFAS1 0.408101240710753 1.75297684486597e-16 postive

RPS16 ZFAS1 0.417275324104979 3.12737781309689e-17 postive

RPS17 ZFAS1 0.498406981162068 6.09931565993586e-25 postive

RPS18 ZFAS1 0.517582193542773 4.38577173720154e-27 postive

RPS19 ZFAS1 0.505970346009683 9.03787960052154e-26 postive

RPS20 ZFAS1 0.518391054987395 3.53688757943094e-27 postive

RPS21 ZFAS1 0.53218898379225 8.23633175050429e-29 postive

RPS23 ZFAS1 0.412838008715139 7.24654410985462e-17 postive

RPS24 ZFAS1 0.443195344071072 1.78772711030979e-19 postive

RPS25 ZFAS1 0.501470843673737 2.83047670809216e-25 postive

RPS27 ZFAS1 0.42115201831205 1.48553818361699e-17 postive

RPS27A ZFAS1 0.563011364175463 9.64798389493327e-33 postive

RPS28 ZFAS1 0.545501321418986 1.851742157401e-30 postive

RPS29 ZFAS1 0.462340648725217 2.93643476556134e-21 postive

RPS3 ZFAS1 0.477919597939971 8.52139067067247e-23 postive

RPS5 ZFAS1 0.488602474005303 6.7598698764312e-24 postive

RPS7 ZFAS1 0.555260185949192 1.02749073634929e-31 postive

RPS8 ZFAS1 0.530231078026474 1.41912044100757e-28 postive

RPS9 ZFAS1 0.479024565051545 6.58350060124179e-23 postive

UBA52 ZFAS1 0.438828524902467 4.40336526162127e-19 postive

RPL3L DSCR9 0.428826944985129 3.30670411587889e-18 postive

AFMID SOX9-AS1 0.698514701214947 3.64311846956006e-56 postive

NAT8L SOX9-AS1 0.701422208772075 8.19044312027051e-57 postive

PYCR1 SOX9-AS1 0.4139035655087 5.92907024575963e-17 postive

RPL3L MAFTRR 0.433792243577002 1.22552583441667e-18 postive

DBH DBH-AS1 0.830660061718601 6.85200010538951e-97 postive

SARDH DBH-AS1 0.471230483736435 3.98320658408466e-22 postive

PAPSS1 LINC02690 0.46059054883199 4.32167487322445e-21 postive

DARS1 SNHG6 0.404573743874352 3.35385231226558e-16 postive

PSMA2 SNHG6 0.500789092132999 3.36001849293177e-25 postive

RIDA SNHG6 0.403831911090135 3.84039315558619e-16 postive

RPL11 SNHG6 0.515762141481975 7.10154641946287e-27 postive

RPL12 SNHG6 0.495025265313503 1.41062061949729e-24 postive

RPL13 SNHG6 0.410382565272585 1.14753218661078e-16 postive

RPL14 SNHG6 0.439327609225035 3.97491069589897e-19 postive

RPL17 SNHG6 0.412032376867226 8.42969590077371e-17 postive

RPL18 SNHG6 0.403348383809057 4.19414355038252e-16 postive

RPL21 SNHG6 0.403204153617281 4.30572200920523e-16 postive

RPL22 SNHG6 0.494493061129445 1.60822841874113e-24 postive

RPL23 SNHG6 0.44087473509314 2.89098528726266e-19 postive

RPL23A SNHG6 0.431829220755637 1.81800482476827e-18 postive

RPL24 SNHG6 0.528587657358467 2.23443193349345e-28 postive

RPL26 SNHG6 0.534937650578266 3.8142292342964e-29 postive

RPL27A SNHG6 0.544574766994972 2.42485371746571e-30 postive

RPL29 SNHG6 0.438793448481699 4.43513133360887e-19 postive

RPL30 SNHG6 0.655144382928213 2.3998299597998e-47 postive

RPL31 SNHG6 0.633524466974644 1.79739195753845e-43 postive

RPL34 SNHG6 0.514183096750659 1.07628207248462e-26 postive

RPL35 SNHG6 0.492800122151499 2.43671297748489e-24 postive

RPL35A SNHG6 0.458582738130791 6.71447339188301e-21 postive

RPL36 SNHG6 0.537539491435249 1.82853417324536e-29 postive

RPL36A SNHG6 0.522412042885767 1.20353546258508e-27 postive

RPL37 SNHG6 0.484919616898944 1.63577783847886e-23 postive

RPL37A SNHG6 0.529114598342609 1.9322867821908e-28 postive

RPL38 SNHG6 0.504327410558712 1.37388883190995e-25 postive

RPL39 SNHG6 0.519253060398195 2.810493471247e-27 postive

RPL5 SNHG6 0.409772672675274 1.28557275806124e-16 postive

RPL6 SNHG6 0.467159938562305 1.00146789518203e-21 postive

RPL7 SNHG6 0.681617284231777 1.510510642067e-52 postive

RPL7A SNHG6 0.47186635274159 3.4450842999667e-22 postive

RPL8 SNHG6 0.550191813857983 4.6680809693144e-31 postive

RPL9 SNHG6 0.429517954888068 2.88289074954018e-18 postive

RPLP1 SNHG6 0.490049590158264 4.76277926147698e-24 postive

RPLP2 SNHG6 0.498004417502975 6.74279956208608e-25 postive

RPS10 SNHG6 0.413171121760156 6.80646086681587e-17 postive

RPS12 SNHG6 0.469237061816848 6.26595533730562e-22 postive

RPS15 SNHG6 0.427398293976027 4.38648858034831e-18 postive

RPS16 SNHG6 0.407955682258856 1.8008153411039e-16 postive

RPS17 SNHG6 0.458638805463284 6.63262495223872e-21 postive

RPS18 SNHG6 0.451447644556082 3.13981077229791e-20 postive

RPS19 SNHG6 0.479593587899204 5.76227554796371e-23 postive

RPS20 SNHG6 0.680809893167874 2.21755109308683e-52 postive

RPS21 SNHG6 0.42568605179181 6.14375917410118e-18 postive

RPS24 SNHG6 0.45836306952206 7.04484568187439e-21 postive

RPS25 SNHG6 0.412973026437348 7.06489931098686e-17 postive

RPS27 SNHG6 0.446727937574474 8.53984333406698e-20 postive

RPS27A SNHG6 0.533110931816465 6.36701869840335e-29 postive

RPS28 SNHG6 0.453217979949301 2.14860699581943e-20 postive

RPS29 SNHG6 0.461489360828716 3.54468878388244e-21 postive

RPS7 SNHG6 0.590882093917704 1.14127331315761e-36 postive

RPS8 SNHG6 0.515265775408799 8.09500209675866e-27 postive

GLDC CNIH3-AS2 0.518853258994638 3.12696893429083e-27 postive

DMGDH LINC00476 0.451343454312074 3.21047629277685e-20 postive

PPM1K LINC00476 0.443341914697031 1.73405571174045e-19 postive

LIPT1 LINC02453 0.426211532847829 5.54138666390139e-18 postive

RPL3L LINC02453 0.403175138235634 4.3285181753576e-16 postive

DIO1 LINC02532 0.515460874867892 7.68913294542242e-27 postive

ARG2 RNF157-AS1 0.421725337171785 1.3295858985166e-17 postive

ASL KMT2E-AS1 0.42147588167757 1.39535824274201e-17 postive

PSMC2 KMT2E-AS1 0.434738433298919 1.01244015118189e-18 postive

CSAD FMR1-IT1 0.471927659180452 3.39715727708636e-22 postive

AGXT LINC01270 0.587712727615778 3.33589509632705e-36 postive

CSAD ITCH-IT1 0.52043846838014 2.04654247907038e-27 postive

NAALAD2 LINC02327 0.550624673028896 4.10614690756427e-31 postive

RPL3L LINC02327 0.401567501545534 5.79496455193375e-16 postive

HOGA1 LINP1 0.464708077310353 1.73478548806511e-21 postive

GRHPR NAALADL2-AS2 0.566383547275394 3.3805407216948e-33 postive

PSMC1 NAALADL2-AS2 0.474995704333605 1.6790563175238e-22 postive

CSAD CDC42-IT1 0.433765161620811 1.23223329828171e-18 postive

CGA WASHC5-AS1 0.658551156502926 5.5001327209432e-48 postive

HAL WASHC5-AS1 0.728655743006171 2.73206454907395e-63 postive

MTRR WASHC5-AS1 0.40654664651136 2.33541983305192e-16 postive

RIMKLA WASHC5-AS1 0.407916741939294 1.81382910888645e-16 postive

SLC36A4 WASHC5-AS1 0.470668160610706 4.52757545922086e-22 postive

TPO PCCA-AS1 0.459981683551395 4.94100395251947e-21 postive

ASPA A2M-AS1 0.402789002194212 4.64340490856568e-16 postive

BHMT2 A2M-AS1 0.711836985918466 3.35400558463892e-59 postive

CKB A2M-AS1 0.449472951255013 4.78120639465213e-20 postive

DMGDH A2M-AS1 0.619701446486512 3.75532337528586e-41 postive

INMT A2M-AS1 0.539865927201317 9.424085411636e-30 postive

MTR A2M-AS1 0.439448312667702 3.87761208092676e-19 postive

PSMA5 A2M-AS1 -0.409631206935919 1.31985085750376e-16 negative

SLC25A12 A2M-AS1 0.573769708014244 3.25778749928787e-34 postive

IL4I1 SOCAR 0.469468713966914 5.94550713269843e-22 postive

PSMB9 SOCAR 0.416329280828553 3.74492304901762e-17 postive

ASPA FAM66C 0.416751707669082 3.45563356254453e-17 postive

BHMT2 FAM66C 0.465375798871193 1.49435999761164e-21 postive

DMGDH FAM66C 0.490144286041852 4.65461319613238e-24 postive

INMT FAM66C 0.481837317478472 3.39919860494232e-23 postive

MTR FAM66C 0.406492504698368 2.35880643862901e-16 postive

TPO FAM66C 0.501929110750199 2.52173445943362e-25 postive

PHYKPL PABPC4-AS1 0.406994123043174 2.15065026203839e-16 postive

SECISBP2 PABPC4-AS1 0.416655074471929 3.51980931638934e-17 postive

DIO1 LINC01605 0.493020060432207 2.3089635658275e-24 postive

LIPT2 TMPO-AS1 0.47286391816673 2.74186867961012e-22 postive

RPL3L LINC01729 0.580792241733756 3.33229525058779e-35 postive

RPL3L LGR4-AS1 0.486698867106978 1.06878583076598e-23 postive

CSAD PSPC1-AS2 0.468489075928909 7.42135302127192e-22 postive

PHYKPL BET1-AS1 0.419575483630723 2.01308966337928e-17 postive

PSMD1 KIRREL1-IT1 0.405365717800848 2.90117188578175e-16 postive

KMO LINC02328 0.51933926039133 2.74652209743396e-27 postive

PPM1K LINC02328 0.444117597778014 1.47535669484286e-19 postive

PSME4 TRIM52-AS1 -0.410714373545818 1.07865818527932e-16 negative

AIMP1 THAP9-AS1 0.431993523306235 1.75914815193996e-18 postive

ENOPH1 THAP9-AS1 0.50847328846897 4.75413596701419e-26 postive

KYAT3 THAP9-AS1 0.434315498585026 1.10275863170599e-18 postive

ASPA LINC02185 0.480004163354251 5.2333064121884e-23 postive

BHMT2 LINC02185 0.517119071984355 4.95935078531185e-27 postive

DMGDH LINC02185 0.607821014079384 3.00760489602894e-39 postive

INMT LINC02185 0.45797287918853 7.67160884631743e-21 postive

PPM1K LINC02185 0.40347896933987 4.09557186687459e-16 postive

PSMA7 PRANCR 0.484832401430826 1.67015494629335e-23 postive

RPS21 PRANCR 0.537090085036694 2.0770813432007e-29 postive

CSAD LAMC1-AS1 0.424494917786171 7.7576621980699e-18 postive

SCLY LINC00299 0.413558019252586 6.32820319787979e-17 postive

SLC6A7 GAU1 0.45330251732891 2.10991970838904e-20 postive

AMT HNF4A-AS1 0.403269532510275 4.25478757616375e-16 postive

GLS HNF4A-AS1 0.484342943232372 1.87671950325595e-23 postive

GLS LINC00574 0.571392380208939 6.96238127400985e-34 postive

NAALAD2 LINC00574 0.429498148607197 2.89425937125275e-18 postive

BHMT2 HAND2-AS1 0.791635392646094 8.09665791956105e-82 postive

CKB HAND2-AS1 0.427417195279408 4.37015832208197e-18 postive

DMGDH HAND2-AS1 0.627862663875795 1.65590635165966e-42 postive

INMT HAND2-AS1 0.527843145933055 2.74237123175533e-28 postive

MTR HAND2-AS1 0.407554310316024 1.93946798595642e-16 postive

SLC25A12 HAND2-AS1 0.629814881698608 7.74037210143705e-43 postive

IDO1 LINC02195 0.530281948372828 1.39926361837982e-28 postive

IL4I1 LINC02195 0.423035422994647 1.03110699494606e-17 postive

PSMB10 LINC02195 0.426743043085041 4.99124460640183e-18 postive

PSMB9 LINC02195 0.539208320457871 1.13719736541975e-29 postive

PSME1 LINC02195 0.431447169410179 1.9624538417407e-18 postive

PSME2 LINC02195 0.460732507728953 4.18865340883814e-21 postive

SAT1 LINC02195 0.435044855318776 9.51588906424051e-19 postive

SQOR LINC02195 0.409602004952752 1.32703764573512e-16 postive

DMGDH LINC02773 0.417915865521647 2.76726222262189e-17 postive

PHYKPL GLYCTK-AS1 0.45663907895065 1.02578819730733e-20 postive

AFMID SNHG21 0.485326569034136 1.48437699199638e-23 postive

NAT8L SNHG21 0.466859004753594 1.07158525151623e-21 postive

CDO1 PRKAR1B-AS2 0.608683641203233 2.20143032720246e-39 postive

GATM PRKAR1B-AS2 0.816805295220786 3.95706543571536e-91 postive

GNMT PRKAR1B-AS2 0.861473572600361 8.02221908076003e-112 postive

HAO1 PRKAR1B-AS2 0.752322754626859 1.35611279579786e-69 postive

PRODH2 PRKAR1B-AS2 0.820615382858728 1.155394354334e-92 postive

ASPA LINC01081 0.412553076432573 7.64506067913483e-17 postive

BHMT2 LINC01081 0.809576939440881 2.5866905234737e-88 postive

DMGDH LINC01081 0.648062244226105 4.83118930389512e-46 postive

ENOPH1 LINC01081 -0.408922073842869 1.50567067133174e-16 negative

INMT LINC01081 0.612680204969402 5.12155471913753e-40 postive

MTR LINC01081 0.408574704583475 1.60584028858692e-16 postive

PPM1K LINC01081 0.417740370934876 2.861660441533e-17 postive

PSMD14 LINC01081 -0.45129103758481 3.2466169026739e-20 negative

SLC25A12 LINC01081 0.473130483866903 2.57926669102745e-22 postive

SARDH CFAP61-AS1 0.415749037191213 4.18140327866386e-17 postive

TPH1 CFAP61-AS1 0.820018526345321 2.02084132473379e-92 postive

CSAD EDRF1-AS1 0.418782536905489 2.34413608144788e-17 postive

PHYKPL EDRF1-AS1 0.438511691190783 4.69861136385788e-19 postive

IL4I1 LINC02528 0.472215378161735 3.1808658293714e-22 postive

KMO LINC02528 0.444542922626851 1.35004136339357e-19 postive

FTCD LINC02365 0.490438494043172 4.33378095310638e-24 postive

CSAD PLCG1-AS1 0.405171642720471 3.00621501282796e-16 postive

CGA ASAP1-IT2 0.515385422834205 7.84365729690307e-27 postive

HAL ASAP1-IT2 0.563236236867277 8.99968315592021e-33 postive

SLC36A4 ASAP1-IT2 0.448755539024621 5.56666710879511e-20 postive

CSAD ATP11A-AS1 0.410092219822033 1.21132963102338e-16 postive

PSMD1 ATP11A-AS1 0.404076817705902 3.67256397777152e-16 postive

CDO1 NAPA-AS1 0.416691877589567 3.49523085387495e-17 postive

GATM NAPA-AS1 0.492231726957867 2.80005662927831e-24 postive

GNMT NAPA-AS1 0.508245076930712 5.04202668065945e-26 postive

HAO1 NAPA-AS1 0.518619403327533 3.32813828514433e-27 postive

PRODH2 NAPA-AS1 0.468102646613282 8.09808381642714e-22 postive

RPS9 NAPA-AS1 0.409843449168085 1.26875276990701e-16 postive

CKB ADORA2A-AS1 0.432050449300656 1.73919641068439e-18 postive

FTCD ADORA2A-AS1 0.474686369570148 1.80327803194999e-22 postive

GAMT ADORA2A-AS1 0.480537033177073 4.61762054832946e-23 postive

NAALAD2 ADORA2A-AS1 0.47027797219201 4.94775564449022e-22 postive

ASPA CARMN 0.417298852536707 3.11337032509598e-17 postive

BHMT2 CARMN 0.726889875310029 7.58846606468413e-63 postive

DMGDH CARMN 0.597711908785319 1.08615515600722e-37 postive

INMT CARMN 0.658380895716181 5.92306979911298e-48 postive

PPM1K CARMN 0.469614969809897 5.75156431867739e-22 postive

SLC25A12 CARMN 0.580176077918507 4.07927737612325e-35 postive

AANAT LINC00427 0.42095171338508 1.54415460090571e-17 postive

DMGDH LINC00427 0.416506035365368 3.62109103484616e-17 postive

KMO LINC00427 0.457981761143608 7.65674886680735e-21 postive

PPM1K LINC00427 0.426343101209682 5.39989572287679e-18 postive

RPL3L LINC00449 0.467042731807046 1.02822247678826e-21 postive

RPS7 MIR22HG -0.412124800368969 8.28487314013455e-17 negative

LARS1 SNHG4 0.509252155974368 3.88850599320516e-26 postive

SCLY SNHG4 0.477103655191851 1.03037136119741e-22 postive

AFMID LINC01819 0.430278535694492 2.47802527876447e-18 postive

SLC6A7 KRTAP5-AS1 0.504268573686677 1.39459151371185e-25 postive

PSMC1 DLG3-AS1 0.409197871421013 1.43053277169036e-16 postive

CSAD LINC02585 0.400861359633443 6.5840212685183e-16 postive

NAALAD2 FAM230C 0.43610999845902 7.66762750471854e-19 postive

OAZ2 FAM230C 0.411678792956337 9.00705377602742e-17 postive

CSAD LINC01176 0.422354983169126 1.17682149246994e-17 postive

RPL3L LINC02739 0.443298085399787 1.74993676772533e-19 postive

SCLY MCM3AP-AS1 0.501290229768127 2.96215740060692e-25 postive

AFMID OSGEPL1-AS1 0.476434975980283 1.20344339111894e-22 postive

LIPT1 OSGEPL1-AS1 0.41048173255447 1.12650751582148e-16 postive

NAT8L OSGEPL1-AS1 0.44316985654092 1.79722558451384e-19 postive

PSMB9 LINC00944 0.429049408048838 3.16397651426473e-18 postive

GLS SOS1-IT1 0.425806618600115 6.00010075322954e-18 postive

CSAD USP3-AS1 0.515516102072471 7.57793781893702e-27 postive

PHYKPL USP3-AS1 0.435784193824265 8.19188750887001e-19 postive

PIPOX GASAL1 0.409932018271663 1.24800854941998e-16 postive

PRODH BANCR 0.467760487638743 8.74781270094178e-22 postive

SLC25A44 PTOV1-AS1 0.400226976003938 7.38219217585164e-16 postive

AANAT ANKRD44-IT1 0.40891291382921 1.50823158484041e-16 postive

KMO ANKRD44-IT1 0.631056408115988 4.75875295857806e-43 postive

PHYKPL ANKRD44-IT1 0.40901238644026 1.48064898102155e-16 postive

PPM1K ANKRD44-IT1 0.730398047040262 9.89249542260255e-64 postive

SLC3A2 IGFL2-AS1 0.463562713854025 2.23899136927877e-21 postive

KMO LIMS1-AS1 0.445416868741918 1.12451603464538e-19 postive

RPL3L LINC00471 0.493583238965328 2.01119379223039e-24 postive

SEM1 DLX6-AS1 0.566363204681958 3.40211786652451e-33 postive

SLC25A13 DLX6-AS1 0.423337774464272 9.7219507201759e-18 postive

FTCD PANK2-AS1 0.449048128284861 5.23206495138076e-20 postive

NAALAD2 PANK2-AS1 0.462648570987211 2.74278069438925e-21 postive

PSMF1 PANK2-AS1 0.406772866321211 2.24014223761968e-16 postive

SMOX PANK2-AS1 0.405419871002054 2.87251012472148e-16 postive

CSAD HNF1A-AS1 0.480218042874883 4.97702468188555e-23 postive

PHYKPL HNF1A-AS1 0.400565513742918 6.94511426839205e-16 postive

RPL3L ATP2A1-AS1 0.402111179854309 5.25140656314128e-16 postive

AFMID LINC02875 0.442844540472307 1.92291291954766e-19 postive

NAT8L LINC02875 0.406128143420164 2.52228288670768e-16 postive

OAZ2 LINC02875 0.51675550365877 5.46099760001584e-27 postive

PYCR1 LINC02875 0.469236883577844 6.2662083352298e-22 postive

TPH1 LINC02241 0.812171797816474 2.60688932774972e-89 postive

CSAD GEMIN7-AS1 0.402043554073303 5.31619037807025e-16 postive

AFMID SACS-AS1 0.704482527346467 1.66927562568576e-57 postive

NAT8L SACS-AS1 0.799089369010498 1.94951114374815e-84 postive

KMO C1RL-AS1 0.424873598846004 7.2039463410819e-18 postive

PHYKPL C1RL-AS1 0.483074510026212 2.53665605342035e-23 postive

CSAD EFCAB14-AS1 0.436297155714804 7.38154365110394e-19 postive

PHYKPL EFCAB14-AS1 0.422222541067071 1.20745029609809e-17 postive

PSME4 EFCAB14-AS1 0.416102213664347 3.91012404779742e-17 postive

PHYKPL HEXD-IT1 0.424369961453519 7.94939718755392e-18 postive

BCKDK CD2BP2-DT 0.519978312734133 2.31505780061426e-27 postive

NMRAL1 CD2BP2-DT 0.417161479622617 3.19603375905966e-17 postive

SEPHS2 CD2BP2-DT 0.462140817263141 3.06925338174333e-21 postive

ASPG OVOL1-AS1 0.434823317848348 9.95210068398526e-19 postive

BBOX1 OVOL1-AS1 0.415135843879595 4.69698650603352e-17 postive

RPL31 MIR17HG 0.443615311108133 1.63814713827244e-19 postive

SCLY MIR17HG 0.475314277801982 1.55994439802956e-22 postive

FTCD LINC01424 0.462852663399156 2.62141704985404e-21 postive

NAT8L LINC01424 0.482103336419063 3.19218525966088e-23 postive

RPL3L LINC01424 0.491366422827864 3.45815203628633e-24 postive

CSAD LINC01772 0.525005705106521 5.95871264208355e-28 postive

DMGDH LINC01772 0.419857430673031 1.90682511718435e-17 postive

INMT LINC01772 0.429542189829691 2.86903983992023e-18 postive

PHYKPL LINC01772 0.442912708106572 1.89587925983454e-19 postive

PPM1K LINC01772 0.535346238708187 3.3997517871818e-29 postive

AHCY SNHG11 0.476400055511612 1.21322988041583e-22 postive

PSMF1 SNHG11 0.455301237204125 1.37105923591895e-20 postive

SMOX SNHG11 0.443436034086343 1.70043038295424e-19 postive

CSAD INO80-AS1 0.47601433781843 1.32668687195907e-22 postive

TPH1 NPSR1-AS1 0.426091802704504 5.67331037044735e-18 postive

CSAD YEATS2-AS1 0.432081065648852 1.72855802647354e-18 postive

PHYKPL YEATS2-AS1 0.471707762197029 3.57217791162186e-22 postive

CSAD BACH1-IT2 0.415258602330125 4.58900232566033e-17 postive

SECISBP2 SLC25A25-AS1 0.454900862541708 1.49505080872427e-20 postive

AANAT LINC00926 0.40736908087661 2.00693906201701e-16 postive

KMO LINC00926 0.640874775268353 9.38224942449777e-45 postive

PPM1K LINC00926 0.673431205034074 7.0026387783103e-51 postive

AFMID SNHG20 0.565447509870671 4.52827156790188e-33 postive

NAT8L SNHG20 0.457539355154676 8.43250368262597e-21 postive

CSAD RNF213-AS1 0.438645011740082 4.57207374651599e-19 postive

PHYKPL RNF213-AS1 0.503278871794467 1.79274604902534e-25 postive

SERINC3 OSER1-DT 0.409255994464405 1.41517406166653e-16 postive

FTCD LINC00205 0.550529355965397 4.22384174863844e-31 postive

GLS LINC00205 0.434407764683563 1.08240267287936e-18 postive

NAALAD2 LINC00205 0.49053804651756 4.23023918426591e-24 postive

RPL3L LINC00205 0.42648801178395 5.24816489596005e-18 postive

FTCD DSCR4 0.614397344661774 2.71966115590409e-40 postive

NAALAD2 DSCR4 0.432063497196449 1.73465475606127e-18 postive

RPL3L DSCR4 0.424833841098651 7.26020517375863e-18 postive

KMO HLA-DQB1-AS1 0.541554215810122 5.80671920897104e-30 postive

PPM1K HLA-DQB1-AS1 0.413777105271164 6.07218694972622e-17 postive

AHCY SNHG17 0.489363575767904 5.62398419734192e-24 postive

SMOX SNHG17 0.452778310317788 2.3613594661522e-20 postive

SMOX HOXC13-AS 0.407724907750301 1.87929975271176e-16 postive

BHMT2 RXYLT1-AS1 0.456361634752259 1.08951477275362e-20 postive

DMGDH RXYLT1-AS1 0.621956285837076 1.59994621889362e-41 postive

INMT RXYLT1-AS1 0.439054160609752 4.20430933542299e-19 postive

GLDC ANK3-DT 0.471974551878202 3.36094249325998e-22 postive

CSAD IGF2BP2-AS1 0.518485074675862 3.44941621368435e-27 postive

HOGA1 LINC02154 0.405508742190498 2.82607440074461e-16 postive

CSAD CNOT10-AS1 0.422659950018973 1.10916828736015e-17 postive

RPL3L MORC2-AS1 0.412125557715875 8.28369657033165e-17 postive

CSAD MORF4L2-AS1 0.429593823352718 2.83974777323614e-18 postive

LIPT1 MORF4L2-AS1 0.410418138318818 1.13994631767371e-16 postive

PHYKPL MORF4L2-AS1 0.439079547110537 4.18247517958586e-19 postive

PNMT RARA-AS1 0.814277249217959 3.94466220614192e-90 postive

LIPT1 MIR4453HG 0.411565087827614 9.20084294869065e-17 postive

CSAD RASA2-IT1 0.563050911949058 9.53071322394966e-33 postive

LIPT1 MRPS9-AS1 0.420785622623558 1.59447764272398e-17 postive

RPL3L MRPS9-AS1 0.411301556414485 9.6658636366612e-17 postive

IDO1 LINC01480 0.48992886841615 4.90427496058416e-24 postive

KMO LINC01480 0.412388585292366 7.88484294840672e-17 postive

CSAD UBE2R2-AS1 0.433720323673845 1.24341790105949e-18 postive

PSMD1 UBE2R2-AS1 0.525006730822751 5.95704913331171e-28 postive

BHMT2 MBNL1-AS1 0.729733612422452 1.45867869793823e-63 postive

DMGDH MBNL1-AS1 0.545413226757603 1.89989785244201e-30 postive

INMT MBNL1-AS1 0.473419940834625 2.41345663886164e-22 postive

MTR MBNL1-AS1 0.401717923948262 5.63928179081822e-16 postive

SLC25A12 MBNL1-AS1 0.604763873033091 9.01908930111579e-39 postive

IL4I1 LINC02345 0.430805065069138 2.23105692245943e-18 postive

CSAD MCCC1-AS1 0.509073413700963 4.07225082041275e-26 postive

MCCC1 MCCC1-AS1 0.489908661045955 4.92836141778847e-24 postive

PHYKPL MCCC1-AS1 0.438311123226746 4.89549198633919e-19 postive

CSAD ASH1L-AS1 0.484351996765757 1.87267911682043e-23 postive

BHMT2 TPM1-AS 0.577052807774187 1.12973990223392e-34 postive

DMGDH TPM1-AS 0.421276172757072 1.45030978380446e-17 postive

INMT TPM1-AS 0.502265221895337 2.31665681638088e-25 postive

PSMB5 TPM1-AS -0.412737075010251 7.38532304983112e-17 negative

PSMD14 TPM1-AS -0.403589072318545 4.01423101847455e-16 negative

SLC25A12 TPM1-AS 0.436854433160078 6.59045249351592e-19 postive

KMO ADPGK-AS1 0.444977043037682 1.23294607886509e-19 postive

PPM1K ADPGK-AS1 0.450127861856407 4.16004697987652e-20 postive

CSAD KANSL1L-AS1 0.433849571433061 1.21144552911498e-18 postive

LIPT1 KANSL1L-AS1 0.484672666627757 1.73497388549598e-23 postive

PHYKPL KANSL1L-AS1 0.508109702522039 5.22086519089308e-26 postive

AFMID MAFG-DT 0.55567234553162 9.07456357649151e-32 postive

BCKDK MAFG-DT 0.452918058945464 2.2915803860049e-20 postive

NAT8L MAFG-DT 0.556214565368736 7.70431546973233e-32 postive

OAZ2 MAFG-DT 0.412309501001826 8.00274011208438e-17 postive

PYCR1 MAFG-DT 0.724141635977287 3.66225227243575e-62 postive

PYCR2 MAFG-DT 0.403476906008613 4.0971115346874e-16 postive

SLC25A10 MAFG-DT 0.513787857565282 1.19392426759884e-26 postive

AIMP1 SENCR -0.412835376741853 7.25013020578748e-17 negative

ASPA SENCR 0.489574248184524 5.34434005947093e-24 postive

AZIN2 SENCR 0.402683051436197 4.73366690328095e-16 postive

BHMT2 SENCR 0.469632707520857 5.72847131984839e-22 postive

CDO1 SENCR 0.414823632640501 4.98299650952224e-17 postive

DMGDH SENCR 0.473729830069819 2.24758114355798e-22 postive

INMT SENCR 0.530959560176257 1.15953494120317e-28 postive

NNMT SENCR 0.449582094373514 4.67169128118056e-20 postive

PPM1K SENCR 0.488492181168047 6.94223655013181e-24 postive

NMRAL1 LINC01569 0.531783686530174 9.2208865366839e-29 postive

RPL3L LINC01715 0.466739475461833 1.10075862157301e-21 postive

CSAD PLS1-AS1 0.450477895128082 3.86137025690854e-20 postive

KMO LINC01588 0.42761329684882 4.20421932347246e-18 postive

RPS4Y1 ZFY-AS1 0.481901608390011 3.34798657323647e-23 postive

CSAD TIMM23B-AGAP6 0.467188720783254 9.95003455141586e-22 postive

MTR TIMM23B-AGAP6 0.410420998688087 1.13933849414749e-16 postive

PHYKPL TIMM23B-AGAP6 0.471089024908125 4.11374712536172e-22 postive

PPM1K TIMM23B-AGAP6 0.403727018280875 3.91455560694822e-16 postive

PSMB5 TIMM23B-AGAP6 -0.410230066582822 1.18061784855881e-16 negative

PSMB6 TIMM23B-AGAP6 -0.411832562425768 8.75134567921061e-17 negative

PSMD8 TIMM23B-AGAP6 -0.403410867158635 4.14669124510263e-16 negative

SLC7A5 LINC01711 0.456357083846543 1.09059192685227e-20 postive

AIMP2 EMSLR 0.407120692754992 2.10104466430736e-16 postive

RPL8 EMSLR 0.416124020868261 3.89395256233297e-17 postive

BHMT2 PART1 0.731173273532055 6.27903717860403e-64 postive

CKB PART1 0.404311566948692 3.51846230948054e-16 postive

DMGDH PART1 0.568779112751376 1.59311769087945e-33 postive

INMT PART1 0.573409429518324 3.65663176607484e-34 postive

SLC25A12 PART1 0.559036953665627 3.27018619541225e-32 postive

NAALAD2 LINC01762 0.407687247645267 1.89242247185957e-16 postive

EEF1E1 PRRT3-AS1 0.416342319134437 3.73564818137187e-17 postive

SRM PRRT3-AS1 0.428351829593029 3.63305978006809e-18 postive

BHMT2 ZNF710-AS1 0.570881255379253 8.19072930822519e-34 postive

DMGDH ZNF710-AS1 0.490272245194621 4.51229300122691e-24 postive

INMT ZNF710-AS1 0.41103807749752 1.0153845675139e-16 postive

SLC25A12 ZNF710-AS1 0.452376376331786 2.57392541116087e-20 postive

CGA MIR4458HG 0.400197839422758 7.42104718251387e-16 postive

HAL MIR4458HG 0.425499709057611 6.37246798282554e-18 postive

CSAD CFLAR-AS1 0.42943520353653 2.93068271788602e-18 postive

KMO CFLAR-AS1 0.410038561148999 1.22349556351316e-16 postive

PHYKPL CFLAR-AS1 0.462914834679736 2.58550951998258e-21 postive

PPM1K CFLAR-AS1 0.500409274870744 3.69627810103286e-25 postive

CSAD CCDC18-AS1 0.507820577035365 5.62405268788149e-26 postive

KMO CCDC18-AS1 0.42124170775122 1.46000594107479e-17 postive

LIPT1 CCDC18-AS1 0.456602996343327 1.03386362875715e-20 postive

PHYKPL CCDC18-AS1 0.547063616413782 1.1730040024153e-30 postive

PPM1K CCDC18-AS1 0.487746264456845 8.30955345421056e-24 postive

FTCD LINC01410 0.40105164674661 6.36157188801855e-16 postive

NAALAD2 LINC01410 0.413946765843453 5.88094327985286e-17 postive

RPL3L LINC01410 0.451262110952575 3.26673263044871e-20 postive

SCLY PRR7-AS1 0.400638001691704 6.85488205058712e-16 postive

HPD SEPTIN4-AS1 0.434592208327441 1.0428119281157e-18 postive

RPL22 LINC01409 0.423070827234502 1.02403125921705e-17 postive

LIPT1 ELOA-AS1 0.403117726248324 4.37397399513127e-16 postive

BHMT2 SERTAD4-AS1 0.682105751248127 1.19668426081224e-52 postive

DMGDH SERTAD4-AS1 0.510327541399596 2.94369917062612e-26 postive

INMT SERTAD4-AS1 0.6251763815021 4.67385151709736e-42 postive

SLC25A12 SERTAD4-AS1 0.512907059175134 1.50370171517202e-26 postive

PYCR1 MRPL20-AS1 0.421069603712979 1.50938603855584e-17 postive

PYCR2 MRPL20-AS1 0.469006003161818 6.60252415539362e-22 postive

BCKDK FOXD3-AS1 0.421424497848897 1.40929821763783e-17 postive

OAZ2 FOXD3-AS1 0.48197407747636 3.29117184985269e-23 postive

RPL39L FOXD3-AS1 0.406883832868002 2.194811740916e-16 postive

ASPA LINC02256 0.576718672090577 1.2590016860595e-34 postive

BHMT2 LINC02256 0.676225101227239 1.91722540004682e-51 postive

DMGDH LINC02256 0.638984540215606 2.02029265617433e-44 postive

ENOPH1 LINC02256 -0.423680179153718 9.09467809200354e-18 negative

INMT LINC02256 0.697602948341656 5.795929552973e-56 postive

KARS1 LINC02256 -0.404234121510467 3.56858653772528e-16 negative

MTR LINC02256 0.447704770135594 6.95130356773036e-20 postive

PPM1K LINC02256 0.588402852051153 2.64372850896794e-36 postive

PSMA5 LINC02256 -0.46156540327525 3.48565413755769e-21 negative

PSMD14 LINC02256 -0.490098837693814 4.7062205682259e-24 negative

SLC25A12 LINC02256 0.456393545792382 1.08199112291337e-20 postive

TPO LINC02256 0.402083769874037 5.27757072736479e-16 postive

DMGDH LINC01359 0.469862680253481 5.43721192596711e-22 postive

INMT LINC01359 0.444512463110596 1.35865633461907e-19 postive

SEPSECS ITFG1-AS1 0.419725862348732 1.95570834702745e-17 postive

BHMT2 LOH12CR2 0.402473242642237 4.91751871552024e-16 postive

DMGDH LOH12CR2 0.43717359513585 6.17558886330687e-19 postive

CSAD SSBP3-AS1 0.459821353266208 5.11813481367598e-21 postive

PHYKPL SSBP3-AS1 0.471167613351895 4.04071251045134e-22 postive

NAALAD2 LINC02377 0.403774352292625 3.88091656294867e-16 postive

OAZ2 LINC02377 0.416011316257149 3.97824412933595e-17 postive

ASPA MIR29B2CHG 0.501730562574868 2.65119091413451e-25 postive

AANAT DHDDS-AS1 0.417191948586048 3.17751511739478e-17 postive

CSAD DHDDS-AS1 0.514140937311119 1.08826430159914e-26 postive

KMO DHDDS-AS1 0.477308616650451 9.82415254246343e-23 postive

PHYKPL DHDDS-AS1 0.491451595121334 3.38712959765365e-24 postive

PPM1K DHDDS-AS1 0.524408622322545 7.00921643532827e-28 postive

AANAT LINC01550 0.410881217298941 1.04557673071734e-16 postive

ASPA LINC01550 0.40860803285726 1.59595249777924e-16 postive

DMGDH LINC01550 0.44240601582359 2.10611893587945e-19 postive

KMO LINC01550 0.608628599684671 2.24576439501095e-39 postive

PPM1K LINC01550 0.662110291743695 1.15606869467564e-48 postive

RPL3L SBNO1-AS1 0.467067683103592 1.022468511217e-21 postive

BHMT2 RGMB-AS1 0.45551668147705 1.30858831536245e-20 postive

DMGDH RGMB-AS1 0.408686965134587 1.57277279476501e-16 postive

FTCD CCDC144NL-AS1 0.468855952727201 6.83057053499608e-22 postive

PAH HPN-AS1 0.465236171670569 1.54175490471218e-21 postive

RPL3L HPN-AS1 0.424894217352597 7.17493928786244e-18 postive

SLC6A12 HPN-AS1 0.403557192133994 4.03761865474059e-16 postive

RPL3L LINC01433 0.436697501387987 6.80437675291952e-19 postive

SMOX LINC01433 0.424614475706775 7.57846930932441e-18 postive

CSAD LATS2-AS1 0.658311543040951 6.1044454552781e-48 postive

SEM1 TMEM132D-AS1 0.47369165658148 2.26739114413304e-22 postive

SLC3A2 NKILA 0.427396301714355 4.3882133362706e-18 postive

SLC7A5 NKILA 0.406628857089616 2.30034350638627e-16 postive

SMOX NKILA 0.438119531787401 5.0911310657239e-19 postive

RPL38 LINC01023 0.417262863851969 3.13482099744135e-17 postive

CSAD PPP3CB-AS1 0.664110296936373 4.766994062334e-49 postive

CSAD LIX1L-AS1 0.418220566316267 2.61057914753007e-17 postive

DMGDH LIX1L-AS1 0.460199013173936 4.71050756224491e-21 postive

LIPT1 LIX1L-AS1 0.43823431952715 4.97301371270814e-19 postive

PPM1K LIX1L-AS1 0.425811373090679 5.99450385810401e-18 postive

RPL3L LIX1L-AS1 0.401706954722121 5.65049449947985e-16 postive

KMO LINC01857 0.617421607701327 8.83585719332721e-41 postive

PPM1K LINC01857 0.635394942652749 8.54304030779095e-44 postive

CSAD RBM5-AS1 0.490903025175478 3.87108784868966e-24 postive

PHYKPL RBM5-AS1 0.476195142682385 1.27225806101383e-22 postive

PPM1K RBM5-AS1 0.468208138784265 7.90753028583763e-22 postive

APIP CD44-AS1 0.508158967563792 5.15506952892262e-26 postive

PDHX CD44-AS1 0.475947054459692 1.3475223022452e-22 postive

RPS24 CD44-AS1 0.405823096215158 2.66766006874644e-16 postive

GRHPR ARLNC1 0.535812152536632 2.981227143374e-29 postive

PSMC1 ARLNC1 0.456352466574138 1.0916858611233e-20 postive

RPL3L LINC02018 0.406241852699503 2.47010646070692e-16 postive

RPL3L LINC01970 0.517546781050999 4.42721396877988e-27 postive

LIPT1 LINC02367 0.467440643260188 9.40149101708299e-22 postive

PSMA2 LINC02367 0.414362261428015 5.4372259141113e-17 postive

RPL31 LINC02367 0.484519670563438 1.79938200839132e-23 postive

CSAD LINC00893 0.501226229927938 3.01025395096074e-25 postive

PHYKPL LINC00893 0.466335330521706 1.20531451976418e-21 postive

KMO ITGB2-AS1 0.513819763989451 1.18397399232742e-26 postive

PPM1K ITGB2-AS1 0.548920702833757 6.79590693209633e-31 postive

EEF1E1 SNHG12 0.431882741069705 1.79862313333415e-18 postive

RPL11 SNHG12 0.440584658014596 3.06921088646511e-19 postive

RPL22 SNHG12 0.415417507837165 4.45283671941249e-17 postive

RPL31 SNHG12 0.452138876951602 2.70827041519585e-20 postive

SCLY SNHG12 0.42082962378805 1.58099114217671e-17 postive

AASS BVES-AS1 0.414474969964769 5.32264366698053e-17 postive

BHMT2 BVES-AS1 0.745983390411315 7.75196560359657e-68 postive

CKB BVES-AS1 0.54599872732815 1.60163761678962e-30 postive

DMGDH BVES-AS1 0.610127080343161 1.3032089738728e-39 postive

INMT BVES-AS1 0.533435470345078 5.81433442251031e-29 postive

SLC25A12 BVES-AS1 0.517596175545943 4.36951469518841e-27 postive

CKMT1B ATXN1-AS1 -0.400726091140008 6.74677668124075e-16 negative

AASS STARD4-AS1 0.463003501258752 2.53513632437158e-21 postive

DMGDH STARD4-AS1 0.403686313742925 3.94371200769621e-16 postive

MTR STARD4-AS1 0.439163595692972 4.11098065477642e-19 postive

FTCD LINC00954 0.464860518995312 1.67674313873106e-21 postive

GLS LINC00954 0.520194145326588 2.18503780516294e-27 postive

NAALAD2 LINC00954 0.595174872401208 2.61934755449081e-37 postive

ASPA BNC2-AS1 0.453135249650283 2.18714328527013e-20 postive

BHMT2 BNC2-AS1 0.671245385966305 1.91035826051611e-50 postive

CDO1 BNC2-AS1 0.483157926641464 2.4869804268841e-23 postive

DMGDH BNC2-AS1 0.680390217950389 2.70605622945976e-52 postive

ENOPH1 BNC2-AS1 -0.406162978915088 2.50618442546653e-16 negative

INMT BNC2-AS1 0.670552319077504 2.62140852698402e-50 postive

PPM1K BNC2-AS1 0.445997163423158 9.95704128768803e-20 postive

PSMA5 BNC2-AS1 -0.402517723351707 4.87796471733222e-16 negative

PSMD14 BNC2-AS1 -0.425902201216775 5.88856296225631e-18 negative

SLC25A12 BNC2-AS1 0.420289500634412 1.75458825025338e-17 postive

PSMC2 LINC02577 0.429488114036106 2.90003593520476e-18 postive

KMO TSPOAP1-AS1 0.45039512519602 3.93003122202323e-20 postive

PPM1K TSPOAP1-AS1 0.573063781042539 4.08455502887449e-34 postive

ALDH4A1 CAMTA1-IT1 0.444307228924699 1.41813054689237e-19 postive

DBH CAMTA1-IT1 0.442493432853871 2.06827723459751e-19 postive

AANAT LINC02091 0.408796834357292 1.54105741705503e-16 postive

RPL3L LINC01213 0.471310772576421 3.9109399658175e-22 postive

AANAT DPP9-AS1 0.43469783313933 1.0207846800119e-18 postive

CSAD DPP9-AS1 0.513443124594302 1.30684046720692e-26 postive

PHYKPL DPP9-AS1 0.577161355941532 1.09064335782606e-34 postive

PPM1K DPP9-AS1 0.424534679814606 7.69760961000487e-18 postive

CSAD ZMYM4-AS1 0.400128606877332 7.5141787758139e-16 postive

PSMA2 HMBOX1-IT1 0.439428604851881 3.89333685685273e-19 postive

CSAD XIAP-AS1 0.493539396478373 2.03294643822531e-24 postive

AIMP1 LINC00467 0.423292174103741 9.8086341506139e-18 postive

GNMT FLNB-AS1 0.432893599245552 1.46847620895123e-18 postive

PRODH2 FLNB-AS1 0.423196981463734 9.99204487323619e-18 postive

CDO1 C21orf62-AS1 0.439360802592156 3.94791670879596e-19 postive

DMGDH C21orf62-AS1 0.495739380851541 1.18264470914631e-24 postive

INMT C21orf62-AS1 0.409906238941445 1.25401184908041e-16 postive

AANAT C1orf147 0.410184582416945 1.19066606760531e-16 postive

KMO C1orf147 0.419260304364969 2.13877556161637e-17 postive

PHYKPL C1orf147 0.435636401063334 8.4411807348498e-19 postive

SCLY C1orf147 0.484686941143836 1.72908187705571e-23 postive

OAZ1 CYTOR 0.432275569796769 1.66245212030346e-18 postive

PSMA2 CYTOR 0.40802054850332 1.77934045053928e-16 postive

PSMB6 CYTOR 0.414913306434887 4.8991414951303e-17 postive

PSMB9 CYTOR 0.424285440071287 8.08172132280653e-18 postive

AIMP2 FAM13A-AS1 -0.416514278673547 3.6154151279226e-17 negative

CSAD FAM13A-AS1 0.458759684565676 6.45949139795195e-21 postive

KMO FAM13A-AS1 0.468776708751469 6.95411964756276e-22 postive

PHYKPL FAM13A-AS1 0.527166883531262 3.30171815715483e-28 postive

PPM1K FAM13A-AS1 0.523361496086977 9.31110034213477e-28 postive

PSMB5 FAM13A-AS1 -0.406707735166958 2.26717545347451e-16 negative

OAZ2 TBX2-AS1 0.511877792539543 1.96727520570987e-26 postive

ASPA LINC02613 0.433632242827407 1.26568074103382e-18 postive

BHMT2 LINC02613 0.660059045965226 2.84783235323635e-48 postive

CKB LINC02613 0.419087307941081 2.21100757690729e-17 postive

DMGDH LINC02613 0.711892249945734 3.25544438621426e-59 postive

INMT LINC02613 0.555133121827798 1.06756817884541e-31 postive

PPM1K LINC02613 0.468785538821306 6.94024440999773e-22 postive

SLC25A12 LINC02613 0.451168776507133 3.33247827843857e-20 postive

AMDHD1 GAS6-AS1 0.601935171015458 2.46544639824821e-38 postive

SECISBP2 GAS6-AS1 0.455710353992579 1.25482824291669e-20 postive

ASPA LINC02829 0.440444422755711 3.15920655016197e-19 postive

BHMT2 LINC02829 0.476944653843045 1.06915689038785e-22 postive

DMGDH LINC02829 0.545773134427349 1.71063601483548e-30 postive

HDC LINC02829 0.425194427307618 6.76537136035118e-18 postive

KMO LINC01891 0.534081087594358 4.8520053879563e-29 postive

PPM1K LINC01891 0.4455814762009 1.08639699521504e-19 postive

TH CASC8 0.414720508544744 5.08117297404161e-17 postive

CSAD TRMT2B-AS1 0.516368784497939 6.04962746113256e-27 postive

PHYKPL TRMT2B-AS1 0.445965286374754 1.00238673899862e-19 postive

PPM1K TRMT2B-AS1 0.408184848943098 1.72606513158607e-16 postive
