# Supplementary material for: Metabolism-related long non-coding RNA in the stomach cancer associated with 11 AMMLs predictive nomograms for OS in STAD
Source: Front Genet. 2023 Mar 13;14:1127132. doi: 10.3389/fgene.2023.1127132 (PMC10040790; doi:10.3389/fgene.2023.1127132)
Supplement: Supplementary file 6 [file Table3.DOCX]

gene conMean treatMean logFC pValue fdr

FGF14-AS2 1.561544889 0.571523891 -1.450088329 1.00E-08 5.44E-08

LINC01778 0.583953048 0.198911635 -1.553724709 0.00066893 0.000994775

LINC02584 0.02814761 0.170753622 2.600831867 2.00E-08 9.45E-08

SNHG31 0.097293457 0.237149779 1.285383837 5.86E-06 1.43E-05

ETV7-AS1 0.209765656 0.460476387 1.134348684 3.15E-05 6.32E-05

MIR222HG 0.629302525 1.698958917 1.432825329 4.14E-07 1.41E-06

PPP1R35-AS1 0.139601923 0.337531396 1.273702882 2.79E-05 5.65E-05

PRMT5-AS1 0.077927818 0.188617002 1.275249396 4.12E-11 5.41E-10

CSE1L-AS1 0.047109069 0.188790196 2.002707116 1.43E-05 3.15E-05

LINC01697 0.615991533 0.097501512 -2.659414027 7.10E-06 1.68E-05

ADAMTS9-AS2 0.700383261 0.184349404 -1.925701853 5.58E-08 2.33E-07

ZFHX2-AS1 0.087373004 0.175419501 1.005549634 3.34E-07 1.18E-06

LINC00106 0.626847337 1.831010864 1.546454315 6.43E-09 3.67E-08

LACTB2-AS1 0.054043427 0.252784143 2.22571489 1.16E-14 1.34E-12

DENND5B-AS1 0.093498501 0.222284996 1.249395433 1.58E-05 3.44E-05

CCDC28A-AS1 0.075613814 0.180771739 1.257447415 8.78E-08 3.59E-07

LINC02753 0.03107105 0.131834759 2.085087901 2.11E-05 4.38E-05

RBMS3-AS3 0.615873563 0.153403781 -2.005300155 1.71E-08 8.30E-08

TM4SF19-AS1 0.098719496 0.23784503 1.268614954 2.24E-06 5.99E-06

TNFRSF10A-AS1 1.387977907 3.342601158 1.267986618 1.80E-10 1.82E-09

LINC00865 1.039934035 0.514060366 -1.016482327 3.24E-07 1.15E-06

SNHG3 3.440286749 7.536693757 1.131402953 3.31E-09 2.24E-08

ACAP2-IT1 0.223859184 0.610531777 1.44747488 1.64E-08 8.02E-08

LINC02268 0.585717051 0.118275966 -2.308046945 0.020629943 0.021962134

MELTF-AS1 0.639355285 1.876217618 1.553137419 6.33E-12 1.26E-10

HCG15 0.14928589 0.325090444 1.122763336 0.024816463 0.026225457

ZKSCAN2-DT 0.203564565 0.48858042 1.263109597 3.30E-11 4.69E-10

LINC00460 0.055511907 0.650005068 3.54958182 5.21E-10 4.46E-09

DSG2-AS1 0.145152654 0.364016626 1.326433394 4.63E-06 1.16E-05

FBXO36-IT1 0.050515249 0.11277115 1.158607179 0.000141693 0.000245517

N4BP2L2-IT2 0.272937519 0.63262091 1.212770515 2.70E-08 1.25E-07

LINC02762 1.042968725 0.445766123 -1.226337013 1.78E-06 4.90E-06

TFAP2A-AS1 0.183937633 0.561621583 1.610381698 4.28E-07 1.44E-06

LINC01719 0.197809248 0.422140899 1.093614736 3.72E-05 7.34E-05

LINC02633 0.032190917 0.147083266 2.191907523 0.00010071 0.000179907

LINC02575 0.65180256 3.142334119 2.269329664 6.74E-06 1.61E-05

IGBP1-AS1 0.113143947 0.328464733 1.537579078 3.64E-09 2.39E-08

LARS2-AS1 0.043497087 0.136464604 1.649536118 1.49E-06 4.31E-06

BARX1-DT 1.330054102 0.397059384 -1.744058234 0.000273315 0.000439284

LY6E-DT 0.147497115 0.384192542 1.381142781 6.74E-05 0.000126344

CERS3-AS1 0.492847908 0.087911758 -2.487014455 6.76E-06 1.61E-05

MYOSLID 0.078352371 0.52312249 2.739099952 5.30E-09 3.19E-08

LRRK2-DT 0.280865762 0.129110957 -1.12126933 3.52E-06 8.98E-06

CASC20 0.031111125 0.19125074 2.619962883 9.02E-05 0.000164723

LINC00092 0.644352678 0.285326813 -1.175235214 2.73E-10 2.63E-09

FRGCA 0.033018004 0.250155264 2.921498993 5.32E-05 0.000102057

MPRIP-AS1 0.127730601 0.277306799 1.118378788 0.002025226 0.002743088

VPS9D1-AS1 1.516050884 6.909481903 2.18825936 1.51E-12 4.83E-11

KCNQ1OT1 0.140354829 0.526928767 1.908529247 1.32E-08 6.85E-08

LINC00862 0.026116312 0.113069285 2.114183971 6.82E-09 3.86E-08

LINC02542 1.030455649 3.50438046 1.765876999 0.025427984 0.026822571

LYPLAL1-DT 0.244415146 0.096469978 -1.34118175 0.000531015 0.000802083

CAPN10-DT 0.175209571 0.478781636 1.450286232 1.97E-12 5.23E-11

H19 5.772193551 44.04127202 2.931664557 0.001169607 0.001678764

NQO2-AS1 0.048073371 0.110885842 1.205765298 0.000128898 0.000226061

LINC01655 0.050105773 0.448009894 3.160481849 1.08E-08 5.75E-08

FLRT1 1.151437504 0.271722451 -2.08323043 0.000184503 0.000307682

PROX1-AS1 0.017107358 0.108025133 2.658678149 0.023343154 0.024804788

LINC02816 0.033102462 0.114997071 1.796586669 1.35E-06 4.02E-06

TMEM147-AS1 0.512514387 1.101881284 1.104304388 2.21E-09 1.58E-08

ZDHHC20-IT1 0.192868802 0.580398396 1.589423739 4.19E-09 2.69E-08

TMED2-DT 0.167092283 0.418216022 1.323603225 7.36E-13 3.03E-11

ANKRD10-IT1 2.301873964 8.381043432 1.864321027 2.59E-14 2.14E-12

ZNF630-AS1 0.049966704 0.116177355 1.217289928 0.00021374 0.000350364

GTF3C2-AS1 0.11088499 0.286344434 1.368687481 5.96E-11 7.24E-10

SAP30-DT 1.373794922 0.525974226 -1.385102647 0.000591193 0.000883726

LINC01671 0.104906582 0.608007558 2.534984063 0.000807872 0.001180107

LINC02570 0.029850924 0.111442282 1.900449212 0.004056225 0.005087917

SEC62-AS1 0.200767469 0.448364867 1.159147712 1.58E-05 3.44E-05

LINC01004 0.589804638 1.581053635 1.422577238 9.33E-11 9.96E-10

NARF-IT1 0.147778155 0.316000318 1.096492993 3.75E-06 9.49E-06

LINC01579 0.634881404 0.228797022 -1.472418847 0.029718935 0.030786043

CARD11-AS1 0.053445824 0.16948303 1.664991703 1.88E-05 3.98E-05

LINC01484 0.029026775 0.112206059 1.950694397 6.00E-06 1.46E-05

LINC01126 0.130864646 0.308201096 1.235796598 1.28E-07 4.96E-07

NCBP2-AS1 0.150442759 0.329221238 1.129842738 1.11E-06 3.37E-06

LINC01978 0.450300535 1.360067236 1.594717877 3.19E-06 8.18E-06

TTC3-AS1 0.044111274 0.14311087 1.697913932 1.49E-08 7.47E-08

MIR497HG 0.485417798 0.167779838 -1.53265765 2.33E-06 6.17E-06

DSCR8 0.00626333 1.04808904 7.38661577 9.56E-06 2.20E-05

TTTY14 0.930014467 0.410499777 -1.179871721 0.004835347 0.005923557

FOXO6-AS1 0.100095702 0.27397081 1.452642167 0.006643462 0.007887402

KLHL7-DT 0.114441667 0.296373368 1.37280339 0.000551496 0.000830843

LINC02626 0.051640228 0.128900001 1.319684994 0.004704 0.005774911

ODF2-AS1 0.199238165 0.503166797 1.336542693 2.01E-06 5.42E-06

PTOV1-AS2 1.143572334 2.54956184 1.156701709 9.51E-10 7.62E-09

EHMT2-AS1 0.10896961 0.248327352 1.188317326 5.58E-06 1.38E-05

WASIR2 0.107521015 0.331391988 1.623920068 1.02E-05 2.34E-05

LINC01767 0.164571285 0.392037541 1.252279179 0.034732238 0.035850628

LINC01106 0.344080762 0.970041509 1.495299253 4.49E-11 5.75E-10

ADAMTS9-AS1 0.604226531 0.106049932 -2.510345838 0.000457452 0.000703867

STPG3-AS1 0.032342857 0.138636892 2.099792195 1.25E-08 6.58E-08

LINC00592 0.047316226 0.13171675 1.477031913 0.002316296 0.003093757

DNAJC3-DT 0.698001987 1.39642964 1.000439836 9.53E-05 0.000172445

LINC01943 0.35968593 0.757657365 1.074807841 1.11E-08 5.86E-08

MIR548XHG 0.010983015 0.287549594 4.710464748 0.000186227 0.000309663

TSPEAR-AS2 0.205663407 1.136858291 2.466695408 5.67E-09 3.37E-08

LIF-AS1 0.030206456 0.115423924 1.934013444 1.41E-06 4.14E-06

C5orf34-AS1 0.153239323 0.432023933 1.495324677 2.45E-06 6.45E-06

GIHCG 2.173493692 1.072649106 -1.018837702 3.95E-05 7.75E-05

SNHG1 4.370723547 10.93770231 1.323365667 8.23E-12 1.58E-10

FOCAD-AS1 0.070334699 0.154410534 1.134462673 0.004979161 0.006061131

PCAT1 0.054209169 0.272209388 2.328108037 1.19E-12 4.03E-11

TYMSOS 1.013903249 2.265267251 1.159761276 6.74E-06 1.61E-05

TESC-AS1 0.048001825 0.120816275 1.331653652 0.00340989 0.004333715

LINC01537 0.304942145 0.108285226 -1.493699139 1.66E-06 4.62E-06

INE1 0.537190976 1.165724859 1.117720341 1.06E-09 8.23E-09

LINC00278 0.770681513 0.365543112 -1.076093219 0.004400387 0.005436881

TXNDC12-AS1 0.082205216 0.2420184 1.557814887 3.67E-07 1.28E-06

PGM5-AS1 11.18765225 1.104176939 -3.340864035 5.00E-13 2.22E-11

SND1-IT1 0.370363324 0.807106079 1.12381706 2.48E-05 5.09E-05

LINC00337 0.073617501 0.24327384 1.7244605 1.89E-07 7.10E-07

MHENCR 1.698513872 3.720597007 1.131261133 2.57E-10 2.52E-09

AP4B1-AS1 0.220444068 0.50068903 1.183502191 3.59E-08 1.55E-07

RB1-DT 0.069199397 0.149061204 1.107073458 1.63E-05 3.52E-05

LENG8-AS1 1.313684948 2.925826564 1.155224926 6.05E-09 3.49E-08

PIK3CD-AS1 0.059821461 0.149788438 1.324191222 0.000113347 0.000200618

NR2F1-AS1 1.047985487 0.481859441 -1.120934462 0.000670955 0.000995221

LINC02716 0.455530028 0.165545688 -1.460316722 6.11E-12 1.26E-10

LNCAROD 0.01450269 0.341554927 4.557725211 0.010806616 0.012140275

UBOX5-AS1 0.116054148 0.256312224 1.143104204 6.49E-07 2.08E-06

CASC15 0.1177884 0.325207612 1.465163556 3.40E-08 1.49E-07

PRKX-AS1 0.042885537 0.133351571 1.636671741 6.16E-06 1.49E-05

PDC-AS1 0.091997447 0.200907395 1.12686493 2.10E-05 4.37E-05

ITCH-AS1 0.099482777 0.203223344 1.030547449 0.003391023 0.004319251

ELFN1-AS1 0.602013567 5.085259106 3.078453381 2.01E-09 1.47E-08

LINC00894 0.157150316 0.375673422 1.257333877 1.44E-08 7.37E-08

ZNF667-AS1 3.001955924 1.295529017 -1.212361465 4.92E-07 1.65E-06

SLFNL1-AS1 0.130252817 0.273075186 1.067983649 5.11E-09 3.14E-08

TSPEAR-AS1 0.169217301 0.791311169 2.225368039 4.13E-07 1.41E-06

FAM215B 0.057873038 0.132765512 1.197917155 7.44E-05 0.000137648

MMP2-AS1 0.046016452 0.25110671 2.448078921 3.11E-09 2.19E-08

ASMTL-AS1 0.63268346 1.633590034 1.368490184 3.08E-07 1.11E-06

SCAT2 0.170382388 0.82536708 2.276259679 1.17E-13 6.60E-12

LRRC3-DT 0.464345498 0.076525379 -2.601188454 0.003201199 0.00412123

NCOA7-AS1 0.444009678 0.135926698 -1.707762277 0.013917663 0.015209264

THORLNC 0.135213636 0.318325437 1.235261799 7.22E-06 1.70E-05

ARAP1-AS2 0.125447559 0.322725917 1.363225042 6.10E-10 5.01E-09

DIP2A-IT1 0.242912226 0.530465239 1.126823111 1.30E-05 2.89E-05

LINC02696 0.206561517 0.091309001 -1.177742502 0.000523217 0.000792379

MIR924HG 0.161919823 0.476350367 1.556743484 0.000315315 0.000501203

SNHG15 2.08963111 4.521772429 1.113640105 1.13E-12 4.03E-11

LINC01355 0.373998082 1.050755632 1.490324414 8.54E-09 4.70E-08

FOXP1-AS1 0.164191213 0.344088707 1.067403623 0.000258056 0.000415917

LINC02321 0.079860578 0.604235632 2.91955584 1.05E-09 8.23E-09

LINC01311 0.18347997 0.412146964 1.16753629 3.71E-09 2.40E-08

GAS1RR 0.402867178 0.101022303 -1.995630431 3.14E-08 1.43E-07

LINC01979 0.081892694 0.459445172 2.488086054 2.50E-12 6.07E-11

MIR100HG 6.064028876 2.18260537 -1.474225318 0.004891471 0.005966974

LINC02106 1.347980979 0.271948592 -2.309394278 5.92E-07 1.93E-06

MIR1-1HG-AS1 1.566802451 0.293825449 -2.414792029 2.89E-08 1.32E-07

NADK2-AS1 0.238158176 0.481712215 1.016251431 1.21E-05 2.74E-05

LINC01082 4.830414251 1.439264573 -1.746815098 2.64E-12 6.10E-11

C3orf35 0.142473285 0.329659467 1.210285081 3.59E-08 1.55E-07

LINC02473 0.02374379 0.134521267 2.502212109 0.000720502 0.001063247

KCNMB2-AS1 0.02153313 0.915207092 5.409468308 1.03E-12 3.97E-11

UBE2Q1-AS1 0.09804132 0.39111209 1.996120312 2.00E-10 1.99E-09

CTBP1-AS 0.266401002 0.538825493 1.016218601 1.15E-07 4.50E-07

HCP5 10.31810251 20.6694045 1.00231914 9.03E-06 2.09E-05

MIS18A-AS1 0.088976923 0.232208903 1.38392017 1.76E-06 4.88E-06

NARF-AS1 0.07303422 0.236134645 1.692965229 1.09E-09 8.24E-09

ITGB1-DT 0.078158921 0.240842471 1.623607375 4.58E-06 1.15E-05

HHLA3-AS1 0.059939639 0.286350989 2.256202283 5.84E-05 0.000110791

RHPN1-AS1 0.206745637 0.80638304 1.963608416 3.34E-11 4.69E-10

LINC00668 0.911873659 1.948544659 1.095491141 0.001575485 0.002219669

LMF1-AS1 0.071347409 0.146522275 1.038187068 0.0385495 0.039229385

SNHG14 1.106172055 0.436095744 -1.342858985 4.65E-06 1.16E-05

STAG3L5P-PVRIG2P-PILRB 0.227614871 0.584974558 1.36177906 7.04E-11 7.97E-10

KLHL6-AS1 0.189447298 0.394570997 1.058488354 0.007786367 0.009039706

LNCOG 0.017155773 0.175217361 3.35237973 2.14E-15 6.84E-13

ASH1L-IT1 0.108530581 0.328124521 1.596141802 8.54E-07 2.66E-06

HM13-IT1 1.286050015 3.12562734 1.28119903 5.89E-09 3.47E-08

NALT1 3.290001571 0.674121426 -2.287007887 0.000244351 0.000394932

LINC02728 0.076781166 0.172191134 1.165186485 0.000136438 0.000237123

LINC02595 0.467943074 1.782404708 1.929420009 7.34E-11 8.14E-10

DLGAP4-AS1 0.18395959 0.370587892 1.010426858 1.52E-05 3.32E-05

LINC01397 0.046736459 0.154713898 1.726982455 1.07E-07 4.25E-07

LINC01572 0.063080547 0.224692382 1.832684147 2.92E-13 1.41E-11

LINC01018 0.622028941 0.206394233 -1.591579048 1.76E-08 8.47E-08

PRC1-AS1 0.095849083 0.277641808 1.534388293 3.64E-09 2.39E-08

UFL1-AS1 0.049544541 0.165575331 1.740689736 0.000372028 0.000580163

LINC01235 0.098408575 0.855760937 3.120351883 1.86E-14 1.78E-12

RABGAP1L-IT1 0.247407796 0.688663121 1.47690746 7.49E-05 0.000138111

LINC01152 0.038169608 0.165293502 2.114533736 0.038061728 0.038870119

PLAC4 0.191042021 0.629703267 1.720782147 0.009205938 0.010539337

RBAKDN 0.029215101 0.152294821 2.382080706 0.003964632 0.004983862

SKAP1-AS1 0.120883591 0.406316562 1.748985745 5.55E-07 1.84E-06

MYO16-AS1 0.031956125 0.252774302 2.983685411 0.001867299 0.002565313

FENDRR 6.906974516 2.819012419 -1.292864067 3.72E-07 1.28E-06

MNX1-AS1 0.682040898 2.169165529 1.669209992 1.07E-09 8.23E-09

CADM3-AS1 0.239099111 0.111940747 -1.094873483 1.33E-07 5.12E-07

LINC01798 0.305979024 0.11217524 -1.447678484 0.007457653 0.008693062

SCAT1 0.07093738 0.43500215 2.616404585 3.53E-11 4.85E-10

DLEU1 0.381384195 0.773231419 1.019655204 1.34E-11 2.21E-10

LINC02525 0.010518199 0.158111876 3.909986177 0.017032799 0.018404353

PRDM16-DT 3.674984738 0.942899947 -1.962561662 0.000561183 0.000841045

LINC01094 0.231084156 0.728624455 1.656757069 7.04E-11 7.97E-10

GHET1 0.126872655 0.299509333 1.239219802 5.21E-09 3.16E-08

SLCO4A1-AS1 0.58082138 4.561130845 2.973225093 4.56E-08 1.92E-07

ATXN2-AS 0.156685194 0.381352187 1.283255116 1.34E-09 1.00E-08

SLC12A5-AS1 0.038010337 0.175531677 2.207267706 3.07E-10 2.81E-09

MAFA-AS1 0.027187901 0.161243886 2.568207772 0.002021748 0.002743088

LINC02418 0.019209904 0.467560399 4.605230556 9.48E-06 2.19E-05

ZFAS1 19.78309395 40.48185977 1.033007498 6.05E-09 3.49E-08

DSCR9 0.051802638 0.138824932 1.422169211 1.29E-06 3.85E-06

SOX9-AS1 0.060695128 0.146321375 1.269487919 5.68E-05 0.000108111

DBH-AS1 0.16526414 0.446902358 1.435185943 0.000236253 0.000382915

LINC02690 0.015166876 0.332695939 4.455208418 0.002245642 0.003006346

CNIH3-AS2 0.047393171 0.155240839 1.711757045 0.00013628 0.000237123

LINC02532 0.080520538 1.729946831 4.425227084 1.59E-06 4.48E-06

RNF157-AS1 0.14059159 0.345688142 1.297960818 0.002019901 0.002743088

FMR1-IT1 0.198597597 0.554480329 1.481288116 4.13E-08 1.77E-07

LINC01270 0.088125952 0.354619872 2.008634542 1.46E-08 7.41E-08

ITCH-IT1 0.30351287 0.756403033 1.317397454 6.10E-06 1.48E-05

LINC02327 0.008221723 0.121492942 3.885287927 0.040287802 0.040854238

LINP1 0.163769196 0.391420442 1.257055081 0.025599403 0.026954116

NAALADL2-AS2 0.018327475 0.138698071 2.919867773 6.80E-05 0.000127064

CDC42-IT1 0.193310425 0.458442557 1.245821535 1.97E-06 5.34E-06

WASHC5-AS1 0.072278548 0.367961672 2.347916064 4.78E-14 3.45E-12

A2M-AS1 1.376588651 0.492402258 -1.483188238 0.003206988 0.00412123

SOCAR 0.093826498 0.304088529 1.696424073 1.17E-11 2.05E-10

PABPC4-AS1 0.187905326 0.509548202 1.439212671 3.28E-09 2.24E-08

LINC01605 0.239044145 0.878686331 1.878071181 2.87E-06 7.45E-06

TMPO-AS1 0.573480499 1.393954781 1.281367426 6.15E-11 7.24E-10

LGR4-AS1 0.045572478 0.11425076 1.325969036 2.27E-06 6.05E-06

PSPC1-AS2 0.308637282 0.801922119 1.377549787 1.53E-08 7.59E-08

BET1-AS1 0.210010788 0.434791257 1.049859495 2.14E-06 5.74E-06

THAP9-AS1 2.620121483 5.978314365 1.190105057 4.97E-12 1.10E-10

LINC02185 0.296017705 0.141156725 -1.068385605 0.00014731 0.000254484

LINC00299 0.028172902 0.12765188 2.179834685 2.36E-11 3.78E-10

GAU1 1.281203738 2.965782679 1.210912975 0.037387913 0.038249692

LINC00574 0.130475936 0.330711497 1.341789447 0.004523433 0.005576967

HAND2-AS1 5.734787316 0.938003597 -2.612074622 0.000163842 0.000278869

LINC02195 0.223172192 1.054869474 2.24083532 2.11E-08 9.89E-08

PRKAR1B-AS2 0.036579003 0.116772463 1.67461245 0.005640465 0.006822952

LINC01081 0.73044976 0.26517838 -1.461821889 4.41E-08 1.87E-07

CFAP61-AS1 0.016364225 0.289028428 4.142594202 3.11E-07 1.12E-06

LINC02528 0.039333847 0.225363896 2.518413196 5.90E-07 1.93E-06

LINC02365 0.0319375 0.580535507 4.184061019 4.37E-09 2.74E-08

ASAP1-IT2 0.114150693 0.310613276 1.444179879 9.74E-08 3.93E-07

ATP11A-AS1 0.046921625 0.134209207 1.516158763 5.50E-06 1.36E-05

ADORA2A-AS1 0.069284299 0.202500576 1.547325663 0.012605288 0.013933432

CARMN 3.45854586 1.081520855 -1.677104101 0.002738153 0.003582572

LINC00427 0.058415063 0.117736568 1.01115015 0.007932735 0.009172722

LINC00449 0.122774155 0.343472529 1.484187819 3.34E-09 2.24E-08

MIR22HG 10.31419469 4.916958158 -1.068793199 1.86E-12 5.23E-11

SNHG4 0.502799654 1.682071991 1.742183893 1.26E-13 6.60E-12

LINC01819 0.349210024 2.624755302 2.910016053 0.001407548 0.001995467

KRTAP5-AS1 0.287819991 0.665409614 1.20907591 2.53E-05 5.18E-05

LINC02585 0.516323434 1.348673793 1.385194459 2.06E-09 1.49E-08

LINC01176 0.41403894 1.158102792 1.483924946 3.63E-10 3.27E-09

LINC02739 0.058936898 0.118247262 1.004563753 0.001637093 0.002287174

MCM3AP-AS1 0.261934674 0.570446054 1.122883412 1.99E-12 5.23E-11

LINC00944 0.237120412 0.555496725 1.228158547 0.000291535 0.000464685

GASAL1 0.434258038 0.975194436 1.167137344 5.17E-07 1.72E-06

BANCR 0.030912881 2.724615769 6.461700854 6.94E-07 2.21E-06

ANKRD44-IT1 0.161908478 0.402777499 1.314804558 0.00222574 0.002986632

IGFL2-AS1 0.770851419 2.055172122 1.414734513 2.05E-05 4.28E-05

LIMS1-AS1 0.273124864 0.617511317 1.176904921 2.53E-06 6.65E-06

LINC00471 0.138683224 0.323597981 1.222409322 1.66E-05 3.56E-05

DLX6-AS1 0.011532174 0.116465174 3.336162249 3.18E-05 6.35E-05

PANK2-AS1 0.994474918 2.136596587 1.103307647 2.88E-07 1.05E-06

HNF1A-AS1 1.776450234 7.142016275 2.007334148 6.16E-10 5.01E-09

LINC02875 0.137016342 0.337747952 1.301599051 0.000182218 0.000304753

LINC02241 0.008169819 0.210357652 4.686396319 0.001338591 0.00190708

C1RL-AS1 0.414330348 0.992084606 1.259681663 2.88E-10 2.72E-09

EFCAB14-AS1 0.156912242 0.343621846 1.130863851 5.73E-06 1.41E-05

MIR17HG 0.121687046 0.541996635 2.155108297 1.10E-11 1.99E-10

LINC01424 0.046789054 0.130861006 1.48379231 3.08E-06 7.93E-06

INO80-AS1 0.075320322 0.166344549 1.143063519 1.30E-05 2.89E-05

NPSR1-AS1 0.002736597 0.130651604 5.577198283 7.97E-15 1.15E-12

YEATS2-AS1 0.117202478 0.326910332 1.479891902 2.58E-11 4.03E-10

BACH1-IT2 0.088814981 0.220224494 1.310099986 6.83E-08 2.81E-07

SNHG20 0.666217243 1.368660533 1.038700062 6.26E-12 1.26E-10

LINC00205 0.852035728 1.83028598 1.103083253 5.41E-10 4.53E-09

HLA-DQB1-AS1 1.073783002 2.316473639 1.109227793 0.001797928 0.002481059

SNHG17 3.116970885 6.896983594 1.145820856 4.85E-10 4.31E-09

HOXC13-AS 0.009398339 0.228210839 4.601817688 1.74E-05 3.70E-05

IGF2BP2-AS1 0.04663425 0.242223968 2.376879804 1.44E-10 1.48E-09

LINC02154 0.010794506 0.257254634 4.574827978 8.44E-11 9.18E-10

CNOT10-AS1 0.041189334 0.125494766 1.607284476 5.05E-05 9.78E-05

MORF4L2-AS1 0.133569223 0.276153853 1.047884639 1.45E-06 4.23E-06

RASA2-IT1 0.066816256 0.181599568 1.442489728 1.64E-06 4.60E-06

MRPS9-AS1 0.114433201 0.331505443 1.534526868 1.01E-08 5.44E-08

UBE2R2-AS1 0.405436051 0.868556494 1.099145313 1.72E-05 3.68E-05

MBNL1-AS1 3.606294099 1.277836026 -1.496814336 0.001931335 0.002646985

LINC02345 0.030132816 0.179175507 2.571966028 3.88E-11 5.21E-10

MCCC1-AS1 0.15940152 0.42063491 1.399903203 9.32E-08 3.79E-07

ADPGK-AS1 0.074447441 0.155945011 1.066743247 2.30E-07 8.56E-07

KANSL1L-AS1 0.204926434 0.485393523 1.244048762 2.63E-07 9.66E-07

MAFG-DT 1.024926223 2.558968876 1.320042536 1.71E-07 6.50E-07

LINC01569 0.496039316 1.014993483 1.032944087 1.07E-06 3.30E-06

TIMM23B-AGAP6 0.335491365 0.678829162 1.016772908 1.37E-06 4.04E-06

LINC01711 0.008230077 0.308416249 5.227829123 2.82E-15 6.84E-13

EMSLR 0.768452614 3.566140237 2.214335232 3.16E-09 2.19E-08

PART1 1.883196549 0.413810401 -2.186141769 2.78E-11 4.23E-10

LINC01762 0.022564199 0.117082366 2.375416349 1.03E-11 1.91E-10

ZNF710-AS1 7.189913646 3.004435027 -1.258880721 1.06E-06 3.26E-06

LINC01410 0.302118217 0.691922643 1.195497574 0.000369631 0.000579557

PRR7-AS1 0.156991215 0.66217767 2.076534535 8.31E-14 5.33E-12

LINC01409 0.088429392 0.260685842 1.559714349 1.34E-11 2.21E-10

SERTAD4-AS1 7.646535725 3.179701573 -1.265914911 0.00046833 0.000718687

ITFG1-AS1 0.179816746 0.37430461 1.057685434 3.48E-07 1.23E-06

CCDC144NL-AS1 0.069150225 0.502890273 2.862437806 2.61E-07 9.64E-07

HPN-AS1 0.063272389 0.211179848 1.738824205 7.76E-06 1.81E-05

LINC01433 0.046850339 0.168616372 1.847613243 1.39E-08 7.14E-08

TMEM132D-AS1 0.006382564 0.939318677 7.201334709 0.000219785 0.000359252

NKILA 0.303616933 1.401804172 2.206960662 4.57E-09 2.83E-08

CD44-AS1 0.391872426 1.311571623 1.742840627 1.25E-07 4.87E-07

LINC02367 0.065791015 0.151909867 1.207253103 0.000156799 0.000268466

SNHG12 1.527850234 3.488237703 1.190995223 3.16E-11 4.68E-10

BVES-AS1 0.404487178 0.111913061 -1.853715552 6.33E-08 2.63E-07

LINC00954 0.096042959 0.360222854 1.907137961 0.000181083 0.000303736

BNC2-AS1 0.730590207 0.332636932 -1.135114058 0.000173284 0.000292353

LINC02577 0.036023284 0.72814914 4.33723236 3.56E-15 6.84E-13

CAMTA1-IT1 0.024014917 0.118201715 2.299248264 0.003482097 0.004386834

LINC01213 0.049649547 0.105349536 1.085331497 0.038548039 0.039229385

ZMYM4-AS1 0.043231325 0.120463755 1.47845019 2.31E-05 4.77E-05

XIAP-AS1 0.213595801 0.486022848 1.186140854 5.29E-05 0.000101798

C1orf147 0.068578643 0.217871632 1.667647107 2.99E-10 2.79E-09

CYTOR 1.468511498 3.640961646 1.309964982 6.08E-11 7.24E-10

LINC02613 0.335584451 0.121845994 -1.461617059 3.59E-07 1.26E-06

GAS6-AS1 0.364213251 0.941222759 1.369752793 0.001119182 0.001610394

LINC02829 0.224560858 0.108765256 -1.045888704 5.90E-07 1.93E-06

LINC01891 0.057131753 0.149119439 1.384103644 0.00180167 0.002481059

CASC8 0.217285935 0.923515375 2.087541188 1.01E-07 4.01E-07

TRMT2B-AS1 0.126050825 0.408981074 1.698028522 1.25E-06 3.76E-06
