# Supplementary material for: Metabolism-related long non-coding RNA in the stomach cancer associated with 11 AMMLs predictive nomograms for OS in STAD
Source: Front Genet. 2023 Mar 13;14:1127132. doi: 10.3389/fgene.2023.1127132 (PMC10040790; doi:10.3389/fgene.2023.1127132)
Supplement: Supplementary file 7 [file Table4.DOCX]

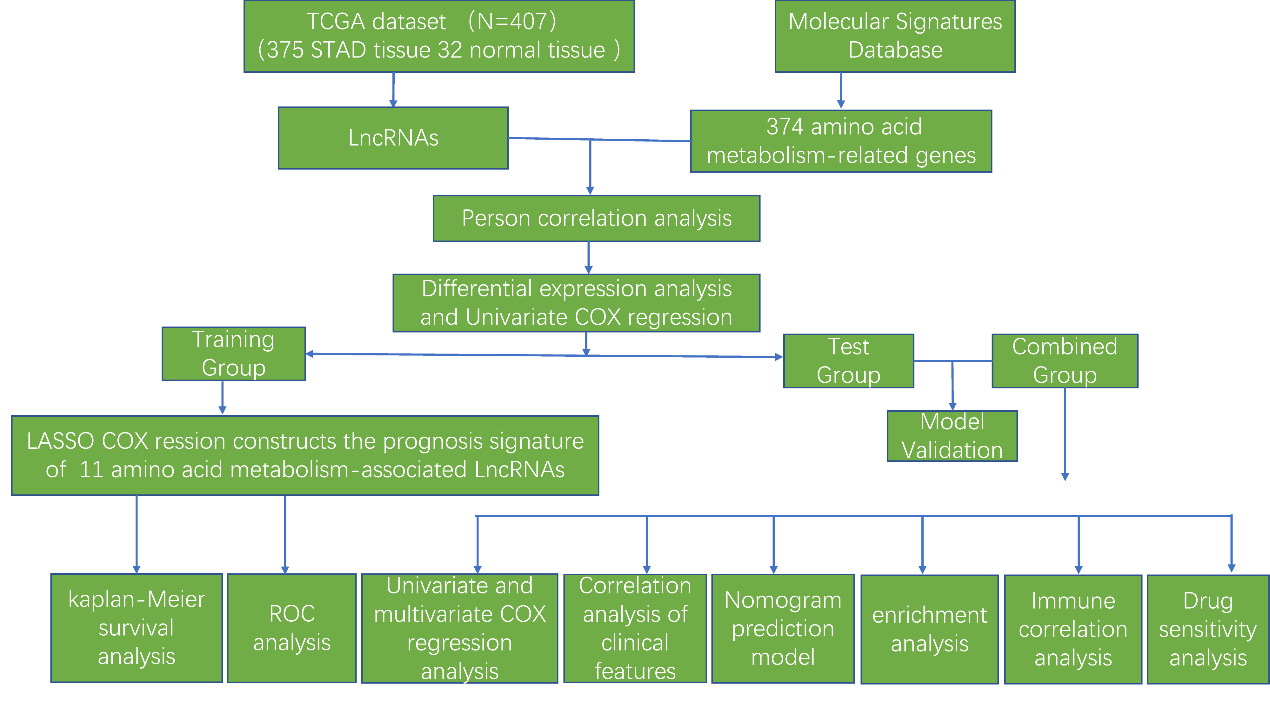


**Supplementary Figure1：**The detailed process of this analysis. TCGA, The Cancer Genome Atlas; STAD, stomach adenocarcinoma; LncRNAs, long non-coding RNAs; LASSO, least absolute shrinkage and selection operator; ROC, receiver operating characteristic.

**
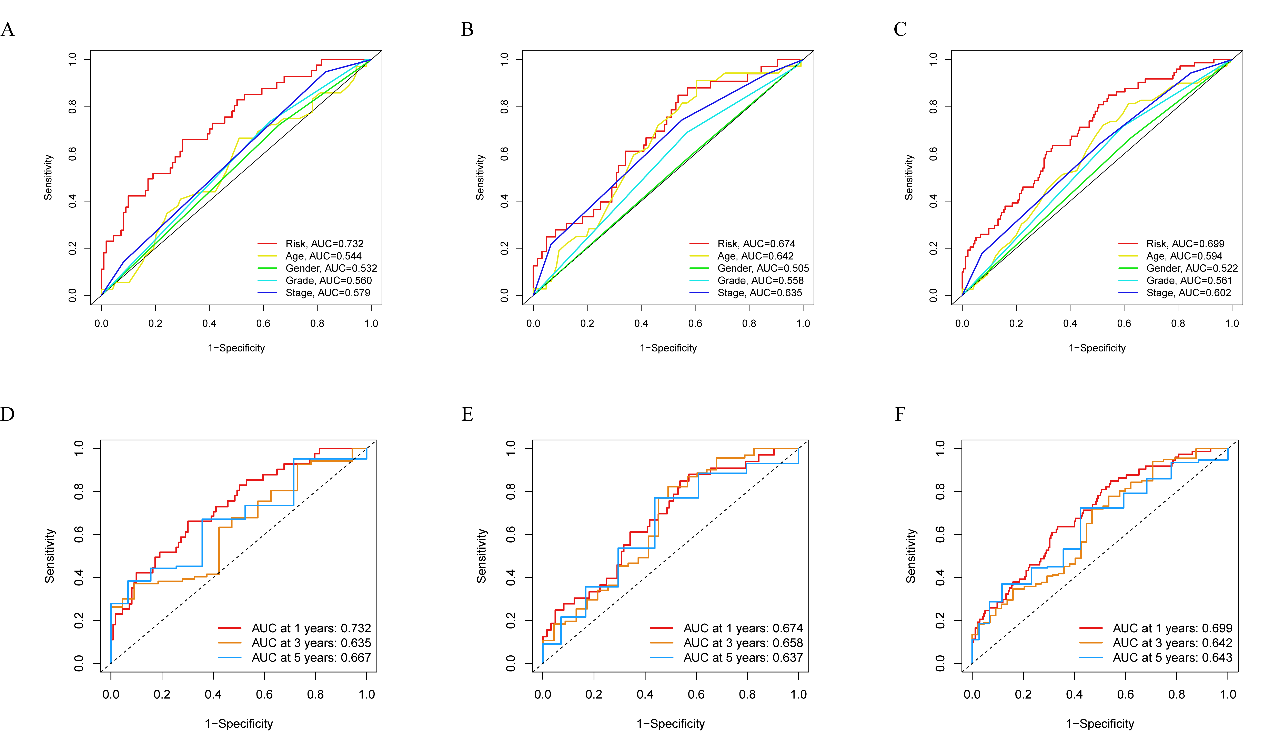
**

**Supplementary Figure2: A-C.** AUC of ROC curves comparing prognostic accuracy of risk scores and other prognostic factors in training, validation and overall groups. **D-F.** ROC curves showed the potential of AMMLs prognostic features in predicting 1-, 2-, and 3-year overall survival (OS) in training, validation, and overall groups. ROC, Receiver operating characteristic; AMMLs, LncRNAs related to amino acid metabolism.


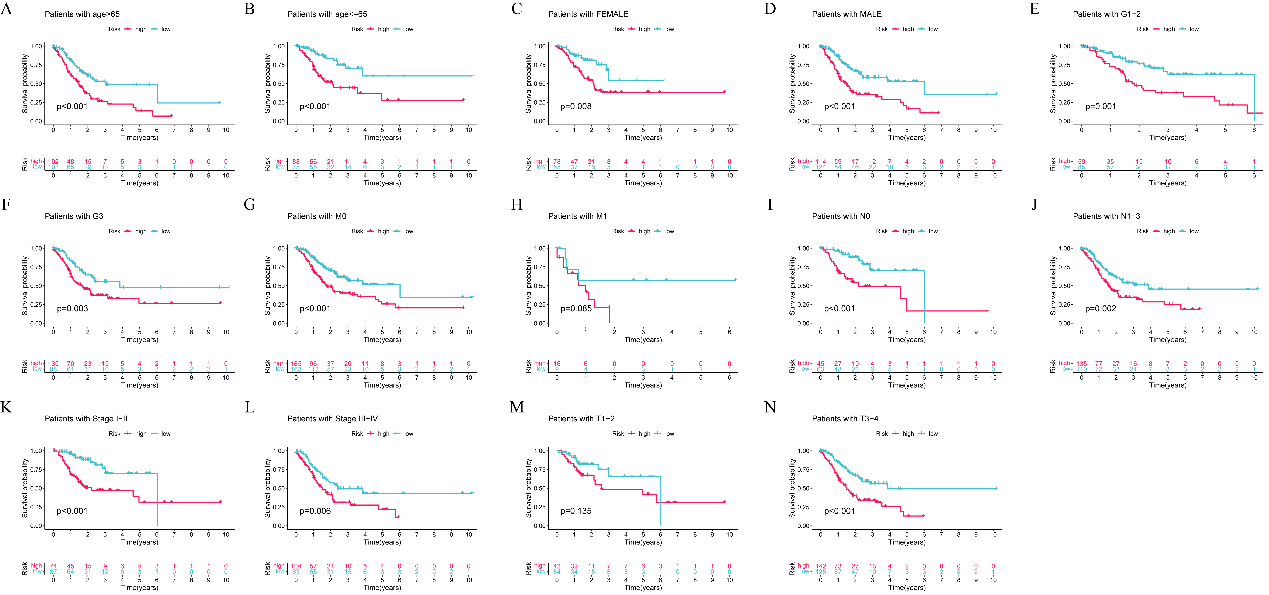


**Supplementary Figure3:** Survival curves stratified between two groups in the entire set by age, sex, grade, stage, T, N, or M. **A.** Age >65 years, **B.** age ≤65 years, **C.** female, **D.** male, **E.** G1–G2, **F.** G3, **G.** M0, **H.** M1, **I.** N0, **J.** N1–N3, **K**. stage I + II, **L.** stage III + IV, **M.** T1–T2. **N.** T3–T4.

**Supplementary Table 1:** A list of amino acid metabolism-related genes.

**Supplementary Table 2:** List of 1724 lncRNAs related to amino acid metabolism.

**Supplementary Table 3:** 327 differential AMMLs between normal and STAD samples. STAD, Stomach adenocarcinoma; AMMLs, LncRNAs related to amino acid metabolism.
